# A comparison of intensive vs. light-touch quality improvement interventions for maternal health in Uttar Pradesh, India

**DOI:** 10.1186/s12913-020-05960-6

**Published:** 2020-12-04

**Authors:** Dominic Montagu, Katie Giessler, Michelle Kao Nakphong, Cathy Green, Kali Prosad Roy, Ananta Basudev Sahu, Kovid Sharma, May Sudhinarset

**Affiliations:** 1 University of California San Francisco, 550 16th St., San Francisco, CA USA; 2 University of California Los Angeles, 650 Charles E. Young Drive South, Los Angeles, CA 90095-1772 USA; 3 Indepedent Improvement Consultant, 24 One Chapmans Peak Drive, Western Cape Hout Bay, South Africa; 4 Population Services International, C-445 Chittranjan Park, New Delhi, 110019 India; 5 Population Services International. Nidhi Kamal Tower, 202, 2nd Floor, Plot – 37 & 38B, Barwara House, Ajmer Road, Jaipur, Rajasthan India; 6 Population Services International, Lucknow, Uttar Pradesh India

**Keywords:** Quality improvement, Person-centered care, Maternal health

## Abstract

**Background:**

Poor patient experiences during delivery leads to delayed presentation at facilities and contributes to poor maternal health outcomes. Person-centered maternity care (PCMC) is a key component of quality. Improving PCMC requires changing the process of care which can be complex and necessitate significant external input, making replication and scale difficult. This study compares the effectiveness two Quality Improvement (QI) intervention phases, one Intensive, one Light-Touch.

**Methods:**

We use a matched case-control design to compare two phases of a QI Intervention targeting PCMC, with three facilities in each. The Intensive phase was introduced into three government facilities where teams were supported to identify, design, and test potential improvements over 12 months. The Light-Touch phase was subsequently introduced in three other government facilities and changes were tracked over six months. We compared the two groups using multivariate linear regression and difference-in-difference models to assess changes in PCMC outcome

**Results:**

Both Intensive and Light-Touch arms demonstrated large improvements in PCMC. On a scale from 0 to 100, Intensive facilities increased in PCMC scores from 85.02 to 97.13, while Light-Touch facilities increased from 63.42 to 87.47. For both there was a ‘halo’ effect, with a similar improvement recorded for the specific improvement activities focused on, as w ell as aspects of PCMC not directly addressed.

**Conclusions:**

This study demonstrates that a short, inexpensive, light-touch and directive intervention can change staff practices and significantly improve the experiences of women during childbirth. It also shows that improvements in a few areas of provider-patient interaction have a ‘halo’ effect, changing many other aspects of patient-provider interaction at the same time.

**Trial registration:**

**QI Phase 1** - NCT04208867. Retrospectively registered. December 19th, 2019.

**QI Phase 2 –** NCT04208841. Retrospectively registered. December 23, 2019.

## Background

The Maternal mortality ratio (MMR) in Indian states remain high. Uttar Pradesh’s MMR is one of the highest in India, reporting 201 deaths per 100,000 live births in 2014–16 [1]. One factor contributing to this high MMR is poor quality care within clinics and hospitals [2]. Poor quality care often deters women from accessing healthcare for both urgent current needs and when post-treatment care or check-ups are indicated; this is true for both poor clinical quality and poor person-centered quality [3–5]. Interventions that improve facility-based care quality are therefore likely to improve utilization of reproductive and intrapartum healthcare and reduce avoidable morbidity and mortality [6]. The importance of person-centered maternity care (PCMC) and client experience is a central component of quality in need of improvement [7].

### Benefits of person-centered maternity care

PCMC includes multiple dimensions of care that patients experience in a facility and the environment in which a woman seeks care. It includes interpersonal interactions, freedom from coercion and abuse, informed and consented care, and provision of respectful care [5, 8]. Higher levels of PCMC are associated with higher patient satisfaction, earlier presentation for care, improved adherence to post-care treatment, and lower overall health care costs [9, 10].

The quality of person-centered maternity care in Uttar Pradesh is low in many settings, especially in public health facilities where 45% of women deliver [11, 12]. Poor person-centered care can delay the recognition of complications, the decision to treat or refer, and limit the amount of information that is shared with a receiving facility, thereby making referrals more complex and generating higher risk of complication for the woman being referred [13]. Poor person-centered care during delivery in Uttar Pradesh has been predicted to have lasting effects on mothers’ decisions about post-partum check-ups, well-baby care, and health seeking for future births [4, 14]. The government has acknowledged the problem of poor person-centered maternal care and prioritized respectful and interpersonal care one key area of focus in a recent high-profile national quality improvement initiative [15]. While this initiative is well funded and has broad support, challenges remain [16, 17]. Effective improvement tools are not known, and in an effort to advance change for maternal health, the National Health Mission has partnered on research to identify possible, and best, practices.

#### Challenge

Recent large-scale studies in Uttar Pradesh have shown both the potential to change some common practices among both clinical and non-clinical staff, and highlighted the high cost and long-term investments needed to effect those changes [18, 19]. The Quality Improvement (QI) literature has documented both the sustained effect, and the gradual change and simplification of interventions over time in hospital settings [20]. Team-based QI interventions demand significant time and resources commitments and so there has been experimentation in many settings with “Light Touch” variations [21–24]. We conducted a study to see if a less-demanding Light Touch intervention could produce improvments in person-centered care similar to a full-intensity QI initiative.

## Methods

In collaboration with the National Health Mission (NHM) of Uttar Pradesh, we conducted a matched case-control QI intervention to improve the PCMC provided to women delivering in government facilities. Study sites were initially identified through previous participation in the “BetterBirth” study [25], a large-scale, clinical quality improvement research intervention. This was done to ensure that all facilities began with an established acceptable level of clinical care provision. Site selection was also informed by the capacity of facility leadership to support a quality improvement intervention focused on PCMC. Inclusion was restricted to facilities averaging more than 100 deliveries per month to ensure recruitment of enough participants to assess baseline to endline changes in a composite PCMC score. We limited selection to a maximum 4-h travel time from the study offices in Lucknow and included rural locations in only two districts, both broadly reflective of the average socio-economic distribution of Uttar Pradesh state. Sites were selected from low- to mid-level facilities: either a Primary Health Center (PHC) or a Community Health Center (CHC). Nine facilities in Unnao and Kanpur Districts met our criteria: three PHCs and six CHCs. Three facilities (two CHCs and one PHC) were randomly selected for the Intensive intervention and three additional facilities, matched based on delivery volume and level of care, were selected for the Light Touch intervention. The final three facilities were retained as controls for a subsequent stage of the study.

### Intervention phases

#### Phase 1: Intensive sites

The three Intensive intervention sites applied the Institute for Healthcare Improvement (IHI) “Improvement Collaborative” model to address PCMC indicators which had been established as priorities through formative studies, and which were determined to be relevant to the facilities from analysis of baseline evaluation performance [26, 27]. In the first phase, the intervention brought representative teams from each facility together every three months to identify potential changes they could introduce to improve. These were subsequently tested by individual teams and revised based on feedback from patients. The team activities were supported by an external advisor who met with each team weekly. The set of process changes which were determined to be effective based on data from exit interviews were collated as a ‘Change Package’ covering eight aspects of PCMC (see below).

#### Phase 2: Light Touch sites

In the second, spread phase, we worked with the facilities that acted as controls during the initial Intensive phase, and encouraged staff to select and introduce relevant process changes from the Change Package developed by teams from the Intensive sites. This Light Touch phase was designed to have limited external input in each facility. At the start of the second phase, representatives from the Light Touch facilities were introduced to the teams who developed the Change Package and heard first-hand of their successes. These staff were then visited for approximately 1.5–2 h every two weeks by the external advisor who had supported the Intensive teams. He provided encouragement for their work, created momentum and responded to any questions. The frequency of these external visits decreased to one per month by the end of the six-month study. Further descriptions of the intervention in the Light Touch sites are available elsewhere [28].

### Data collection

All surveys were developed for this study and are available at https://datadryad.org/stash doi:10.7272/Q6BG2M6W as well as in the supplementary file inventory for this paper. The baseline Intensive site survey was conducted in all facilities between September 2016 and March 2017. The endline for the Intensive sites was conducted in two waves between May and December 2018. In total, 285 women were surveyed at baseline and 300 at endline from three Intensive intervention facilities. At the Light Touch sites, baseline surveys were conducted between April and June 2018 (*n* = 300). Endline surveys were conducted between April and June of 2019 (n = 300). For both Intensive and Light Touch phases, inclusion criteria were women aged 18–49 years who had delivered at the health facility in the last seven days and who were willing and consented to participate. Women who had delivered outside of a participating health facility, were not well enough to participate at the time of recruitment, were less than 18, or who refused to participate following a short explanation about the study purpose were excluded from participating in the survey.

Surveys were conducted using a pre-tested, structured questionnaire. Local investigators were recruited and trained to conduct informed consent and administer the survey via a web-based application. Quality checks (skip patterns, relevance and constraints) were developed in the application and surveys reviewed by the local Research Manager to ensure quality and accuracy. Women who agreed to participate in the study provided verbal consent and each survey took approximately 45 min. All surveys were conducted in person at the health facility by female enumerators, using a tablet-based guide, in the most private setting available.

### Ethics compliance

Human subjects approval for this study was received from the Human Research Protection Program Committee on Human Research of the University of California, San Francisco (IRB# 15–18,008, ref. 176,940; 11/09/2016). Designated approval was received from Population Services International (OHRB Federalwide Assurance (FWA) #0009154)). Formative research was approved from the Institutional Ethics Committee of the Public Health Foundation of India (TRC-IEC-276/15; May 2, 2016).

### Outcome variables

#### Person-centered maternity care Indicator

Person-centered maternity care was assessed using the PCMC scale that measures care received within three domains: dignity and respect; communication and autonomy; and supportive care. This scale was validated using survey data specifically from women who had delivered in Uttar Pradesh and contains 27 items to measure a woman’s PCMC experience at the facility [29]. Four items could not be matched between baseline and endline. Each item asked about frequency of person-centered experiences or care received and scores on individual items ranged from 0 to 3 (0 “No never”; 1 “Yes, a few times”; 2 “Yes, most of the time”; 3 “Yes, all of the time”). Responses that were recorded as “not applicable” were conservatively recoded to receive the highest score. Total PCMC scores were calculated by summing all items for each participant, ranging from zero to 69 points. Final total PCMC and subdomain scores were scaled to 100-point scales.

### Change package indicators

We investigated eight targeted PCMC indicators that were the focus of the Change Package, hereby referred to as “Change Package PCMC score”. These eight indicators include the following: 1.) Provider introduction; 2.) Assurance of visual privacy during exams; 3.) Ability to labour and deliver in the woman’s position of choice; 4.) Cleanliness of toilets/washrooms 5.) Provision of pain medication; 6.) Explanation of medicines and procedures; 7.) Cleanliness of the postnatal ward; and 8.) Assisting the recently delivered woman to the toilet. The latter 3 items were not represented in the PCMC scale, but responded to change ideas prioritized by the facilities and their patients. Total scores for each participant summed all items and could range from zero to 24 points. To assist with interpretability, the eight specific PCMC indicators were also scaled to 100-point scales.

### Non-change package (“halo”) indicators

To examine the impact of the Light Touch intervention on other indicators not targeted by thechange package, we constructed a ‘Non-Change Package PCMC score’ comprising all items in the PCMC scale except the first four items which were included in the Change Package as noted above and described in Table 2. Assessing changes in these indicators was intended to measure a ‘halo effect’, of differences in behaviors indirectly caused by the work on targeted indicators. Total scores could range from zero to 57 points and were also scaled to a 100-point scale.

### Other associated variables

We examined factors that may be associated with PCMC and other outcomes including socioeconomic factors, pregnancy characteristics, and provider characteristics. We investigated the distributions of age, parity, wealth, religion, caste, literacy, education, number of antenatal care (ANC) visits, pregnancy complications, facility type, as well as type and gender of delivery assistant. Wealth was assessed by a modified EquityTool based on India NFHS4 [Released March 30, 2019], equitytool.org, maintained by Metrics for Management.

### Analysis

We conducted three sets of analyses to assess the impact of the Light Touch phase compared to the Intensive phase on 1) total PCMC scores, 2) Change Package PCMC scores that were worked on by facilities and ‘Halo Effect’ indicators, and 3) sub-domains of the total PCMC. Differences between treatment groups at each phase were assessed by cross-tabulations, chi-square tests, and t-tests. We constructed multivariate linear regression difference-in-differences models for each set of analyses to evaluate the impact of the intervention on various outcomes. Our models used the general equation:

$$ Y={\beta}_0+{\beta}_1{X}_{Endline}+{\beta}_2{X}_{L-T}+{\beta}_3{X}_{Endline\ast L-T}+{\beta}_4{X}_{covariates}+\varepsilon $$

For example, in models assessing the impact of the interventions on PCMC scores, the term *β*_1_ estimates the average difference in PCMC score between endline and baseline for the intervention group, *β*_2_ estimates the average difference in PCMC score between Light Touch and Intensive groups at baseline, and *β*_3_ is the interaction term or ‘difference-in-differences estimator’ which estimates the difference in PCMC score slopes between the Light Touch and Intensive groups over time, adjusting for covariates. We tested for homogeneity of variance and used robust standard errors (Eicker-Huber-White) to correct for heteroskedasticity and clustering. Final multivariate models adjusted for age, parity, education, wealth, religion, caste, facility type, delivery provider, number of ANC visits, and pregnancy complications. One key assumption of difference-in-differences models is that groups with and without treatment would follow similar trends. We were unable to assess this empirically because pre-intervention data was collected at only one time point. However, this assumption was considered reasonable because 1) facilities were matched on key characteristics and 2) statistical models adjusted for potential confounders relating to socioeconomic status, health factors, and characteristics of care described above. Because the composition of groups appeared different, we also performed sensitivity analyses using propensity score matching to assess selection effects. These results indicated that our estimates of the interventions appear to be robust (Supplement 1). Stata SE 15.1 was used for all analyses and statistical significance was established at an alpha level of 0.05.

## Results

### Demographic characteristics

No women older than 45 were recruited at any of our sites. At baseline, participants at Light Touch sites had greater wealth and higher education than those at Intensive phase facilities (Table 1). Intensive facilities’ participants attended fewer ANC visits than Light Touch participants at baseline, but more at endline. More participants at Intensive facilities had pregnancy complications than those at Light Touch facilities at baseline, but no significant difference was observed at endline. Across time, deliveries assisted by ANMs, Anganwadi workers and ASHAs (community health workers) increased in Light Touch facilities, whereas nurse, physician and Midwife/Dai assisted deliveries increased at Intensive facilities between survey rounds.

**Table 1:** Characteristics of participants, by Light Touch / Intensive groups and survey round

	Baseline				Endline			
	Light-Touch	Intensive	Total	p	Light-Touch	Intensive	Total	p
**Total number in group**	300	285	585		300	300	600	
**Age** (%)								
18–19 years	8 (2.7%)	6 (2.1%)	14 (2.4%)	0.647	3 (1.0%)	6 (2.0%)	9 (1.5%)	0.524
20–29 years	254 (84.7%)	236 (82.8%)	490 (83.8%)		258 (86.0%)	251 (83.7%)	509 (84.8%)	
30–45 years	38 (12.7%)	43 (15.1%)	81 (13.8%)		39 (13.0%)	43 (14.3%)	82 (13.7%)	
**Number of births** (%)								
1	124 (41.3%)	105 (36.8%)	229 (39.1%)	0.022	92 (30.7%)	122 (40.7%)	214 (35.7%)	0.012
2	98 (32.7%)	95 (33.3%)	193 (33.0%)		110 (36.7%)	79 (26.3%)	189 (31.5%)	
3	53 (17.7%)	39 (13.7%)	92 (15.7%)		65 (21.7%)	57 (19.0%)	122 (20.3%)	
4 or more	25 (8.3%)	46 (16.1%)	71 (12.1%)		33 (11.0%)	42 (14.0%)	75 (12.5%)	
**Wealth Quintiles** (%)								
Poorest	43 (14.3%)	141 (49.5%)	184 (31.5%)	< 0.001	32 (10.7%)	54 (18.0%)	86 (14.3%)	< 0.001
Poorer	65 (21.7%)	47 (16.5%)	112 (19.1%)		38 (12.7%)	53 (17.7%)	91 (15.2%)	
Middle	57 (19.0%)	36 (12.6%)	93 (15.9%)		55 (18.3%)	81 (27.0%)	136 (22.7%)	
Richer	56 (18.7%)	32 (11.2%)	88 (15.0%)		76 (25.3%)	59 (19.7%)	135 (22.5%)	
Richest	79 (26.3%)	29 (10.2%)	108 (18.5%)		99 (33.0%)	53 (17.7%)	152 (25.3%)	
**Religion** (%)								
Hindu	284 (94.7%)	262 (91.9%)	546 (93.3%)	0.203	281 (93.7%)	275 (91.7%)	556 (92.7%)	0.347
Muslim/Other	16 (5.3%)	23 (8.1%)	39 (6.7%)		19 (6.3%)	25 (8.3%)	44 (7.3%)	
**Caste** (%)								
Scheduled Caste	128 (42.7%)	147 (51.6%)	275 (47.0%)	< 0.001	139 (46.3%)	151 (50.3%)	290 (48.3%)	0.339
Scheduled Tribe	1 (0.3%)	7 (2.5%)	8 (1.4%)		2 (0.7%)	5 (1.7%)	7 (1.2%)	
General Caste	48 (16.0%)	75 (26.3%)	123 (21.0%)		39 (13.0%)	29 (9.7%)	68 (11.3%)	
OBC	123 (41.0%)	56 (19.6%)	179 (30.6%)		120 (40.0%)	115 (38.3%)	235 (39.2%)	
**Literate** (%)								
No	34 (11.3%)	88 (30.9%)	122 (20.9%)	< 0.001	23 (7.7%)	60 (20.0%)	83 (13.8%)	< 0.001
Yes	266 (88.7%)	197 (69.1%)	463 (79.1%)		277 (92.3%)	240 (80.0%)	517 (86.2%)	
**Highest grade/class completed** (%)								
No education	39 (13.0%)	88 (30.9%)	127 (21.7%)	< 0.001	30 (10.0%)	61 (20.3%)	91 (15.2%)	< 0.001
Primary or post-primary	112 (37.3%)	117 (41.1%)	229 (39.1%)		136 (45.3%)	151 (50.3%)	287 (47.8%)	
Secondary or higher	149 (49.7%)	80 (28.1%)	229 (39.1%)		134 (44.7%)	88 (29.3%)	222 (37.0%)	
**Number ANC visits** (%)								
No ANC	1 (0.3%)	8 (2.8%)	9 (1.5%)	< 0.001	0 (0.0%)	0 (0.0%)	0 (0.0%)	< 0.001
Less than 4	98 (32.%)	185 (64.9%)	283 (48.4%)		107 (35.7%)	97 (32.3%)	204 (34.0%)	
4 or 5	96 (32.0%)	64 (22.5%)	160 (27.4%)		146 (48.7%)	66 (22.0%)	212 (35.3%)	
6 plus	105 (35.0%)	28 (9.8%)	133 (22.7%)		47 (15.7%)	137 (45.7%)	184 (30.7%)	
**Problems during pregnancy**								
No	184 (61.3%)	84 (29.5%)	268 (45.8%)	< 0.001	209 (69.7%)	212 (70.7%)	421 (70.2%)	0.789
Yes	116 (38.7%)	201 (70.5%)	317 (54.2%)		91 (30.3%)	88 (29.3%)	179 (29.8%)	
**Facility Type** (%)								
Gov’t Health Center	200 (66.7%)	193 (67.7%)	393 (67.2%)	0.786	200 (66.7%)	200 (66.7%)	400 (66.7%)	1.000
Gov’t Hospital	100 (33.3%)	92 (32.3%)	192 (32.8%)		100 (33.3%)	100 (33.3%)	200 (33.3%)	
**Delivery Assistant** (%)								
Nurse/Doctor	112 (37.%)	35 (12.3%)	147 (25.1%)	< 0.001	11 (3.7%)	83 (27.7%)	94 (15.7%)	< 0.001
Midwife/Dai	153 (51.0%)	31 (10.9%)	184 (31.5%)		18 (6.0%)	216 (72.0%)	234 (39.0%)	
ASHA/Angawali	32 (10.7%)	13 (4.6%)	45 (7.7%)		191 (63.7%)	1 (0.3%)	192 (32.0%)	
Other/Non-skilled attendant	3 (1.0%)	206 (72.3%)	209 (35.7%)		80 (26.7%)	0 (0.0%)	80 (13.3%)	
**Gender of delivery** (%) **assistant**								
Male	1 (0.3%)	0 (0.0%)	1 (0.2%)	0.329	0 (0.0%)	1 (0.3%)	1 (0.2%)	0.317
Female	299 (99.7%)	285 (100.0%)	584 (99.8%)		300 (100.0%)	299 (99.7%)	599 (99.8%)	

### Impact of the intervention: Total PCMC score, change package PCMC score, and PCMC sub-domains

Out of a 100-point scale, unadjusted overall mean PCMC score in Light Touch facilities increased 24.03 points from 63.42 (SD 11.44) at baseline to 87.47 (SD 8.31) at endline (Table 2). Mean PCMC score at Intensive facilities increased 12.01 points, from 85.02 (SD 8.12) to 97.13 (SD 2.91).

**Table 2:** Mean PCMC Scores for Full Scale, Change Package and Non-Change Package Indicators, and Sub-Domains, by Light Touch/Intensive groups and survey round^a^

	Baseline						Endline					
	Light-Touch			Intensive			Light-Touch			Intensive		
	N	Mean	SD	N	Mean	SD	N	Mean	SD	N	Mean	SD
**Total PCMC score**												
PCMC total sum (23 indicators)	300	63.42	(11.44)	285	85.02	(8.12)	300	87.47	(8.31)	300	97.13	(2.91)
Change Package PCMC total sum (8 indicators)	300	42.71	(16.15)	285	69.36	(12.56)	300	83.36	(13.51)	300	95.4	(7.61)
Non-Change Package total sum (19 items)	300	68.65	(11.87)	285	91.23	(8.69)	300	88.81	(8.57)	300	98.05	(2.14)
Dignity and Respect domain subtotal (5 indicators)	300	77.42	(15.65)	285	94.41	(9.93)	300	85.33	(10.75)	300	98.22	(3.63)
Communication and Autonomy domain subtotal (7 indicators)	300	40.98	(16.24)	285	78.56	(13.06)	300	84.67	(16.19)	300	96.89	(5.37)
Supportive Care domain subtotal (11 indicators)	300	71.33	(11.57)	285	84.87	(9.82)	300	90.22	(7.24)	300	96.78	(3.00)
**Specific Indicators**												
**Dignity and Respect Domain**												
Treated with respect^b^	300	2.14	(0.76)	285	2.95	(0.28)	300	2.68	(0.53)	300	3	(0.00)
Visual privacy	300	1.64	(1.01)	285	2.44	(1.17)	300	2.77	(0.53)	300	2.99	(0.17)
Record confidentiality^b^	300	2.18	(0.90)	285	2.8	(0.64)	300	1.53	(1.18)	300	2.76	(0.51)
Verbal abuse^b^	300	2.69	(0.68)	285	2.98	(0.17)	300	2.89	(0.49)	300	2.99	(0.11)
Physical abuse^b^	300	2.96	(0.27)	285	3	(0.00)	300	2.92	(0.46)	300	3	(0.00)
**Communication and Autonomy Domain**												
Introduce self	300	0.09	(0.30)	285	0.36	(0.97)	300	1.87	(1.11)	300	2.61	(0.74)
Involvement in care^b^	300	1.08	(1.11)	285	2.82	(0.52)	300	2.49	(0.76)	300	2.93	(0.25)
Delivery position choice	300	1.22	(1.11)	285	2.81	(0.63)	300	2.92	(0.34)	300	2.91	(0.36)
Language^b^	300	2.45	(0.78)	285	2.62	(0.88)	300	2.76	(0.48)	300	2.97	(0.17)
Explain exams/procedures^b^	300	0.68	(0.91)	285	2.83	(0.49)	300	2.49	(0.77)	300	2.94	(0.24)
Explain medicines^b^	300	1.06	(1.24)	285	2.21	(1.19)	300	2.57	(0.72)	300	3	(0.06)
Able to ask questions^b^	300	2.02	(0.91)	285	2.85	(0.55)	300	2.68	(0.51)	300	2.98	(0.15)
**Supportive Care Domain**												
Time to care^b^	300	2.44	(0.78)	285	3	(0.00)	300	2.84	(0.43)	300	2.91	(0.30)
Labor support^b^	300	2.75	(0.57)	285	2.89	(0.41)	300	2.96	(0.20)	300	3	(0.00)
Delivery support^b^	300	2.72	(0.60)	285	2.92	(0.42)	300	2.98	(0.14)	300	3	(0.00)
Attention when need help^b^	300	1.98	(0.80)	285	2.85	(0.47)	300	2.65	(0.49)	300	2.99	(0.10)
Bribes^b^	300	2.28	(0.71)	285	1.35	(1.24)	300	2.65	(0.68)	300	2.55	(0.60)
Control pain^b^	300	1.61	(0.80)	285	2.45	(1.03)	300	2.68	(0.48)	300	2.95	(0.22)
Enough staff^b^	300	1.87	(0.70)	285	2.77	(0.70)	300	2.53	(0.57)	300	2.98	(0.25)
Took best care^b^	300	1.81	(0.62)	285	2.86	(0.46)	300	2.59	(0.50)	300	2.99	(0.11)
Trust^b^	300	2.14	(0.81)	285	2.94	(0.32)	300	2.82	(0.42)	300	2.97	(0.16)
Clean bathroom	300	1.68	(0.62)	285	1.06	(1.29)	300	2.17	(0.51)	300	2.61	(0.51)
Safe^b^	300	2.26	(0.81)	285	2.93	(0.31)	300	2.89	(0.31)	300	2.99	(0.10)
**Change Package**												
Introduction	300	0.09	(0.30)	285	0.36	(0.97)	300	1.97	(1.13)	300	2.61	(0.74)
Privacy	300	1.64	(1.01)	285	2.44	(1.17)	300	2.71	(0.63)	300	2.99	(0.17)
Position of choice	300	1.22	(1.11)	285	2.81	(0.63)	300	2.92	(0.34)	300	2.85	(0.48)
Clean bathroom	300	1.68	(0.62)	285	1.06	(1.29)	300	2.59	(0.76)	300	2.83	(0.57)
Explain test and medicine purpose	300	0.89	(0.97)	285	2.21	(1.19)	300	2.38	(0.92)	300	2.85	(0.51)
Pain medicines given when needed	300	1.27	(0.83)	285	2.45	(1.03)	300	2.29	(0.84)	300	2.99	(0.13)
Helped to the toilet	300	1.31	(1.25)	285	2.68	(0.76)	300	2.41	(1.07)	300	2.88	(0.48)
Clean post-natal care ward	300	2.15	(0.86)	285	2.64	(0.76)	300	2.74	(0.48)	300	2.89	(0.33)

^a^Total, change package, non-change package and subdomain scores were scaled to a 100-point scale

^b^Denotes Non-change package indicator

In Intensive and Light-Touch groups, there was improvement in both the Change Package indicators specifically targeted for change, and the Non-Change Package “Halo” indicators, which were not targeted. In Light-Touch facilities Change-Package PCMC score increased 20.65 points between survey rounds, from 42.71 (SD 16.15) to 83.36 (SD 13.51). In Intensive facilities they increased 26.04 points, from 69.36 (SD 12.56) to 95.4 (SD 7.61).

The Non-Change Package “Halo” indicators increase 20.16 points in Light-Touch facilities (68.65 (SD 11.87) to 88.81 (SD 8.57)); and 6.82 in Intensive facilities (91.23 (SD 8.69) to 98.05 (SD 2.14)).

Subdomains also increased in both Light-Touch and Intensive facilities (Table 2).

**Fig. 1:**
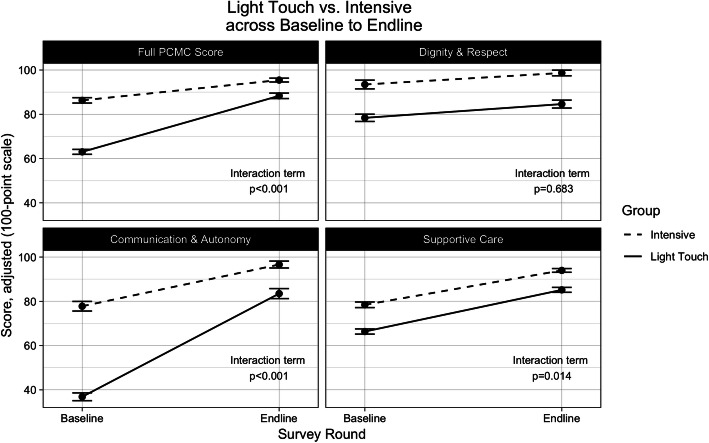
Mean adjusted PCMC scores, by survey round and Light Touch/Intensive sites. Scores were scaled to a 100-point scale. All estimates adjusted for age, parity, education, wealth, religion, caste, facility type, delivery provider, ANC visits, and pregnancy complications. Robust standard errors were used

**Fig. 2:**
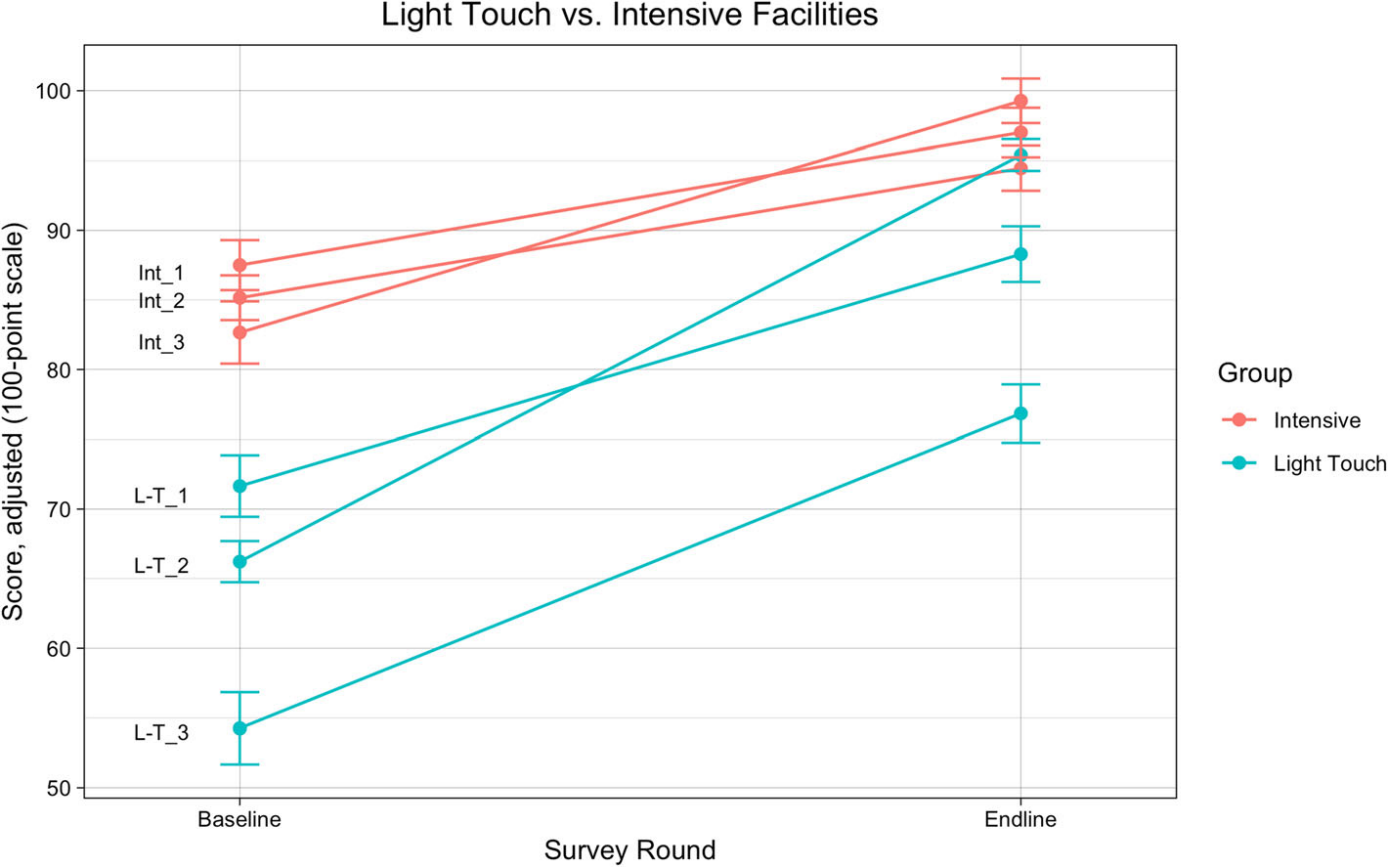
Mean adjusted PCMC scores by facility and survey round. Scores were scaled to a 100-point scale. All estimates adjusted for age, parity, education, wealth, religion, caste, facility type, delivery provider, ANC visits, and pregnancy complications. Robust standard errors were used

Our Difference-in-Difference regression analysis was conducted to adjust for demographic characteristics, facility type, provider factors, and pregnancy complications. The results show the increase in mean total PCMC scores at Light Touch facilities over time was greater than Intensive facilities (Fig. 1). Compared to Intensive intervention facilities, the Light Touch facilities’ adjusted total PCMC scores increased an average of 16.15 points (95% CI: 13.47, 18.83) (Table 3; Table 4). For the Change Package PCMC score, Light Touch facilities increased 11.75 points more (95% CI: 7.33, 16.17) compared to Intensive intervention facilities across survey rounds. Non-change Package score at Light Touch facilities increased 16.79 points (95%CI: 14.12, 19.45) relative to Intensive facilities between survey rounds (Fig. 2).

**Table 3:** Difference-in-Differences analyses of Light Touch vs. Intensive Intervention on PCMC scores. Measured for Full Scale, Change Package, and Non-Change Package items, as well as PCMC Sub-Domains^a^

	Survey Round: Endline (reference Baseline)	Treatment Group: Light Touch (reference Intensive Intervention)	Interaction term
**Full PCMC score, 23 items (unadjusted)**			
Coefficient	12.10	−21.60	11.95
95%CI	(10.76, 13.45)	(−22.95, −20.26)	(10.06, 13.84)
*p*-value	0.000	0.000	0.000
**Full PCMC score, 23 items (adjusted)**			
Coefficient	9.16	−23.27	16.15
95%CI	(7.50, 10.83)	(−25.02, −21.51)	(13.47, 18.83)
*p*-value	0.000	0.000	0.000
**Change Package score, 8 items (adjusted)**			
Coefficient	26.94	−25.52	11.75
95%CI	(24.03, 29.85)	(−28.33, −22.70)	(7.33, 16.17)
*p*-value	0.000	0.000	0.000
**Non-Change Package score, 19 items (adjusted)**			
Coefficient	4.54	−23.70	16.79
95%CI	(2.88, 6.21)	(−25.49, −21.91)	(14.12, 19.45)
*p*-value	0.000	0.000	0.000
**Dignity & Respect (adjusted)**			
Coefficient	5.21	−15.07	1.01
95%CI	(2.26, 8.16)	(−18.05, −12.09)	(−3.84, 5.86)
*p*-value	0.001	0.000	0.683
**Communication and Autonomy (adjusted)**			
Coefficient	18.84	−40.90	27.78
95%CI	(15.77, 21.90)	(−44.01, −37.79)	(22.86, 32.70)
*p*-value	0.000	0.000	0.000
**Supportive Care (adjusted)**			
Coefficient	15.53	−12.07	3.29
95%CI	(13.89, 17.18)	(−13.86, −10.28)	(0.67, 5.92)
*p*-value	0.000	0.000	0.014

^a^Adjusted estimates controlled for age, parity, education, wealth, religion, caste, facility type, delivery provider, ANC visits, and pregnancy complications. Robust standard errors were used

**Table 4:** Mean PCMC Scores for Light Touch / Intensive Facilities, by survey round (unadjusted)^a^

	Baseline						Endline						Unadjusted difference
	Light-Touch			Intensive			Light Touch			Intensive			
	N	Mean	SD	N	Mean	SD	N	Mean	SD	N	Mean	SD	
**Facility**													
L-T_1 PHC	100	70.65	(7.38)				100	89.29	(5.07)				18.64
L-T_2 CHC	100	67.84	(7.19)				100	93.78	(4.44)				25.94
L-T_3 CHC	100	51.77	(8.93)				100	79.33	(7.27)				27.56
Intensive 1 PHC				105	86.87	(6.73)				100	97.67	(2.64)	10.8
Intensive 2 CHC				92	82.14	(10.46)				100	97.23	(3.14)	15.09
Intensive 3 CHC				88	85.84	(5.75)				100	96.48	(2.85)	10.64

^a^*PHC* Primary Health Care center; CHC = Community Health Center

## Discussion

These results contribute to broader QI efforts by demonstrating that a light touch, less intensive QI method may improve women’s experiences of care. Person-centered care, as measured through self-reported patient experience during childbirth, improved significantly at all three facilities which received a year-long, intensively supported team-based collaborative QI intervention. PCMC also improved significantly at all three facilities which subsequently participated in a six-month-long Light Touch phase, with limited and decreasing external support and lower demands on time and input from facility staff and clinicians.

The Intensive facilities, beginning from a higher baseline PCMC score than the Light-Touch facilities, and limited by the upper bound of the scale, were constrained in the absolute potential improvement they could achieve. The similarity of results from both study arms are noteworthy for facilities in low-resource settings where a full QI methodology may not be feasible.

Our findings show only minor differences between the set of activities within the PCMC Scale which are measures of specifically targeted processes included in the “Change Package”, and therefore emphasized for improvement during both Intensive and Light Touch phases, and the remaining 19 indicators within the PCMC scale which measure activities or behaviors not targeted for change. Results indicate that PCMC improved for both Change-Package specific indicators and for non-Change-Package indicators. The increase in PCMC was higher for non-Change Package indicators. The greatest change in PCMC score was for the Communication & Autonomy sub-domain of PCMC. This may be due to the greater ability of providers to change interpersonal behaviors such as calling the patient by their name or introducing themselves as opposed to broader health facility environment changes (i.e. Supportive care sub-domain) or respect and dignity.

This study has a number of limitations. First, there were only a small number of facilities in each arm. We made an effort to match both arms based on existing facility information but we were not able to adjust for clustering effect given the small numbers. However, we used robust standard errors to correct for heteroskedasticity that may have arisen due to facility-level effects. Second, all the facilities had also previously been part of a major quality of care Intervention focused on improving clinical quality for delivery through use of a validated childbirth checklist. As a result, the responsiveness to change interventions among staff may not be reflective of facilities that don’t have this prior experience. Moreover, broader government-focused initiatives on quality improvement may have influenced these results. For example, a government-sponsored, national campaign to improve the cleanliness of public facilities during the period of the intervention may have influenced results in PCMC indicators focused on cleanliness of the washrooms and postnatal wards across both arms. Towards the end of the Intensive phase, the project’s facilities also received attention to a phased expansion of the Government of India’s national initiative to improve the clinical and experiential quality of care in labor rooms and maternity hospitals [15]. This may have influenced the receptiveness of facility leadership to the improvement Interventions in this study, particularly in the light-touch phase. Third, there may have been a Hawthorne effect, through which facility staff changed the intervention activities knowing they would be assessed. We attempted to control for this by measuring aspects of PCMC which were not the target of intervention actions and comparing across the two groups.

## Conclusion

The sustained time commitment by facility staff and clinicians, and the transport and external expertise needed to lead a facility through the team-based Quality Improvement efforts to develop effective process changes are all necessary for effective initiatives. The costs are high, however, and are likely the reason that QI initiatives are increasingly common in OECD countries, and remain rare in Low- and Middle-Income Countries (LMICs). QI interventions do not appear to have changed practices widely in LMICs. We assume that the cost and complexity of implementation is at least part of the reason for this. Added to this already significant barrier, focusing on person-centered care is more challenging than focusing on clinical care in all settings. Where competition for limited resources exists, prioritizing infrastructure and clinical care improvements often takes precedence for both health and political reasons. Our own past research has shown that as providers become overworked and service volumes increase, better clinical care is often at odds with better, more personal, care [30]. Provider stress, and facility management, were shown to be critical contextual pre-conditions for being open to improvement, and anecdotally reported by research teams for this study as well [31].

In this context, our findings are particularly important. This study provides evidence that once effective process changes are identified through a locally developed “Change Package” they can be effectively introduced into facilities without long-duration, high-intensity, support, and that this ‘lighter-touch’, less expensive, method of introduction can achieve similar outcomes. This offers a major opportunity for health systems where demonstrated packages of process changes have been identified to replicate and spread that package of changes widely. In addition, the development of a local change package with its high cost may be more acceptable if there is evidence its benefits can be spread across a much wider system with modest marginal costs.

Beyond this, our research provides insights into an important question about whether PCMC interventions change only specific, targeted, behaviors, or change overall attitudes and practices which effect how providers interact with patients more broadly. This shows that the positive changes in provider and staff practices on actions such as assuring cleanliness and calling patients by name – identified, defined, and targeted for change through collaborative process (Intensive intervention) – spurred more broad-reaching changes in how providers and staff engaged with patients. For example, using the patients’ preferred language when speaking to them, or assuring patients were informed about the intent of interventions or the results of tests. These kinds of actions also improved even though not the focus of change actions identified through the intervention. Because of the small number of facilities involved the findings from this study will need to be replicated to be sure of their broader applicability; nonetheless, they offer a way forward for quality focused intervention efforts. Taken together, these results provide evidence of the potential for improving PCMC in a low-resource setting.

## Supplementary Information

**Additional file 1.**

**Additional file 2.**

**Additional file 3.**

**Additional file 4.**

**Additional file 5.**

**Additional file 6.**

## Data Availability

The datasets generated and/or analyzed during the current study are available at the Dryad repository. https://datadryad.org/stash/dataset/doi:10.7272/Q6BG2M6W

## Abbreviations

ANC: Antenatal Care
CHC: Community Health Center
LMIC: Low- and Middle-Income Countries
MMR: Maternal mortality rates
NHM: National Health Mission
OECD: Organization for Economic Co-operation and Development
PCMC: Person-centered maternity care
PHC: Primary Health Center
QI: Quality Improvement

## References

[1] Office of the Registrar General & Census Commissioner. MMR Bulletin-2014-16.pdf [Internet]. 2018 [cited 2019 Sep 16]. Available from: http://www.censusindia.gov.in/vital_statistics/SRS_Bulletins/MMR%20Bulletin-2014-16.pdf.

[2] Montagu D, Sudhinaraset M, Diamond-Smith N, Campbell O, Gabrysch S, Freedman L (2017). Where women go to deliver: understanding the changing landscape of childbirth in Africa and Asia. Health Policy Plan.

[3] Diamond-Smith N, Sudhinaraset M (2015). Drivers of facility deliveries in Africa and Asia: regional analyses using the demographic and health surveys. Reprod Health.

[4] Bhattacharyya S, Srivastava A, Roy R, Avan BI. Factors influencing women’s preference for health facility deliveries in Jharkhand state, India: a cross sectional analysis. BMC Pregnancy Childbirth. 2016;16(1). 10.1186/s12884-016-0839-6 [cited 2019 Jan 13]. 10.1186/s12884-016-0839-6 PMC4782569 26951787

[5] Sudhinaraset M, Treleaven E, Melo J, Singh K, Diamond-Smith N (2016). Women’s status and experiences of mistreatment during childbirth in Uttar Pradesh: a mixed methods study using cultural health capital theory. BMC Pregnancy Childbirth.

[6] Kruk ME, Kujawski S, Moyer CA, Adanu RM, Afsana K, Cohen J (2016). Next generation maternal health: external shocks and health-system innovations. Lancet.

[7] Tunçalp ӧ, Were W, MacLennan C, Oladapo O, Gülmezoglu A, Bahl R, et al. (2015). Quality of care for pregnant women and newborns-the WHO vision. BJOG Int J Obstet Gynaecol.

[8] Afulani PA, Diamond-Smith N, Golub G, Sudhinaraset M. Development of a tool to measure person-centered maternity care in developing settings: validation in a rural and urban Kenyan population. Reproductive Health. 2017;14(1) [cited 2018 Jul 4]. 10.1186/s12978-017-0381-7. 10.1186/s12978-017-0381-7 PMC5610540 28938885

[9] Raj A, Dey A, Boyce S, Seth A, Bora S, Chandurkar D (2017). Associations between mistreatment by a provider during childbirth and maternal health complications in Uttar Pradesh. India Maternal Child Health J.

[10] Sudhinaraset M, Landrian A, Afulani PA, Diamond-Smith N, Golub G (2019). Association between person-centered maternity care and newborn complications in Kenya. Int J Gynecol Obstet..

[11] National Family Health Survey, India. NFHS-4. Mumbai: International Institute for Population Sciences; 2014. Available from: http://rchiips.org/nfhs/nfhs4.shtml. Accessed 28 Nov 2020.

[12] Sharma G, Penn-Kekana L, Halder K, Filippi V (2019). An investigation into mistreatment of women during labour and childbirth in maternity care facilities in Uttar Pradesh, India: a mixed methods study. Reprod Health.

[13] Singh S, Doyle P, Campbell OM, Mathew M, Murthy GVS (2016). Referrals between Public Sector Health Institutions for Women with Obstetric High Risk, Complications, or Emergencies in India – A Systematic Review. Ho Y-S, editor. PLoS ONE.

[14] Sudhinaraset M, Beyeler N, Barge S, Diamond-Smith N (2016). Decision-making for delivery location and quality of care among slum-dwellers: a qualitative study in Uttar Pradesh. India BMC Pregnancy Childbirth.

[15] National Health Systems Resource Center. LaQshya Labor Room Quality Improvement Initiative Guideline [Internet]. LaQysha. [cited 2018 Jul 1]. Available from: http://nhsrcindia.org/updates/laqshya-लक्ष्य-labour-room-quality-improvement-initiative-guideline.

[16] Srivastava A, Singh D, Montagu D, Bhattacharyya S (2018). Putting women at the center: a review of Indian policy to address person-centered care in maternal and newborn health, family planning and abortion. BMC Public Health.

[17] O’Neil S, Naeve K, Ved R. An Examination of the Maternal Health Quality of Care Landscape in India. Cambridge: Mathematica Policy Research, 2017. Available from https://www.macfound.org/media/files/50268_Landscape_Report_2017.03.02.pdf. Accessed 28 Nov 2020.

[18] Semrau KEA, Hirschhorn LR, Marx Delaney M, Singh VP, Saurastri R, Sharma N (2017). Outcomes of a coaching-based WHO safe childbirth checklist program in India. N Engl J Med.

[19] Barnhart DA, Spiegelman D, Zigler CM, Kara N, Marx Delaney M, Kalita T, et al. Coaching Intensity, Adherence to Essential Birth Practices, and Health Outcomes in the BetterBirth Trial in Utter Pradesh, India. Glob Health Sci Pract. 2020;8(1):38–54. 10.9745/GHSP-D-19-00317 PMC7108945 32127359

[20] Robert G, Sarre S, Maben J, Griffiths P, Chable R (2020). Exploring the sustainability of quality improvement interventions in healthcare organisations: a multiple methods study of the 10-year impact of the ‘productive Ward: releasing time to care’ programme in English acute hospitals. BMJ Qual Saf.

[21] Barnhart DA, Spiegelman D, Zigler CM (2020). Coaching Intensity, Adherence to Essential Birth Practices, and Health Outcomes in the Better Birth Trial in Uttar Pradesh. India. Glob Health Sci Pract..

[22] Stephens TJ, Peden CJ, Pearse RM, Shaw SE, TEF A, on behalf of the EPOCH trial group (2018). Improving care at scale: process evaluation of a multi-component quality improvement intervention to reduce mortality after emergency abdominal surgery (EPOCH trial). Implementation Sci.

[23] Stephens T, Johnston C, Hare S (2019). Quality improvement and emergency laparotomy care: what have we learnt from recent major QI efforts?. Clin Med.

[24] El-Khani A, Maalouf W, Baker DA, Zahra N, Noubani A, Cartwright K (2020). Caregiving for children through conflict and displacement: a pilot study testing the feasibility of delivering and evaluating a light touch parenting intervention for caregivers in the West Bank. Int J Psychol.

[25] Gawande AA, Semrau K, Miller K, Lipsitz S, Fisher-Bowman J, Karlage A (2018). Adherence to essential birth practices and perinatal mortality in Uttar Pradesh, India. Int J Gynecol Obstet.

[26] IHI’s Collaborative Model for Achieving Breakthrough Improvement. [Internet]. Boston: Institute for Healthcare Improvement; 2003 [cited 2019 Sep 29]. (IHI Innovation Series). Available from: http://www.ihi.org/resources/Pages/IHIWhitePapers/TheBreakthroughSeriesIHIsCollaborativeModelforAchievingBreakthroughImprovement.aspx.

[27] The Breakthrough Series (2004). IHI’s Collaborative Model for Achieving Breakthrough Improvement. Diabetes Spectrum.

[28] UCSF IGHS. UCSF SPARQ PROJECT [Internet]. UCSF SPARQ PROJECT. 2017 [cited 2020 Apr 30]. Available from: https://globalhealthsciences.ucsf.edu/our-work/diseases-and-conditions/maternal-and-newborn-child-health/sparq-strengthening-people.

[29] Afulani PA, Diamond-Smith N, Phillips B, Singhal S, Sudhinaraset M (2018). Validation of the person-centered maternity care scale in India. Reprod Health.

[30] Phillips B, Kajal F, Montagu D, Kumar A, Kumar V (2018). Is bigger better? Assessment of self-reported and researcher-collected data on maternal health care quality among high-case-load facilities in Uttar Pradesh: a mixed-methods study. Lancet Global Health.

[31] Landrian A, Phillips BS, Singhal S, Mishra S, Kajal F, Sudhinaraset M (2020). Do you need to pay for quality care? Associations between bribes and out-of-pocket expenditures on quality of care during childbirth in India. Health Policy Plan.

