# Supplementary material for: A comparison of intensive vs. light-touch quality improvement interventions for maternal health in Uttar Pradesh, India
Source: BMC Health Serv Res. 2020 Dec 4;20:1121. doi: 10.1186/s12913-020-05960-6 (PMC7716449; doi:10.1186/s12913-020-05960-6)
Supplement: Supplementary file 1 — Additional file 1. [file 12913_2020_5960_MOESM1_ESM.pdf]

MH Phase 2 Prov. Endline Survey-QI facilities-Sept 2018

Field	Question	Answer
**Provider Survey Delivery**		
intro_b	Welcome to Provider Survey(Delivery doctors and nurses). We are carrying out a study to learn about the care provided to women during delivery and family planning services in this health facility. We have a few starting questions before we begin the survey.<br>नमस्कार । हम लोग यह अध्ययन, यह सीखने के लिए कर रहे है कि , इस स्वास्थ्य सुविधा मे परिवार नियोजन एवं प्रसव के लिए आई हुयी महिलाओं को किस प्रकार कि सुविधाए, देखभाल प्रदान की जाती है । हम लोग साक्षात्कार शुरू करने से पहले कुछ शुरुआती बाते जानना चाहेंगे ?	
enum_name *(required)*	Enumerator name	[redacted]
resp_id *(required)*	Enter Respondent ID	
resp_id2 *(required)*	Confirm Respondent ID<br>*Response constrained to: .= ${resp_id}*	
elig1_b	Do you plan to leave this facility before end 2019?<br>क्या आप यह स्वस्थ्य सुविधा 2019 के आखिरी से पहले छोडने की सोच रहे है ?	1 Yes / हाँ<br>0 No / नहीं
elig2_b *(required)*	Would you be willing to do the interview now in a private space at the facility?<br>क्या आप यह साक्षात्कार इसी स्वस्थ्य मे किसी अन्य (अलग/ एकांत) जगह पर देना चाहेंगे ?	1 Yes / हाँ<br>0 No / नहीं
**Provider Survey Delivery > Cover Page**		
consent_b	Informed Consent Form<br>Conducting survey of Providers and Facility Staff<br>(To be read to participant prior to the survey)<br><br>Study Title: Strengthening Person-Centered Accessibility, Respect, and Quality (SPARQ)<br><br>Introduction: Hello. My name is __________________. I work for Populations Services International (PSI) and we are studying the health care available for mothers and children in this area. We are carrying out this study in collaboration with researchers at the University of California, San Francisco.<br>Purpose of the study: We are carrying out a study to learn about the care provided to women during delivery and family planning services in this health facility.<br>Procedures: If you agree to take part in the study, we will ask you questions about your experiences as a provider of maternity and/or family planning services at the health facility. The interview will be conducted in a private place and will take approximately 15-30 minutes. We will follow-up with you to conduct one or more follow-up surveys.<br><br>Privacy and confidentiality: The information you provide during this survey will be kept confidential and used only for the specific purpose of this study. Your name and other information that could reveal your identity will be removed before the results of the study are made public or shared between people other than the main researchers working on the project. The information you tell us is strictly confidential and will not be shared with this facility as we are not affiliated with this facility.<br><br>Risks and benefits of participation: Before you decide whether you want to participate, it is important to listen to the following information carefully and discuss it with others if you wish. If you chose to answer these questions there will not be a direct benefit to you but you will help us to understand if and how to improve care provided to women delivering and receiving family planning services in the health facilities, which could benefit other expectant mothers in India and elsewhere. Please ask me if there is anything that is not clear or if you would like more information.<br><br>Withdrawal: Participation in this study is completely voluntary. Choosing not to take part will not disadvantage you in any way. It is up to you to decide whether to take part or not. If you decide to take part you are free to withdraw at any time and without giving a reason. You are also free to not answer any question that you do not wish to answer.<br><br>Questions and contacts: If you have any questions or concerns at a later time, you may contact the SPARQ Co-investigator, [redacted] If you have additional questions about your rights as a research subject, you can contact the UCSF Institutional Review Board at +001.415.476.1814.<br>Consent<br>If you decide to participate in this study, you will be asked to sign this consent form or make your thumbprint in front of a witness. A copy of this consent form will be provided to you, please indicate whether you agree to participate by signing below.<br>Would you like to participate? 0Yes 0No<br><br>STATEMENT OF CONSENT AND SIGNATURES<br>I have read this form or had it read to me. I have discussed the information with study staff. My questions have been answered. I understand that my decision whether or not to take part in the study is voluntary. I understand that if I decide	

	to join the study I may withdraw at any time. By signing this form I do not give up any rights that I have as a research participant. If you are uncomfortable writing and signing your name on this form, please feel free to make a mark of your choice to indicate you have understood the study and are willing to participate.<br><br>[redacted]		
consented_b *(required)*	Did the respondent consent to do the interview?<br>क्या सेवा प्रदाता ने साक्षात्कार के लिए अपनी सहमति प्रदान की है	1	Yes / हाँ
		0	No / नहीं
**Provider Survey Delivery > Cover Page > Identification**<br>*Group relevant when: ${consented_b} = 1*			
starttime_b *(required)*	Time interview begun		
prov_survey_date_b *(required)*	Date / तारीख<br>*Response constrained to: . <= today() and . >= today()*		
[redacted]	[redacted]		[redacted]
[redacted]	[redacted]		[redacted]
facility_type_b *(required)*	Facility type<br>स्वास्थ्य सुविधा का सुविधा प्रकार	1	Gov't Health Center / सरकारी स्वास्थ्य केंद्र
Level *(required)*	Level of care<br>स्वस्थ्य सुविधा का स्तर	1	First Referral Unit
		2	Community Health Center/First Referral Unit
		3	Community Health Center
		4	Primary Health Center
		5	Block Primary Health Center
**Provider Survey Delivery > Cover Page > Respondent**<br>*Group relevant when: ${consented_b} = 1*			
gender_b *(required)*	Gender<br>लिंग	1	Male / पुरुष
		2	Female / महिला
age_b *(required)*	How old are you?<br>उम्र<br>*Response constrained to: .>=18 and .<=80*		
religion_b *(required)*	What is your religion?<br>धर्म	1	Protestant / प्रोटेस्टेंट
		2	Catholic / कैथोलिक
		3	Muslim / मुस्लिम
		4	Hindu / हिन्दू
		6	other Christian
		5	Other (specify) / अन्य (स्पष्ट करे)
		7	none
Religion_Others *(required)*	Others<br>*Question relevant when: ${religion_b} =5*		
qualification_b *(required)*	What are your Training/Qualification(s)?<br>प्रशिक्षण / योग्यता	1	Less than certificate
		2	Certificate
		3	Diploma
		4	Degree
		5	Masters
		6	Specialty
qualification_bo *(required)*	Others Qualifications<br>*Question relevant when: not(${qualification_b} = '1')*		

specialty_b *(required)*	What is your specialization?<br>आप की विशेषग्यता किस पर है ?	
position_b *(required)*	What is your position in this facility?	1 Doctor / डॉक्टर<br>2 Clinical officer / क्लिनिकल अफ़सर<br>3 Nurse/Midwife / नर्स / मिडवाइफ<br>4 AuxiliaryNurseMidwife (ANM) / सहायक नर्समिडाइफ़ (एएनएम)<br>5 Other (specify) / अन्य (स्पष्ट करे)
Position_Others *(required)*	Others Specify<br>*Question relevant when: ${position_b} =5*	
hours_week_b *(required)*	How many hours do you work per week?<br>आप प्रति साप्ताह कितने घंटे काम करते है ?<br>*Response constrained to: .>=1 and .<=150*	
Provider Survey Delivery > Cover Page > Respondent > Duration		
years_position_b *(required)*	How long have you been at this facility: In YEARS?<br>इस स्वास्थ्य सेवा केन्द्र मे आपका कितने वर्षों का अनुभव है?<br>*Response constrained to: .>=0 and .<=60*	
months_position_b *(required)*	How long have you been at this facility: In MONTHS?<br>इस स्वास्थ्य सेवा केन्द्र मे आपका कितने माह का अनुभव है?<br>*Response constrained to: .>=0 and .<=11*	
Provider Survey Delivery > Cover Page > Years of experience<br>*Group relevant when: ${consented_b} = 1*		
years_position_b1 *(required)*	How long have you been in this position in total: IN YEARS?<br>इस स्वास्थ्य सुविधा मे आप का इस पद पर कितने वर्षों का अनुभव है?<br>*Response constrained to: .>=0 and .<=60*	
months_position_b1 *(required)*	How long have you been a in this position in total: IN MONTHS?<br>इस स्वास्थ्य सुविधा मे आप का इस पद पर कितने माह का अनुभव है?<br>*Response constrained to: .>=0 and .<=11*	
Provider Survey Delivery > Other<br>*Group relevant when: ${elig1_b} = 0 and ${consented_b} = 1*		
note_2_b	For the next set of questions, please tell me if each situation is acceptable. Choose either unacceptable in all instances, acceptable in certain instances or acceptable in all instances.<br>आगे दिए गये प्रश्नो के लिए कृपया बताए की इन परिस्थितियो के लिए निम्न स्वीकार्या है या नही ,सभी मामलो मे अस्वीकार्य ,कुछ मामलो मे स्वीकार्य ,सभी मामलो मे स्वीकार्य	
accept_wait_b *(required)*	Do you think it is unacceptable in all instances, acceptable in certain instances, or acceptable in all instances…<br>For delivery patients to wait for more than one hour before being seen?<br>क्या आपको लगता है की गर्भवती महिलाओ को देखे जाने से पहले एक घंटे से अधिक प्रतीक्षा करनी पड़ती है ?	1 Unacceptable in all instances / सभी मामलो मे अस्वीकार्य<br>2 Acceptable in certain instances / कुछ मामलो मे स्वीकार्य<br>3 Acceptable in all instances / सभी मामलो मे स्वीकार्य
accept_noinfo_b *(required)*	Do you think it is unacceptable in all instances, acceptable in certain instances, or acceptable in all instances….<br>For other people NOT involved in delivery patients' care to see their health information?<br>क्या आपको लगता है की प्रसूति के लिए आयी महिलाओ को उनके स्वस्थ्य देखभाल की जानकारी नही दी जाती है ?	1 Unacceptable in all instances / सभी मामलो मे अस्वीकार्य<br>2 Acceptable in certain instances / कुछ मामलो मे स्वीकार्य<br>3 Acceptable in all instances / सभी मामलो मे स्वीकार्य
accept_other_info_b *(required)*	Do you think it is unacceptable in all instances, acceptable in certain instances, or acceptable in all instances……<br><br>For other people NOT involved in delivery patients' care to see their health information?<br>प्रसूति के लिए आयी महिलाओ की स्वस्थ की जानकारी अन्य लोगो , अन्य सेवा प्रदाता को साझा की जाती है जो महिला की देखभाल मे शामिल नही है।	1 Unacceptable in all instances / सभी मामलो मे अस्वीकार्य<br>2 Acceptable in certain instances / कुछ मामलो मे स्वीकार्य<br>3 Acceptable in all instances / सभी मामलो मे स्वीकार्य
accept_provider_shout_b *(required)*	Do you think it is unacceptable in all instances, acceptable in certain instances, or acceptable in all instances……<br>For providers to shout at or scold delivery patients?<br>सेवा प्रदाता का मरीज पर चिल्लाना या डाटना ?	1 Unacceptable in all instances / सभी मामलो मे अस्वीकार्य<br>2 Acceptable in certain instances / कुछ मामलो मे स्वीकार्य<br>3 Acceptable in all instances / सभी मामलो मे स्वीकार्य
accept_provider_hit_b *(required)*	Do you think it is unacceptable in all instances, acceptable in certain instances, or acceptable in all instances………<br>For providers to hit delivery patients?<br>सेवा प्रदाता का मरीज को मारना ?	1 Unacceptable in all instances / सभी मामलो मे अस्वीकार्य<br>2 Acceptable in certain instances / कुछ मामलो मे स्वीकार्य

		3 Acceptable in all instances / सभी मामलो मे स्वीकार्य
accept_facility_dirty_b *(required)*	Do you think it is unacceptable in all instances, acceptable in certain instances, or acceptable in all instances……<br>For facilities to be dirty?<br>स्वस्थ सेवा केंद्र का गंदा होना ?	1 Unacceptable in all instances / सभी मामलो मे अस्वीकार्य<br>2 Acceptable in certain instances / कुछ मामलो मे स्वीकार्य<br>3 Acceptable in all instances / सभी मामलो मे स्वीकार्य
accept_diff_age_b *(required)*	Do you think it is unacceptable in all instances, acceptable in certain instances, or acceptable in all instances…..<br>For delivery patients to receive better care because of their age?<br>प्रसूति के लिए आयी महिलाओ उम्र के कारण बेहतर सेवा प्राप्त करना ?	1 Unacceptable in all instances / सभी मामलो मे अस्वीकार्य<br>2 Acceptable in certain instances / कुछ मामलो मे स्वीकार्य<br>3 Acceptable in all instances / सभी मामलो मे स्वीकार्य
accept_diff_married_b *(required)*	Do you think it is unacceptable in all instances, acceptable in certain instances, or acceptable in all instances…..<br>For delivery patients to receive better care because they are married?<br>प्रसूति के लिए आयी महिलाओ का बेहतर देखभाल प्राप्त करना क्योकि महिला विवाहित है ?	1 Unacceptable in all instances / सभी मामलो मे अस्वीकार्य<br>2 Acceptable in certain instances / कुछ मामलो मे स्वीकार्य<br>3 Acceptable in all instances / सभी मामलो मे स्वीकार्य
accept_diff_educated_b *(required)*	Do you think it is unacceptable in all instances, acceptable in certain instances, or acceptable in all instances……<br>For a patient to receive better care because she is educated?<br>प्रसूति के लिए आयी महिलाओ का बेहतर देखभाल प्राप्त करना क्योकि महिला शिक्षित है	1 Unacceptable in all instances / सभी मामलो मे अस्वीकार्य<br>2 Acceptable in certain instances / कुछ मामलो मे स्वीकार्य<br>3 Acceptable in all instances / सभी मामलो मे स्वीकार्य
accept_diff_wealthy_b *(required)*	Do you think it is unacceptable in all instances, acceptable in certain instances, or acceptable in all instances……<br>For delivery patients to receive better care because they are wealthy?<br>प्रसूति के लिए आयी महिलाओ का बेहतर देखभाल प्राप्त करना क्योकि महिला अमीर है	1 Unacceptable in all instances / सभी मामलो मे अस्वीकार्य<br>2 Acceptable in certain instances / कुछ मामलो मे स्वीकार्य<br>3 Acceptable in all instances / सभी मामलो मे स्वीकार्य
accept_bribe_b *(required)*	Do you think it is unacceptable in all instances, acceptable in certain instances, or acceptable in all instances…..<br>For any hospital staff, including doctors, nurses, cleaners, technicians to ask for money beyond the required amount ?<br>प्रसूति के लिए आयी महिलाओ से स्वस्थ्य सेवा प्रदाता का अलग से [अतिरिक्त] पैसे माँगना ?	1 Unacceptable in all instances / सभी मामलो मे अस्वीकार्य<br>2 Acceptable in certain instances / कुछ मामलो मे स्वीकार्य<br>3 Acceptable in all instances / सभी मामलो मे स्वीकार्य
work_b	Now I want to ask you two questions about your work.<br>अब मै आपसे आपके कार्य से संबन्धित दो प्रश्न पूछूगा	
job_satisfied_b *(required)*	How satisfied are you with your job?<br>आप अपनी नौकरी से कितने संतुष्ट है?	1 Very satisfied / संतुष्ट बहुत<br>2 Satisfied / संतुष्ट<br>3 Somewhat satisfied / कुछ हद तक संतुष्ट<br>4 Not satisfied at all / बिल्कुल भी संतुष्ट नही
recommend_friend_b *(required)*	Would you recommend this facility to your friends/family?<br>क्या आप अपने दोस्तो / परिवार के लोगो को इस स्वास्थ्य सेवा केंद्र का सुझाव देंगे?	1 Yes, definitely / हां, निश्चित रूप से<br>2 Yes, somewhat / हां, कुछ हद तक<br>3 No / नहीं
wait_b	Now I will ask you a few questions about the services provided to delivery patients at this facility. Please remember to be as honest as possible. Anything you say will be kept confidential and nothing you say will be associated with your name.<br><br>अब हम आपसे प्रसूति के लिए आयी महिलाओ को इस सुविधा मे मिल रही सुविधाओं के बारे मे जानना चाहेंगे । कृपया ईमानदारी से आप हमको सही उत्तर देजिएगा आप द्वारा दी गयी जानकारी पूरी तरह से गोपनीय रखी जाएगी और आपका नाम कही नहीं आएगा ।	
del_patient_wait_b *(required)*	When delivery patients first arrive at the health facility, how long do they typically have to wait before a doctor or nurse first examines them?<br>जब मरीज प्रसव के लिए स्वास्थ्य सेवा केंद्र पर पहुचता है तो डॉक्टर या नर्स के जाँच करने से पहले उसे कितनी देर तक इंतजार करना पड़ता है?	1 Do not wait at all<br>2 Less than 5 mins<br>3 5-15mins<br>4 16-30mins<br>5 31-45mins<br>6 46- to 60mins<br>7 61 min-1.5 hours

		8 More than 1.5 hrs to 2 hrs<br>9 More than 2 hrs to 3 hrs<br>10 More than 3 hrs to 4 hrs<br>11 More than 4 hours
pcc_time_b *(required)*	How do you feel about the amount of time delivery patients wait? Would you say it is very short, somewhat short, somewhat long, or very long?<br>प्रसव के लिए आए मरीज द्वारा इंतजार करने की अवधि के बारे मे आप क्या महसूस करते है? क्या आप उसे कहेगे बहुत कम, थोड़ी देर, कुछहद तक ज़्यादा देर, या बहुत ज़्यादा देर	1 Very short / बहुत कम देर<br>2 Somewhat short / थोड़ी देर<br>3 Somewhat long / कुछ हद तक ज़्यादा देर<br>4 Very long / बहुत ज़्यादा देर
provider_introduce_b *(required)*	During their time in the health facility do the doctors, nurses, or other health care providers introduce themselves to delivery patients when they first come to see them?<br>स्वास्थ्य सेवा केंद्र में अपने समय के दौरान क्या डॉक्टरों नर्सों या अन्य स्वास्थ्य देखभाल प्रदाताओ द्वारा खुद को, प्रसव के लिए आए मरीज़ो से परिचित करवाया जाता है?	1 No, none of them<br>2 Yes, a few of them<br>3 Yes, most of them<br>4 Yes, all of them
**Provider Survey Delivery > PCC**<br>*Group relevant when: ${consented_b} = 1*		
note_4_b	For the following questions, I want you to think about delivery services generally. I will ask you some questions about how delivery patients are treated at the health facility. Tell me if the following things happen all the time, most of the time, a few times, or it never happens. You can say a few times if it happens one or two times, and most of the time will be if it happens 3 or more times, but not always. For some questions I will ask specifically if something occurrs during labor, delivery, or after delivery. If I do not specify please answer based on your experiences during the entire time a delivery patients is in the facility labor until discharge.<br><br>(PROBE FOR ALL QUESTIONS: if respondent just responds, yes, ask them: Does this occur a few times, most of the time, or all the time)?<br><br>अब हम आपसे प्रसव के लिए आए मरीज़ो के साथ किए गये व्यवहार के बारे मे कुछ प्रशन पूछना चाहेंगे, मुझे ये बताएगा की क्या निम्नलिखित चीज़े हर समय होती है, कुछ समय होती है या कभी नही होती है, यदि एक या दो बार होती है तो आप बोल सकते है की कुछ समय,और यदि ३ या उससे अधिक बार होती है तो आप कह सकते है की ज़्यादातर है| अब हम प्रसव पीड़ा या प्रसव के दौरान या प्रसव के बाद के लिए मे विशेष रूप से कुछ प्रसन पूछना चाहूँगा| अगर मैं स्पष्ट नहीं करता हूं, तो पूरे समय के दौरान अपने अनुभवों के आधार पर उत्तर दें, प्रसूति के लिए आयी महिलाओ को स्वास्थ्य सुविधा से जब तक छुट्टी नहीं होती है।<br>(सभी प्रसनो की जाँच करें यदि उत्तर दाता केवल हाँ मे उत्तर देता है तो उनसे पूछे की क्या यह कुछ समय होता है, ज़्यादातर समय होता है या हमेशा होता है)?	
pcc_name_b *(required)*	Do the doctors, nurses, or other health care providers call delivery patients by their name?<br>क्या डॉक्टर या अन्य स्वास्थ्य सेवा प्रदाता प्रसूति के लिए आयी महिलाओ को उनके नाम से बुलाते है?	1 No, never / नही कभी नही<br>2 Yes, a few times / हाँ कभी कभी<br>3 Yes, most of the time / हाँ, ज़्यादातर समय<br>4 Yes, all the time / हाँ हर समय
pcc_respect_b *(required)*	Do the doctors, nurses, or other staff at the facility treat delivery patients with respect?<br>क्या आप महसूस करते है की स्वस्थ सेवा केंद्र पर डॉक्टर नर्स या अन्य कर्मचारी प्रसूति के लिए आयी महिलाओ के साथ सम्मानजनक व्यवहार करते है?	1 No, never / नही कभी नही<br>2 Yes, a few times / हाँ कभी कभी<br>3 Yes, most of the time / हाँ, ज़्यादातर समय<br>4 Yes, all the time / हाँ हर समय
pcc_friendly_b *(required)*	Do the doctors, nurses, and other staff at the facility treat delivery patients in a friendly manner?<br>क्या स्वस्थ सेवा केंद्र पर डॉक्टर नर्स या अन्य कर्मचारी प्रसूति के लिए आयी महिलाओ के साथ मित्रवत व्यवहार करते है?	1 No, never / नही कभी नही<br>2 Yes, a few times / हाँ कभी कभी<br>3 Yes, most of the time / हाँ, ज़्यादातर समय<br>4 Yes, all the time / हाँ हर समय
pcc_cared_b *(required)*	Do the doctors, nurses, and other staff at the facility show that they care about delivery patients?<br>क्या स्वस्थ सेवा केंद्र पर डॉक्टर नर्स या अन्य कर्मचारी ये दिखाते है की उन्हे प्रसूति के लिए आयी महिलाओ की चिंता है?	1 No, never / नही कभी नही<br>2 Yes, a few times / हाँ कभी कभी<br>3 Yes, most of the time / हाँ, ज़्यादातर समय<br>4 Yes, all the time / हाँ हर समय
pcc_privacy_aud_b *(required)*	When doctors and nurses are speaking with delivery patients, do you feel other people NOT involved in her care can hear what is being discussed?<br>क्या स्वस्थ सेवा केंद्र पर डॉक्टर नर्स प्रसव मरीज के साथ बात कर रहे होते है, तो क्या आपने ये महसूस किया की अन्य लोग जो की देखभाल मे शामिल नही है वो वहा हो रही चर्चा को सुन सकते है?	1 No, never / नही कभी नही<br>2 Yes, a few times / हाँ कभी कभी<br>3 Yes, most of the time / हाँ, ज़्यादातर समय<br>4 Yes, all the time / हाँ हर समय
pcc_privacy_vis_b *(required)*	During examinations in the labor room, are delivery patients covered up with a cloth or blanket or screened with a curtain so that they do not feel exposed?<br>प्रसव कक्ष मे परीक्षण के दौरान, क्या प्रसूति के लिए आयी महिलाओ को कपड़े या कंबल या पर्दे से ढाका जाता है जिससे की वा खुला हुआ ना महसूस करें?	1 No, never / नही कभी नही<br>2 Yes, a few times / हाँ कभी कभी<br>3 Yes, most of the time / हाँ, ज़्यादातर समय

		4	Yes, all the time / हाँ हर समय
pcc_info_confidential_b *(required)*	Do you feel like patient health information is kept confidential at this facility?<br>क्या आप महसूस करते है की मरीज की स्वास्थ्य सूचना इस स्वास्थ्य सेवा केंद्र पर गोपनीय रखी जाती है?	1	No, never / नही कभी नही
		2	Yes, a few times / हाँ कभी कभी
		3	Yes, most of the time / हाँ, ज़्यादातर समय
		4	Yes, all the time / हाँ हर समय
pcc_involvement_b *(required)*	Do you feel like the doctors, nurses or other staff at the facility involve delivery patients in decisions about their care?<br>क्या आप महसूस करते है की स्वस्थ सेवा केंद्र पर डॉक्टर नर्स या अन्य कर्मचारी मरीजों को उनके देखभाल के निर्णय मे शामिल करते है?	1	No, never / नही कभी नही
		2	Yes, a few times / हाँ कभी कभी
		3	Yes, most of the time / हाँ, ज़्यादातर समय
		4	Yes, all the time / हाँ हर समय
pcc_permission_b *(required)*	Do the doctors, nurses or other staff at the facility ask delivery patients for their permission/consent before doing procedures on them?<br>क्या आप महसूस करते है की स्वस्थ सेवा केंद्र पर डॉक्टर नर्स या अन्य करंचारियो द्वारा प्रसूति के लिए आयी महिलाओ उनका इलाज करने से पहले उनकी अनुमति / सहमति के लिए पूछते है ?	1	No, never / नही कभी नही
		2	Yes, a few times / हाँ कभी कभी
		3	Yes, most of the time / हाँ, ज़्यादातर समय
		4	Yes, all the time / हाँ हर समय
pcc_position_choice_b *(required)*	During the delivery, do doctors and nurses allow women to be in the position of her choice?<br>प्रसव के दौरान, क्या डॉक्टर और नर्स महिलाओं को अपनी पसंद की स्थिति में रहने की अनुमति देते हैं?	1	No, never / नही कभी नही
		2	Yes, a few times / हाँ कभी कभी
		3	Yes, most of the time / हाँ, ज़्यादातर समय
		4	Yes, all the time / हाँ हर समय
pcc_language_b *(required)*	Do the doctors, nurses or other staff at the facility speak to delivery patients in a language they can understand?<br>क्या स्वस्थ सेवा केंद्र पर डॉक्टर नर्स या अन्य करंचारियो द्वारा प्रसव मरीज से ऐसी भाषा मे बात की जाती है जो वो समझ सके?	1	No, never / नही कभी नही
		2	Yes, a few times / हाँ कभी कभी
		3	Yes, most of the time / हाँ, ज़्यादातर समय
		4	Yes, all the time / हाँ हर समय
pcc_explain_exam_b *(required)*	Do the doctors and nurses explain to delivery patients why they are doing examinations or procedures on them?<br>क्या डॉक्टरों नर्सों द्वारा महिलाओं को ये बताया जाता है की उन पर कोई परीक्षण या इलाज क्यो किया जा रहा है?	1	No, never / नही कभी नही
		2	Yes, a few times / हाँ कभी कभी
		3	Yes, most of the time / हाँ, ज़्यादातर समय
		4	Yes, all the time / हाँ हर समय
pcc_explain_meds_b *(required)*	Do the doctors and nurses explain to delivery patients why they are giving them any medicine?<br>क्या डॉक्टरों नर्सों द्वारा प्रसूति के लिए आयी महिलाओ को ये बताया जाता है की उन्हे कोई दवा किस तकलीफ के कारण दी जा रही है?	1	No, never / नही कभी नही
		2	Yes, a few times / हाँ कभी कभी
		3	Yes, most of the time / हाँ, ज़्यादातर समय
		4	Yes, all the time / हाँ हर समय
pcc_feeling_b *(required)*	Do the doctors and nurses at the facility talk to delivery patients about how they are feeling?<br>क्या स्वस्थ सेवा केंद्र पर डॉक्टरों नर्सों द्वारा प्रसव मरीजों से बात करके ये पूछा जाता है की वो कैसा महसूस कर रहे है?	1	No, never / नही कभी नही
		2	Yes, a few times / हाँ कभी कभी
		3	Yes, most of the time / हाँ, ज़्यादातर समय
		4	Yes, all the time / हाँ हर समय
pcc_anxieties_b *(required)*	Do the doctors, nurses or other staff at the facility try to understand the anxieties and fears of delivery patients?<br>क्या स्वस्थ सेवा केंद्र पर डॉक्टरों नर्सों या अन्य कर्मचारीओ द्वारा प्रसव मरीजों की चिन्ताओ और डर को समझने की कोशिश की जाती है?	1	No, never / नही कभी नही
		2	Yes, a few times / हाँ कभी कभी
		3	Yes, most of the time / हाँ, ज़्यादातर समय
		4	Yes, all the time / हाँ हर समय
pcc_questions_b *(required)*	Do you feel delivery patients can ask the doctors, nurses or other staff at the facility any questions they have?<br>क्या आपको लगता है की प्रसूति के लिए आयी महिलाओ स्वस्थ सेवा केंद्र पर डॉक्टरों नर्सों या अन्य कर्मचारीओ से कोई भी प्रशन पूछ सकते है जो भी उनको पूछना हो?	1	No, never / नही कभी नही
		2	Yes, a few times / हाँ कभी कभी
		3	Yes, most of the time / हाँ, ज़्यादातर समय
		4	Yes, all the time / हाँ हर समय
pcc_support_labor_b *(required)*	Are delivery patients allowed to have someone they want (outside of staff at the facility, such as family or friends) stay with them during labor?<br>क्या प्रसूति के लिए आयी महिलाओ को यह अनुमति है की वह प्रसव पीड़ा के दौरान अपने साथ किसी व्यक्ति को (जो भी उस स्वस्थ सेवा केंद्र के बाहर का हो जैसे की परिवार या दोस्त) को ठहरा सकता है?	1	No, never / नही कभी नही
		2	Yes, a few times / हाँ कभी कभी
		3	Yes, most of the time / हाँ, ज़्यादातर समय
		4	Yes, all the time / हाँ हर समय
pcc_support_del_b *(required)*	Are delivery patients allowed to have someone they want stay with them during delivery?<br>क्या प्रसव के दौरान किसी व्यक्ति को जिसे प्रसूता वह साथ रखना चाहती हो महिला के साथ रहने की इजाज़त है?	1	No, never / नही कभी नही
		2	Yes, a few times / हाँ कभी कभी
		3	Yes, most of the time / हाँ, ज़्यादातर समय

		4	Yes, all the time / हाँ हर समय
pcc_attention_help_b *(required)*	When delivery patients need help, do you feel the doctors, nurses or other staff at the facility pay attention?<br>जब प्रसव मरीज़ो को मदद की ज़रूरत होती है क्या आपको लगता है की स्वस्थ सेवा केंद्र पर डॉक्टर नर्स या अन्य करंचारियो द्वारा उन पर ध्यान दिया जाता है?	1	No, never / नही कभी नही
		2	Yes, a few times / हाँ कभी कभी
		3	Yes, most of the time / हाँ, ज़्यादातर समय
		4	Yes, all the time / हाँ हर समय
pcc_ask_pain_b *(required)*	Do the doctors and nurses ask how much pain delivery patients are in?<br>क्या डॉक्टरों या नर्सों द्वारा प्रसव मरीज़ो से पूछा जाता है की वो कितने दर्द मे है?	1	No, never / नही कभी नही
		2	Yes, a few times / हाँ कभी कभी
		3	Yes, most of the time / हाँ, ज़्यादातर समय
		4	Yes, all the time / हाँ हर समय
pcc_control_pain_b *(required)*	Do you feel the doctors or nurses do everything they can to help control delivery patients' pain?<br>क्या आप महसूस करते हैं कि डॉक्टर या नर्सें प्रसव के रोगियों के दर्द को नियंत्रित करने में मदद करने के लिए वे सब कुछ करते है जो सब कुछ वो कर सकते है?	1	No, never / नही कभी नही
		2	Yes, a few times / हाँ कभी कभी
		3	Yes, most of the time / हाँ, ज़्यादातर समय
		4	Yes, all the time / हाँ हर समय
pcc_attention_stay_b *(required)*	Do you feel the doctors and nurses pay attention to delivery patients during their stay in the facility?<br>क्या आपको लगता है की स्वास्थ्य सेवा केंद्र पर डॉक्टरों या नर्सों द्वारा प्रसव मरीज के वहाँ रहने के दौरान उन पर ध्यान दिया जाता है?	1	No, never / नही कभी नही
		2	Yes, a few times / हाँ कभी कभी
		3	Yes, most of the time / हाँ, ज़्यादातर समय
		4	Yes, all the time / हाँ हर समय
pcc_eat_drink_b *(required)*	Are delivery patients allowed to eat or drink when they are hungry/thirsty?<br>क्या प्रसूता महिलाओ को भूखे प्यासे होने पर खाने या पीने की इजाज़त होती है?	1	No, never / नही कभी नही
		2	Yes, a few times / हाँ कभी कभी
		3	Yes, most of the time / हाँ, ज़्यादातर समय
		4	Yes, all the time / हाँ हर समय
pcc_abuse_verbal_b *(required)*	Do you feel the doctors, nurses, or other health providers shout, scold, insult, threaten, or talk to delivery patients rudely?<br>क्या आपको लगता है की डॉक्टर या नर्से या अन्य स्वास्थ्य प्रदाता प्रसव मरीज़ो पर चिल्लाते है डाटते है, अपमान करते है, धमकाते है या बुरी तरह बात करते है?	1	No, never / नही कभी नही
		2	Yes, a few times / हाँ कभी कभी
		3	Yes, most of the time / हाँ, ज़्यादातर समय
		4	Yes, all the time / हाँ हर समय
pcc_abuse_physical_b *(required)*	Do you feel like delivery patients are treated roughly like pushed, beaten, slapped, pinched, physically restrained, or gagged?<br>क्या आपको लगता है की प्रसव मरीज़ो के साथ बुरा व्यवहार जैसे की धक्का देना, थप्पड़ मारना, नोचना, शारीरिक रूप से जकड़ना या बोलने नही दिया जाता?	1	No, never / नही कभी नही
		2	Yes, a few times / हाँ कभी कभी
		3	Yes, most of the time / हाँ, ज़्यादातर समय
		4	Yes, all the time / हाँ हर समय
pcc_force_stay_b *(required)*	Do you feel like delivery patients are forced to stay at the health facility against their will because they cannot pay their bill?<br>क्या आपको लगता है की प्रसव मरीज़ो को उनकी इच्छा के विपरीत स्वास्थ्य सेवा केंद्र पर रुकने के लिए मजबूर किया जाता है] क्योकि वो स्वस्थ सेवा केंद्र के बिल का भुगतान नही कर सकते?	1	No, never / नही कभी नही
		2	Yes, a few times / हाँ कभी कभी
		3	Yes, most of the time / हाँ, ज़्यादातर समय
		4	Yes, all the time / हाँ हर समय
pcc_bribe_b *(required)*	During their time at the facility, do any staff at the facility ask delivery patients or their family for money outside of official payment?<br>स्वास्थ्य सेवा केंद्र पर उनके समय के दौरान क्या वहाँ का कोई कर्मचारी प्रसूता से या उनके परिवार वालो से अलग से {अतिरिक्त} पैसे की मांग करता है?	1	No, never / नही कभी नही
		2	Yes, a few times / हाँ कभी कभी
		3	Yes, most of the time / हाँ, ज़्यादातर समय
		4	Yes, all the time / हाँ हर समय
pcc_enough_staff_b *(required)*	Do you think there is enough health staff in the facility to care for delivery patients?<br>क्या आपको लगता है कि स्वास्थ्य सेवा केंद्र पर प्रसव मरीजों की देखभाल के लिए पर्याप्त स्वास्थ्य कर्मचारी हैं?	1	No, never / नही कभी नही
		2	Yes, a few times / हाँ कभी कभी
		3	Yes, most of the time / हाँ, ज़्यादातर समय
		4	Yes, all the time / हाँ हर समय
pcc_bestcare_b *(required)*	Do you feel the doctors, nurses or other staff at the facility take the best care of delivery patients?<br>क्या आपको लगता है कि स्वास्थ्य सेवा केंद्र पर डॉक्टरों नर्से या अन्य कर्मचारी प्रसुता महिलाओं की अच्छी देखभाल करते है?	1	No, never / नही कभी नही
		2	Yes, a few times / हाँ कभी कभी
		3	Yes, most of the time / हाँ, ज़्यादातर समय
		4	Yes, all the time / हाँ हर समय
pcc_trust_provider_b *(required)*	Do you feel the doctors, nurses or other staff at the facility are completely trusted with regards to their delivery care?<br>क्या आपको लगता है की स्वास्थ्य सेवा केंद्र पर डॉक्टरों या नर्सों या अन्य कर्मचारीओ पर उसकी प्रसव देखभाल के संबंध मे भरोसा किया जा सकता है?	1	No, never / नही कभी नही
		2	Yes, a few times / हाँ कभी कभी
		3	Yes, most of the time / हाँ, ज़्यादातर समय

		4 Yes, all the time / हाँ हर समय
pcc_crowded_del_b *(required)*	Thinking about the labor and postnatal wards, do you feel the health facility is crowded?<br>प्रसव वॉर्ड और प्रसवोतर वॉर्ड के बारे मे सोच कर बताए की क्या आपको लगता है की स्वस्थ सेवा केंद्र पर भीड़ होती है?	1 No, never / नही कभी नही<br>2 Yes, a few times / हाँ कभी कभी<br>3 Yes, most of the time / हाँ, ज़्यादातर समय<br>4 Yes, all the time / हाँ हर समय
pcc_clean_b *(required)*	Thinking about the wards, washrooms and the general environment of the health facility, would you say the facility is very clean, clean, dirty, or very dirty?<br>स्वास्थ्य सेवा केंद्र के वॉर्ड, वॉशरूम और सामान्य वातावरण के बारे मे सोच कर बताए की क्या आप स्वस्थ सेवा केंद्र को साफ, साफ, बहुत साफ, गंदा या बहुत गंदा कहँगे?	1 Very clean / बहुत साफ<br>2 Clean / साफ<br>3 Dirty / गंदा<br>4 Very dirty / बहुत गंदा
pcc_water_b *(required)*	Is there water in the facility?<br>क्या स्वास्थ्य सुविधा में पानी है?	1 No, never / नही कभी नही<br>2 Yes, a few times / हाँ कभी कभी<br>3 Yes, most of the time / हाँ, ज़्यादातर समय<br>4 Yes, all the time / हाँ हर समय
pcc_electricity_b *(required)*	Is there electricity in the facility?<br>क्या स्वास्थ्य सेवा केंद्र मे बिजली है?	1 No, never / नही कभी नही<br>2 Yes, a few times / हाँ कभी कभी<br>3 Yes, most of the time / हाँ, ज़्यादातर समय<br>4 Yes, all the time / हाँ हर समय
pcc_safe_b *(required)*	In general, do delivery patients feel safe in the health facility?<br>सामान्य तौर पर क्या प्रसव मरीज स्वास्थ्य सुविधा केंद्र मे सुरक्षित महसूस करते हैं?	1 No, never / नही कभी नही<br>2 Yes, a few times / हाँ कभी कभी<br>3 Yes, most of the time / हाँ, ज़्यादातर समय<br>4 Yes, all the time / हाँ हर समय
endnote_b	"This is the end of the interview. Thank you for your time."<br>अब हमारा साक्षात्कार समाप्त होता। आप का समय देने के लिए बहोत बहोत धन्यवाद।	
comment_b *(required)*	Any comments?	
status_b *(required)*	To Enumerator: What is the status of the survey?<br>सर्वे अब पूरा हुआ	0 Incomplete<br>1 Complete<br>3 Ineligible(SAVE even if ineligible)<br>4 Refusal
