# Supplementary material for: A comparison of intensive vs. light-touch quality improvement interventions for maternal health in Uttar Pradesh, India
Source: BMC Health Serv Res. 2020 Dec 4;20:1121. doi: 10.1186/s12913-020-05960-6 (PMC7716449; doi:10.1186/s12913-020-05960-6)
Supplement: Supplementary file 4 — Additional file 4. [file 12913_2020_5960_MOESM4_ESM.pdf]

MH Phase 2 Patient Endline Survey

Field	Question	Answer
Intro	Welcome to the SPARQ Delivery Survey. We are carrying out a study to learn about the care that you have received when you delivered your last child in the health facility. We have a few starting questions before we begin the survey.<br><br>नमस्कार। SPARQ डिलिवरी सर्वेक्षण में आपका स्वागत है| हम स्वास्थ्य सुविधा में अपने आखिरी बच्चे को देने के दौरान आपके द्वारा प्राप्त की गई देखभाल के बारे में जानने के लिए एक अध्ययन कर रहे हैं। सर्वेक्षण शुरू करने से पहले हमारे पास कुछ शुरुआती प्रश्न हैं		
wel_conf	Welcome to the SPARQ Delivery Survey. We are carrying out a study to learn about the care that you have received when you delivered your last child in the health facility. We have a few starting questions before we begin the survey.<br><br>नमस्कार। SPARQ डिलिवरी सर्वेक्षण में आपका स्वागत है| हम स्वास्थ्य सुविधा में अपने आखिरी बच्चे को देने के दौरान आपके द्वारा प्राप्त की गई देखभाल के बारे में जानने के लिए एक अध्ययन कर रहे हैं। सर्वेक्षण शुरू करने से पहले हमारे पास कुछ शुरुआती प्रश्न हैं | *GPS coordinates can only be collected when outside.*	
Screen		
date *(required)*	1. Date (तिथि)<br>*Response constrained to: . <= today() and . >= today()*	
scree_1 *(required)*	2. Are you 18 to 49 years old<br><br>क्या आप 18 से 49 साल उमर के हैं		1 Yes (हाँ)<br>0 No (नहीं)
Scree_2 *(required)*	3. Have you delivered your baby in this facility within the past 7 days?<br><br>क्या आपने पिछले 7 दिनों में इस स्वाश्थ्य केंद्र में बच्चे को जन्म दिया है?<br>*Question relevant when: selected(${scree_1} , '1')*	1 Yes (हाँ)<br>0 No (नहीं)
witness_consent *(required)*	Witnessed Informed Consent of the participant (Delivery Services)<br><br>Informed Consent Form<br>Conducting survey of Recently Delivered Women<br>(To be read to participant prior to the survey)<br><br>Study Title: Strengthening Person-Centered Accessibility, Respect, and Quality (SPARQ)<br><br>Introduction: Hello. My name is ___________________. I work for Population Services International (PSI) and we are studying the health care available for mothers and children in this area. We are carrying out this study in collaboration with researchers at the University of California, San Francisco.<br>Purpose of the study: We are carrying out a study to learn about the care that you have received when you delivered your last child in the health facility.<br>Procedures: If you agree to take part in the study, we will ask you questions about your delivery experiences, and the nature of care you have received when you delivered your last baby at the health facility. The interview will be conducted in a private place and will take approximately 1 hour.<br><br>Privacy and confidentiality: The information you provide during this survey will be kept confidential and used only for the specific purpose of this study. Your name or the location of your house and other information that could reveal your identity will be removed before the results of the study are made public or shared between people other than the main researchers working on the project. Your data will be transferred to computers protected by passwords. We will not speak about anything that you personally have said unless you indicate that there is a real risk to your own or your baby's health. The information you tell us is strictly confidential and will not be shared with this facility as we are not affiliated with this facility. If during the interview, you are not feeling well, I will immediately help you seek care at this facility.<br>Risks and benefits of participation: Before you decide whether you want to participate, it is important to listen to the following information carefully and discuss it with others if you wish. If you chose to answer these questions there will not be a direct benefit to you but you will help us to understand if and how to improve care provided to women delivering in the health facilities, which could benefit other expectant mothers in India and elsewhere. Please ask me if there is anything that is not clear or if you would like more information.<br><br>Withdrawal: Participation in this study is completely voluntary. Choosing not to take part will not disadvantage you in any way. It is up to you to decide whether to take part or not. If you decide to take part you are free to withdraw at any time and without giving a reason. You are also free to not answer any question that you do not wish to answer.<br><br>Questions and contacts: If you have any questions or concerns at a later time, you may contact the SPARQ Co-investigator, [redacted]. If you have additional questions about your rights as a	1 Yes (हाँ)<br>0 No (नहीं)

research subject, you can contact the UCSF Institutional Review Board at +001.415.476.1814.

Consent

If you decide to participate in this study, you will be asked to sign this consent form or make your thumbprint in front of a witness. A copy of this consent form will be provided to you, please indicate whether you agree to participate by signing below.

Would you like to participate? 0Yes 0No

STATEMENT OF CONSENT AND SIGNATURES

I have read this form or had it read to me. I have discussed the information with study staff. My questions have been answered. I understand that my decision whether or not to take part in the study is voluntary. I understand that if I decide to join the study I may withdraw at any time. By signing this form I do not give up any rights that I have as a research participant. If you are uncomfortable writing and signing your name on this form, please feel free to make a mark of your choice to indicate you have understood the study and are willing to participate.

___________________ ________________________ _____________

Participant name (print) Participant signature/thumbprint Date

___________________ ________________________ ____________

Study staff conducting Study staff signature Date
consent discussion (print)

___________________ ________________________ _____________

Witness name, if thumbprint Witness signature Date
given (print)

[redacted]

सूचित सहमति प्रपत्र
हाल ही में वितरित महिलाओं के सर्वेक्षण का आयोजन
(सर्वेक्षण से पहले प्रतिभागी को पढ़ा जाना है)

अध्ययन शीर्षक: व्यक्ति-केंद्रित अभिगम्यता, सम्मान और गुणवत्ता को मजबूत बनाना (एसपीएआरक्यू)

परिचय: हैलो। मेरा नाम है ___________________। मैं जनसंख्या सेवा अंतर्राष्ट्रीय (पीएसआई) के लिए काम करती हूं और हम इस क्षेत्र में माताओं और बच्चों के लिए उपलब्ध स्वास्थ्य देखभाल का अध्ययन कर रहे हैं। हम इस अध्ययन को कैलिफोर्निया विश्वविद्यालय, सैन फ्रांसिस्को में शोधकर्ताओं के सहयोग से कर रहे हैं।

अध्ययन का उद्देश्य: जब आप अपने अंतिम बच्चे को स्वास्थ्य केन्द्र में जन्म देती हैं तो आपको प्राप्त होने वाली देखभाल के बारे में जानने के लिए हम एक अध्ययन कर रहे हैं।

प्रक्रियाएं: यदि आप अध्ययन में भाग लेने के लिए सहमत हैं, तो हम आपको आपके वितरण अनुभवों के बारे में प्रश्न पूछेंगे, और जब आपने स्वास्थ्य केंद्र पर अपना अंतिम बच्चा जन्म दिया था तो आपको जो देखभाल मिली थी। साक्षात्कार एक निजी स्थान पर आयोजित किया जाएगा और लगभग 1 घंटा लग जाएगा।

गोपनीयता: इस सर्वेक्षण के दौरान आपके द्वारा प्रदान की जाने वाली जानकारी को गोपनीय रखा जाएगा और केवल इस अध्ययन के विशिष्ट उद्देश्य के लिए उपयोग किया जाएगा। आपका नाम या आपके घर का स्थान और अन्य जानकारी जो आपकी पहचान प्रकट कर सकती है, अध्ययन के नतीजे सार्वजनिक परियोजनाओं के अलावा अन्य लोगों के बीच साझा किए जाने से पहले हटा दिए जाएंगे। आपका डेटा पासवर्ड द्वारा संरक्षित कंप्यूटरों में स्थानांतरित कर दिया जाएगा। हम किसी भी चीज के बारे में बात नहीं करेंगे जिसे आपने व्यक्तिगत रूप से कहा है जबतक कि आप इंगित न करें कि यह आपके और आपके बच्चे के स्वास्थ्य के लिए वास्तविक जोखिम है। जो जानकारी आप हमें बताते हैं वह पूर्ण तया गोपनीय है और इसे स्वास्थ्य केंद्र के साथ साझा नहीं की जाएगी क्योंकि हम इस स्वास्थ्य केंद्र से सम्बंधित नहीं हैं। यदि साक्षात्कार के दौरान, आप अच्छी तरह से महसूस नहीं कर रही हैं, तो मैं तुरंत इस स्वास्थ्य केंद्र पर आपकी देख भाल के लिए ध्यान देने में आपकी सहायता करुँगी |

जोखिम और भागीदारी के लाभ: इससे पहले कि आप यह तय करने से पहले कि आप भाग लेना चाहते हैं, निम्नलिखित जानकारी को ध्यान से सुनना महत्वपूर्ण है और यदि आप चाहें तो दूसरों के साथ चर्चा करना महत्वपूर्ण है। यदि आपने इन सवालों का जवाब देना चुना है तो आपके लिए

कोई सीधा लाभ नहीं होगा, लेकिन आप यह समझने में हमारी सहायता करेंगे कि स्वास्थ्य सुविधाओं में महिलाओं को प्रदान की जाने वाली देखभाल में सुधार कैसे किया जाए, जिससे भारत और अन्य जगहों पर अन्य उम्मीदवार माताओं को लाभ हो सके। कृपया मुझसे पूछें कि क्या ऐसी कोई बात है जो स्पष्ट नहीं है या यदि आप अधिक जानकारी चाहती हैं।

निकासी: इस अध्ययन में भागीदारी पूरी तरह से स्वतंत्र है। यदि आप इसमें भाग लेते हैं तो आप को किसी भी प्रकार का नुकशान नही होगा। यह तय करने के लिए यह आप पर निर्भर है कि भाग लेना है या नहीं। यदि आप भाग लेने का फैसला करती हैं तो आप किसी भी समय और बिना किसी भी कारण के आपना निर्णय वापस लेने के लिए स्वतंत्र हैं। आप किसी भी प्रश्न का उत्तर देने के लिए स्वतंत्र हैं जिसका आप जवाब देना नहीं चाहती हैं।

प्रश्न और संपर्क: यदि आपके पास बाद में कोई प्रश्न या चिंता है, तो आप [redacted] पर संपर्क कर सकते हैं। यदि आपके पास शोध विषय के रूप में आपके अधिकारों के बारे में अतिरिक्त प्रश्न हैं, तो आप +001.415.476.1814 पर यूसीएसएफ इंस्टीट्यूशनल रिव्यू बोर्ड से संपर्क कर सकते हैं।

सहमति
यदि आप इस अध्ययन में भाग लेने का निर्णय लेते हैं, तो आपको इस सहमति फॉर्म पर हस्ताक्षर करने या गवाह के सामने अपना अंगूठा बनाने के लिए कहा जाएगा। इस सहमति फॉर्म की एक प्रति आपको प्रदान की जाएगी, कृपया संकेत दें कि आप नीचे हस्ताक्षर करके भाग लेने के लिए सहमत हैं या नहीं।

क्या आप भागीदारी करना चाहेंगे? 0Yes 0No

सहमति और हस्ताक्षर का विवरण
मैंने इस फॉर्म को पढ़ा है। मैंने अध्ययन कर्मचारियों के साथ जानकारी पर चर्चा की है। मेरे सवालों का जवाब दिया गया है। मैं समझती हूं कि मेरा निर्णय अध्ययन में भाग लेना है या नहीं, स्वैच्छिक है। मैं समझती हूं कि अगर मैं अध्ययन में शामिल होने का फैसला करती हूं तो मैं किसी भी समय आपना निर्णय बदल सकती हूँ। इस फॉर्म पर हस्ताक्षर करके मैं शोध प्रतिभागी के रूप में मेरे पास कोई अधिकार नहीं छोड़ती हूं। यदि आप इस फॉर्म पर अपना नाम लिखने और हस्ताक्षर करने में असहज हैं, तो कृपया यह समझने के लिए कि आप अध्ययन समझ चुकी हैं और भाग लेने के इच्छुक हैं, अपनी पसंद का एक निशान बनाने के लिए स्वतंत्र महसूस करें।

Participant Name (print) Participant signature/thumb print Date

_________________ ______________________ ___________

Study staff conducting Study staff signature Date
Consent discussion (print)

_________________ ______________________ ___________

Witness name, if thumbprint Witness signature Date
Given (print)

Question relevant when: selected(${Scree_2} , '1')

**IDENTIFICATION (पहचान)** *Group relevant when: selected(${witness_consent} , '1')*		
Inter_name *(required)*	4. Interviewer's name	[redacted]
inter_num *(required)*	5. Interviewer's number (साक्षात्कारकर्ता का संख्या)	
resp_num *(required)*	6.1. Respondent's number (प्रतिवादी की संख्या)<br>*Response constrained to: .>=100 and .<=999*	
confir_respnumber *(required)*	6.2. Respondent's Number Confirmation<br><br>उत्तरदायित्व की संख्या पुष्टिकरण<br>*Response constrained to: .= ${resp_num}*	
[redacted]	[redacted]	[redacted]
[redacted]	[redacted]	
[redacted]	[redacted]	
[redacted]	[redacted]	[redacted]

[redacted] information

Group relevant when: *selected(${witness_consent} , '1')*

mother_age *(required)*	Now we will start with socio-demographic information. (अब हम सामाजिक-जनसांख्यिकीय जानकारी से शुरू करेंगे।)<br><br>1. How old were you at your last birthday?<br><br>(पिछले जन्मदिन पे आप कितने साल की हुई ?)<br>*Response constrained to: .>=18 and .<=49*	
maritalstatus *(required)*	2. What is your current marital status?<br><br>(क्या आप शादी शुदा है?)	1 Unmarried (अविवाहित)<br>2 Cohabiting/Partnered (साथी के साथ रहना)<br>3 Currently married (शादी शुदा)<br>4 Widowed (विधवा)<br>5 Divorced (तलाकशुदा)
literate *(required)*	3. Can you read/write?<br><br>क्या आप पढ़/लिख सकती हैं ?	1 Yes, and attended school (हाँ, और स्कूल में सीखा)<br>2 Yes, but did not attend schoool (हाँ, लेकिन स्कूल में नहीं गए)<br>0 No (नहीं)
edu_class *(required)*	4. What is the highest grade/class that you completed at school?<br><br>आपने किस कक्षा तक पढाई की है?	0 Did not go to school (स्कूल नहीं गए)<br>1 Primary (प्राथमिक कक्षा तक)<br>2 Till eighth standard/Post-primary (आठवी तक)<br>3 Secondary (माध्यमिक कक्षा तक)<br>4 College (कॉलेज (मध्य स्तर) तक)<br>5 University (विश्वविद्यालय तक)
occupation_a *(required)*	5 What is your current occupation?<br><br>आप क्या काम करती हैं?	1 Unemployed/Homemaker (बेरोजगार / गृहिणी)<br>2 Cultivator (कृषि)<br>3 Agricultural Labor (कृषि श्रम)<br>4 Casual Labor (आकस्मिक)<br>5 Salaried Worker (वेतनभोगी कर्मचारी)<br>6 Self-employed in petty trade (छोटे व्यापार में स्व-नियोजित)<br>7 Self-employed small scale industry (स्व-नियोजित लघु उद्योग)<br>8 Others (अन्य)
occupation_specify *(required)*	6. What is your current occupation? Other (Specify)<br><br>आप क्या काम करती हैं ? अन्य (स्पष्ट करे)<br>*Question relevant when: selected(${occupation_a} , '8')*	
hus_work *(required)*	7. What is your husband's occupation? Do you know?<br><br>आपके पति क्या काम करते है? आपको पता है ?<br>*Question relevant when: selected(${maritalstatus} , '3')*	99 Yes I know (पता है)<br>88 Don't Know (पता नहीं)
hus_occupation *(required)*	8. What is your husband's occupation?<br><br>आपके पति क्या काम करते है?<br>*Question relevant when: selected(${hus_work} , '99')*	1 Cultivator (कृषक)<br>2 Agricultural Labor (कृषि श्रम)<br>3 Casual Labor (आकस्मिक श्रम)<br>4 Salaried Worker (वेतनभोगी कर्मचारी)<br>5 Self-employed in petty trade (स्वनियोजित खुदरा व्यापार)<br>6 Self-employed in small scale industry (स्वनियोजित लघु उद्योग)<br>7 Unemployed (बेरोज़गार)<br>8 Others (अन्य)
hus_wrk_other *(required)*	9.What is your husband's occupation? Other (Specify)<br><br>आपके पति क्या काम करते है? अन्य (स्पष्ट करे)<br>*Question relevant when: selected(${maritalstatus} , '3') and selected(${hus_occupation} , '8')*<br>*Response constrained to: ((string-length(.)>=2 and string-length(.)<=15) and not(regex(.,'^(.*)\d(.*)$')) and*	

	not(regex(.,'^(.)\s(.*)$')) and not(regex(.,'^(.*)[\p{Punct}](.*)$')))*	
hh_income *(required)*	10. What's your total household income? (Monthly)<br><br>आपकी कुल घरेलू आय क्या है? (महीने के)	
religion *(required)*	11 What is your religion?<br><br>आपका धर्म क्या है?	1 Hindu (हिन्दू)<br>2 Muslim (मुस्लिम)<br>3 Christian (ईसाई)<br>4 Other (अन्य)
religion_other *(required)*	12. What is your religion? - Other<br><br>आपका धर्म क्या है? - अन्य<br>*Question relevant when: selected(${religion} , '4')*<br>*Response constrained to: ((string-length(.)>=2 and string-length(.)<=15) and not(regex(.,'^(.*)\d(.*)$')) and not(regex(.,'^(.*)\s(.*)$')) and not(regex(.,'^(.*)[\p{Punct}](.*)$')))*	
caste *(required)*	13.1 To what tribe/caste do you belong?<br><br>आप किस जाती या जन - जाती में शामिल हैं?	1 Scheduled Caste (अनुसूचित जाति)<br>2 Scheduled Tribes (अनुसूचित जनजाति)<br>3 General (सामान्य)<br>4 Other Backward Class (अन्य पिछड़ा वर्ग)<br>9 Others (specify)
caste_other	13.2 To what tribe/caste do you belong? Other (Specify)<br><br>आप किस जाती या जन - जाती में शामिल हैं? अन्य (स्पष्ट करें)<br>*Question relevant when: selected(${caste} , '9')*<br>*Response constrained to: ((string-length(.)>=2 and string-length(.)<=15) and not(regex(.,'^(.*)\d(.*)$')) and not(regex(.,'^(.*)\s(.*)$')) and not(regex(.,'^(.*)[\p{Punct}](.*)$')))*	
"Pregnancy and Child Birth History- All pregnancies"<br><br>(गर्भावस्था और प्रसव इतिहास - सभी गर्भावस्थाएं)<br>*Group relevant when: selected(${witness_consent} , '1')*		
no_delivery *(required)*	14. How many many times have you given birth in total?<br><br>आपने कितनी बार बच्चों को जन्म दिया है? (बार)<br>*Response constrained to: .>=1 and .<=20*	
child_death *(required)*	15. Have you ever given birth to a baby that was born alive but later died?<br><br>(क्या आपने कभी बच्चे को जन्म दिया है, जिसकी बाद में मृत्यु हो गयी हो?)	1 Yes (हाँ)<br>0 No (नहीं)
child_death_num *(required)*	16. How many babies have you had that later died?<br><br>ऐसा कितनी बार हुआ है? (बच्चा)<br>*Question relevant when: selected(${child_death} , '1')*<br>*Response constrained to: .>=1 and .<=20*	
babyborn *(required)*	Which day was your baby born	
Pregnancy and Childbirth History – Most recent pregnancy <br>(गर्भावस्था और प्रसव के इतिहास - हाल ही में गर्भावस्था)<br>*Group relevant when: selected(${witness_consent} , '1')*		
recentpreg_anc *(required)*	Now I am going to ask you questions about your most recent pregnancy<br><br>17. During your most recent pregnancy, did you attend any antenatal care visits?<br><br>अब मैं आपको अपनी सबसे हाल की गर्भावस्था के बारे में प्रश्न पूछने जा रही हूं<br><br>आपकी सबसे हाल की गर्भावस्था के दौरान, क्या आपने किसी भी प्रसवपूर्व देखभाल यात्राओं में भाग लिया था?	1 Yes (हाँ)<br>0 No (नहीं)
anc_place *(required)*	18. Where did you receive antenatal care for this pregnancy?<br><br>(आपने इस गर्भावस्था के लिए कहा से प्रसव पूर्व देखभाल प्राप्त किया था?)<br>*PROBE TO IDENTIFY EACH TYPE OF PERSON AND RECORD ALL MENTIONED<br><br>(व्यक्ति के प्रत्येक प्रकार की पहचान करने के लिए प्रोत्साहित करें और सभी ध्यान दें)*<br>*Question relevant when: selected(${recentpreg_anc} , '1')*	1 Respondent's Home (अपना घर)<br>2 Someone else's home (किसी और का घर)<br>3 Community Health Centre (CHC) सामुदायिक स्वास्थ्य केंद्र (सीएचसी)<br>4 Primary Health Centre (PHC) प्राथमिक स्वास्थ्य केंद्र (पीएचसी)<br>5 Sub-centre (उप-केन्द्र)

		6 Mobile clinic (Govt.) (मोबाइल क्लिनिक (सरकार)<br>11 other Govt. facility (Specify) (अन्य सरकार सुविधा (निर्दिष्ट करें)<br>7 Private Hospital/clinic (निजी अस्पताल / क्लिनिक)<br>8 Maternity Home (प्रसूति गृह)<br>9 Mobile Clinic (Private) (मोबाइल क्लिनिक (निजी)<br>12 other private facility (Specify) (मोबाइल क्लिनिक (निजी)<br>10 Others (Specify) (अन्य (निर्दिष्ट करें)
ancplace_other_pub *(required)*	18.2 Public Sector - Other (Specify)<br><br>(सार्वजनिक क्षेत्र - अन्य (स्पष्ट करें))<br>*Question relevant when: selected(${anc_place} , '11')*	
ancplace_other_pri *(required)*	18.3 Private Sector - Other (Specify)<br><br>(निजी चिकित्सा क्षेत्र - अन्य (स्पष्ट करें))<br>*Question relevant when: selected(${anc_place} , '12')*	
ancplace_specify *(required)*	19. Other (Specify)<br>(अन्य (स्पष्ट करें))<br>*Question relevant when: selected(${anc_place} , '10')*	
month_firstanc *(required)*	20. How many months pregnant were you when you first received antenatal care for this pregnancy? (Months)<br><br>आप कितने माह की गर्भवती थीं जब आप पहली बार इस गर्भावस्था के लिए प्रसव पूर्व देखभाल प्राप्त कर रही थी ? (माह)	
times_anc *(required)*	21. How many times did you receive antenatal care during this pregnancy?<br><br>कितनी बार आपने इस गर्भावस्था के दौरान प्रसव पूर्व देखभाल प्राप्त किया?<br>*If they can't remember ask about how many times*<br>*Response constrained to: regex(.,'^\d{1,30}(?:\d{1,30}){0,29}$')*	
anycompli_preg *(required)*	22. At any time, during your pregnancy did you suffer from any problems?<br><br>किसी भी समय, अपनी गर्भावस्था के दौरान आपको कोई भी समस्या हुई थी?	1 Yes (हाँ)<br>0 No (नहीं)
pregcompli_a *(required)*	23.1. If yes, what problems did you have?<br><br>आपको क्या समस्या हुई?<br>*Probe: Anything else?*<br>*Question relevant when: selected(${anycompli_preg} , '1')*	1 Headache (सरदर्द)<br>2 Blurry vision (धुंधली दृष्टि)<br>3 Edema/pre-eclampsia (सूजन)<br>4 Vaginal bleeding (योनि से खून बहना)<br>5 Convulsions/eclampsia (दौरे पड़ना)<br>6 Tetanus (धनुस्तंभ(टिटनेस))<br>7 Foul-smelling discharge (बदबूदार श्राव)<br>8 Lower abdominal pain (निचले पेट में दर्द)<br>9 Fever (बुखार)<br>10 Excessive vomiting (अत्यधिक उलटी)<br>11 Dizziness (चक्कर आना)<br>12 Palpitation (घबराहट)<br>13 High blood pressure (उच्च रक्त चाप)<br>14 Diabetes (सूगर)<br>15 Anaemia (एनीमिया (खून की कमी))<br>16 Malaria (मलेरिआ)<br>17 Urinary Tract Infection (मूत्र पथ के संक्रमण)<br>18 Other (Specify) (अन्य (स्पष्ट करें))
pregcompli_specify *(required)*	23.2 What problems did you have? Other (Specify)	

	आपको क्या समस्या हुई? - अन्य (स्पष्ट करें)<br>*Question relevant when: selected(${pregcompli_a} , '18')*	
compli_visit_hp *(required)*	24. Did you see anyone about this (these) problems?<br><br>क्या आप इन सब समस्याओं के लिए किसी से मिली थी?<br>*Question relevant when: selected(${anycompli_preg} , '1')*	1 Yes (हाँ)<br>2 No (नहीं)<br>88 Don't Know (पता नहीं)
visited_doc *(required)*	25.1 Who did you see for the problems you had?<br><br>आपने इन समस्याओं के लिए किनसे परामर्श लिया था?<br>*PROBE FOR THE TYPE(S) OF PERSON(S) AND RECORD ALL MENTIONED<br><br><br>व्यक्ति (एस) के प्रकार (एस) के लिए प्रोब और सभी ध्यान दें*<br>*Question relevant when: selected(${compli_visit_hp} , '1')*	1 Doctor (डॉक्टर)<br>2 Nurse/Midwife (नर्स/मिडवाइफ)<br>3 Auxiliary Nurse Midwife (A.N.M.) सहायक नर्स मिडवाइफ<br>4 Trained Traditional Birth Attendant (प्रशिक्षित पारंपरिक जन्म परिचर)<br>5 Untrained Traditional Birth Attendant (अप्रशिक्षित पारंपरिक जन्म परिचर)<br>6 Relative/Friend (रिश्तेदार / दोस्त)<br>10 No one (कोई नहीं)<br>9 Others (Specify) (अन्य)
visited_other *(required)*	25.2 Who did you see about the problems you had? Others (Specify)<br><br>आपने इन समस्यायों के लिए किनसे परामर्श लिया था ? अन्य<br>*Question relevant when: selected(${visited_doc} , '9')*	
deli_place *(required)*	26.1 Why did you choose this facility for your delivery?<br><br>(आपने प्रसब के लिए यह स्वास्थ्य केंद्र क्यों चुना?)<br>*select all that apply*	1 Referred from another facility (दूसरे स्वास्थ्यकेंद्र से भेजा गया)<br>2 Close to my home (मेरे घर के पास है)<br>3 I delivered here in the past (मैं पहले भी प्रसब के लिए यहाँ आई हूँ)<br>4 I visited here for my ANC care (मैंने यहाँ आपनी प्रसब पुर्व देखभाल ली थी)<br>5 Affordable (सस्ती है)<br>6 It provides good care/expert/good care provided by well trained staff (यह अच्छी तरह से देखभाल / विशेषज्ञ / अच्छी तरह से प्रशिक्षित सेवा प्रदान करता है)<br>7 Recommended by a friend/family (दोस्त / परिवार द्वारा सलाह)<br>8 No other choice (कोई और बिकल्प नहीं था)<br>9 Other (Specify) (अन्य (स्पष्ट करें)
deliplace_specify *(required)*	26.2 Why did you choose this facility for your delivery? Other (Specify)<br><br>आपने प्रसब के लिए यह स्वास्थ्य केंद्र क्यों चुना? अन्य (स्पष्ट करें)<br>*Question relevant when: selected(${deli_place} , '9')*	
transport *(required)*	27.1 How did you get to this health facility?<br><br>आप इस स्वास्थ्य केंद्र तक कैसे पहुंचीं ?<br>*Probe: Any other way<br><br>Select all that you apply*	1 Ambulance (एम्बुलेंस)<br>2 Taxi / private car (costs money) टैक्सी / निजी कार (पैसे लागू)<br>3 Private Car (free) (निजी कार (मुफ़्त)<br>4 Public Transport (सार्वजनिक परिवाहन)<br>5 Motorcycle (मोटरसाइकिल)<br>6 Bicycle (साइकिल)<br>7 Walk (पैदल)<br>88 Don't Know (पता नहीं)

		9 Other (Specify) (अन्य (स्पष्ट करें))
transport_specify *(required)*	27.2 How did you get to this health facility? Other (Specify)<br><br>(आप इस स्वास्थ्य केंद्र तक कैसे पहुंचीं ? अन्य (स्पष्ट करें)<br>*Question relevant when: selected(${transport} , '9')*	
time_toreach *(required)*	28.1 About how much time did it take you to get to this facility from where you live?<br><br>आपको यहाँ तक आने में कितना समय लगा ?<br>*Question relevant when: selected(${anycompli_preg} , '1')*	
time_unit *(required)*	28.2 Select minute/hour for the digit entered in the previous question<br><br>उपर भरी अवधि की इकाई बताएं | *Question relevant when: selected(${anycompli_preg} , '1')*	1 Minutes (मिनट)<br>2 Hours (घंटे)
transport_time *(required)*	29. How do you feel about the amount of time it takes you to get to the health facility? Will you say it is very short, a little long, somewhat long, or very long?<br><br>आपको क्या लगता है की, आपको स्वास्थ्य केंद्र पहुँचने के लिए कितना समय लगता है? क्या आप कहंगे की यह बहुत कम, थोड़ा सा, कुछ हद तक है, या बहुत ज्यादा है?	1 Very short (बहुत कम)<br>2 A little long (थोड़ा सा)<br>3 Long (ज्यादा)<br>4 Very long (बहुत ज्यादा)
transport_cost *(required)*	30.1. Did you pay any money for transportation?<br><br>क्या आपने यहां तक पहुँचने के लिए परिवहन के लिए पैसे दिए थे?	1 Yes (हाँ)<br>2 No (नहीं)<br>88 Don't Know (पता नहीं)
transcost_amount *(required)*	30.2 If yes, how much did you pay? (Rs.)<br><br>कितने पैसे दिए ? (Rs.)<br>*Indicate -88 if respondent doesn't know*<br>*Question relevant when: selected(${transport_cost} , '1')*<br>*Response constrained to: .>=1 and .<=9999*	
trans_access *(required)*	31. How easy is it for you to get transportation to the health facility? Would you say it is very easy, easy, difficult, or very difficult?<br><br>स्वास्थ्य केंद्र तक पहुंचने के लिए परिवहन पाना आपके लिए कितना आसान है? क्या आप कहेंगे बहुत आसान, आसान, मुश्किल और बहुत मुश्किल?	1 Very easy (बहुत आसान है)<br>2 Easy (आसान है)<br>3 Difficult (मुश्किल है)<br>4 Very Difficult (बहुत कठिन है)
trans_cost *(required)*	32. How easy is it for you to pay for transportation to the health facility? Would you say it is very easy, easy, difficult, or very difficult?<br><br>स्वास्थ्य केंद्र तक पहुंचने के लिए परिवहन का खर्च उठाना आपके लिए कितना आसान है? क्या आप कहेंगे बहुत आसान, आसान, मुश्किल और बहुत मुश्किल?	1 Very easy (बहुत आसान है)<br>2 Easy (आसान है)<br>3 Difficult (मुश्किल है)<br>4 Very Difficult (बहुत कठिन है)
facility_free *(required)*	33. Is delivery care free in this facility?<br><br>क्या इस स्वास्थ्य केंद्र में प्रसव देखभाल मुफ्त में उपलब्ध है?	1 Yes (हाँ)<br>0 No (नहीं)<br>88 Don't Know (पता नहीं)
delivery_paid *(required)*	34. Did you pay any money to the health provider for delivery of your baby?<br><br>क्या आपने डिलीवरी कराने के लिए स्वास्थ्य प्रदाता को कोई पैसा दिया था?	1 Yes (I know how much I have paid) हाँ (पता है कितना दिया)<br>2 Yes (but don't know how much) हाँ (पर पता नही कितना)<br>0 No नहीं<br>88 Don't Know पता नहीं
delipaid_amount *(required)*	34.2 How much did you pay?<br><br>कितने पैसे दिए ?<br>*Question relevant when: selected(${delivery_paid} , '1')*<br>*Response constrained to: .>=1 and .<=9999*	
child_sex_payment *(required)*	35. Was the amount you paid dependent on the sex of the baby you had ?<br><br>क्या आपके बच्चे के लिंग की वजह से आपको ज्यादा या कम पैसा देना पड़ा?<br>*Question relevant when: selected(${delivery_paid} , '1')*	1 Yes (हाँ)<br>0 No (नहीं)<br>88 Don't Know (पता नहीं)
medi_cost *(required)*	36.1 Did you pay any money for any drugs?<br><br>क्या आपने किसी भी दवाई के लिए कुछ भी पैसे का भुगतान किया था?<br>*This includes drugs purchased for delivery (either within facility or outside).*	1 Yes (I know how much I have paid) हाँ (पता है कितना दिया)<br>2 Yes (but don't know how much) हाँ (पर पता नही कितना)<br>0 No नहीं<br>88 Don't Know पता नहीं
med_pay *(required)*	36.2. How much did you pay? (Rs.)	

	आपने कितने पैसे दिए ? (Rs.)<br>*This includes drugs purchased for delivery (either within facility or outside).<br><br>Indicate -88 if respondent doesn't know*<br>*Question relevant when: selected(${medi_cost} , '1')*<br>*Response constrained to: .>=1 and .<=9999*	
deli_test_cost *(required)*	37. Did you pay any money for tests or supplies?<br><br>क्या आपने परीक्षण या आपूर्ति के लिए किसी भी पैसे का भुगतान किया था?	1 Yes (I know how much I have paid) हाँ (पता है कितना दिया)<br>2 Yes (but don't know how much) हाँ (पर पता नही कितना)<br>0 No नहीं<br>88 Don't Know पता नहीं
deli_test_amount *(required)*	37.2 How much did you pay? (Rs.)<br><br>कितने पैसे दियें ? (Rs.)<br>*Question relevant when: selected(${deli_test_cost} , '1')*<br>*Response constrained to: .>=1 and .<=9999*	
deli_test_expense *(required)*	38. How easy is it for you to get money to buy what you need for your delivery and pay for services at the health facility? Would you say it is very easy, easy, difficult, or very difficult?<br><br>अपनी देखभाल के लिए और दवाइयों/टेस्ट के लिए खर्च उठाना आपके लिए कितना आसान है? क्या यह बहुत आसान है, आसान है, मुश्किल है, बहुत कठिन है?<br>*Question relevant when: not(selected(${delivery_paid} , '2'))*	1 Very easy (बहुत आसान है)<br>2 Easy (आसान है)<br>3 Difficult (मुश्किल है)<br>4 Very Difficult (बहुत कठिन है)
receiv_jsy *(required)*	39. Did/will you receive a cash incentive to deliver in this facility (Janani Suraksha Yojana)?<br><br>क्या आपको यहां डिलीवरी कराने के लिए पैसे मिलेंगे (जननी सुरक्षा योजना)?<br>*Question relevant when: not(selected(${delivery_paid} , '0')) or not(selected(${delivery_paid} , '88'))*	1 Yes (हाँ)<br>0 No (नहीं)<br>88 Don't Know (पता नहीं)
Scale for person-centered quality measures<br><br>Now I am going to ask you some questions about your experiences in the health facility during your last delivery. Remember that all the questions in this section refer specifically to the time you were in the health facility for this last delivery. Also, know that everything you tell me is confidential and will not be shared with the health facility. <br>व्यक्ति केंद्रित गुणवत्ता उपायों के लिए स्केल<br><br>अब मैं आपको अंतिम सुविधा के दौरान स्वास्थ्य सुविधा में अपने अनुभवों के बारे में कुछ प्रश्न पूछने जा रहा हूं।<br>याद रखें कि इस खंड में सभी प्रश्न विशेष रूप से उस समय तक संदर्भित करते हैं जब आप इस अंतिम वितरण के लिए स्वास्थ्य सुविधा में थे। साथ ही, पता है कि जो कुछ भी आप मुझे बताते हैं वह गोपनीय है और स्वास्थ्य सुविधा के साथ साझा नहीं किया जाएगा।<br>*Group relevant when: selected(${witness_consent} , '1')*		
pcc_time *(required)*	40. How did you feel about the amount of time you waited to meet health workers after reaching the facility? Would you say it was very short, somewhat short, somewhat long, or very long?<br><br>आपको इस स्वास्थ्य केंद्र में पहुँचने के बाद स्वास्थ्यकर्मियों से मिलने के लिए जितना समय इंतज़ार करना पड़ा उसके बारे में आपको क्या लगता है कि वह बहुत कम था, थोड़ा कम था, थोड़ा ज़्यादा या बहुत ज़्यादा?	0 Very short (बहुत कम)<br>1 somewhat short (थोड़ा कम)<br>3 somewhat long (थोड़ा ज़्यादा)<br>4 very long (बहुत ज़्यादा)
pcc-introduce-a *(required)*	41.a. Did the doctors, nurses, or other health care providers introduce themselves to you when they came to see you?<br><br>डॉक्टरों, नर्सों, या अन्य स्वास्थ्य देखभाल प्रदाताओं ने आपको आपना खुद का परिचय दिया था ?	0 No, never (नहीं, कभी नहीं)<br>1 Yes, a few times (हाँ, कभी कभी)<br>2 Yes, most of the times (हाँ, ज़्यादातर)<br>3 Yes, all the time (हाँ, हमेशा)
note_1	We just asked about how often providers introduced themselves to you. We will now ask you a question that sounds very similar. However, we are interested in learning how many providers introduced themselves to you.<br><br>हमने अभी पूछा है कि प्रदाताओं ने आपको कितनी बार पेश किया। अब हम आपको एक प्रश्न पूछेंगे जो बहुत समान लगता है। हालांकि, हम सीखने में रुचि रखते हैं कि कितने प्रदाताओं ने आपको खुद को पेश किया	
pcc_introduce *(required)*	41. b. "During your time in the health facility did the doctors, nurses, or other health care providers introduce themselves to you when they first came to see you?<br><br>(If yes) Was it a few of them, most of them, or all of them,"<br><br>जब आप स्वास्थ्य केंद्र में थीं, तब क्या पहली मुलाकात में डॉक्टरों, नर्स, अथवा अन्य स्वास्थ्यकर्मियों ने आपको अपना परिचय दिया था? (यदि हाँ, तो क्या कुछ ने दिया, ज़्यादातर ने दिया या सभी ने दिया)	0 No, none of them (नहीं, किसी ने नहीं दिया था)<br>1 Yes, a few of them (हाँ, कुछ ने दिया था)<br>2 Yes, most of them (हाँ, ज़्यादातर ने दिया था)<br>3 Yes, all of them (हाँ, सभी ने दिया था)
pcc_respect *(required)*	Now I will ask you some questions about how you were treated at the health facility. Tell me if the following things happened all the time, most of the time, a few times, or it never happened. You can say a few times if it happened one or two times, and most of the time will be if it happened 3 or more times, but not always. For some questions I will ask specifically if something occurred during labor, delivery, or after delivery. If I do not specify please answer based on your experiences during the entire time you were in the facility from labor until discharge.<br><br>42. Did the doctors, nurses, or other staff at the facility treat you with respect?<br><br>क्या डॉक्टर/ नर्स ने आपके साथ सम्मान के साथ व्यवहार किया?	0 No, never (नहीं, कभी नहीं)<br>1 Yes, a few times (हाँ, कभी कभी)<br>2 Yes, most of the times (हाँ, ज़्यादातर)<br>3 Yes, all the time (हाँ, हमेशा)

	(PROBE FOR ALL QUESTIONS: if respondent just responds, yes, ask them: Did this occur a few times, most of the time, or all the time)? DO NOT PROMPT RESPONDENT	
pcc_name *(required)*	43. Did the doctors, nurses, or other health care providers call you by your name?<br><br>क्या डॉक्टर, नर्स, या अन्य स्वास्थयकर्मी आपको आपके नाम से पुकारते थे?	0 No, never (नहीं, कभी नहीं)<br>1 Yes, a few times (हाँ, कभी कभी)<br>2 Yes, most of the times (हाँ, ज़्यादातर)<br>3 Yes, all the time (हाँ, हमेशा)
pcc_friendly *(required)*	44. Did the doctors, nurses, and other staff at the facility treat you in a friendly manner?<br><br>क्या डॉक्टर,नर्स या अन्य स्वास्थयकर्मियों का व्यवहार प्रेमपूर्वक था?	0 No, never (नहीं, कभी नहीं)<br>1 Yes, a few times (हाँ, कभी कभी)<br>2 Yes, most of the times (हाँ, ज़्यादातर)<br>3 Yes, all the time (हाँ, हमेशा)
pcc_privacy_a *(required)*	45.a. Were you covered up with a cloth, or blanket during examinations in the labor room so that you did not feel exposed?<br><br>क्या आप जाँच के दौरान कपड़े, या कंबल से ढके थे ताकि आप को खुला ना महसूश हो?<br>*We just asked about how you were may or may not have been covered in the facility during examinations. We will now ask you the same question, but it also includes whether or not a curtain was used to screen you in the facility.*	0 No, never (नहीं, कभी नहीं)<br>1 Yes, a few times (हाँ, कभी कभी)<br>2 Yes, most of the times (हाँ, ज़्यादातर)<br>3 Yes, all the time (हाँ, हमेशा)
pcc_priv_vis *(required)*	45. b. During examinations in the labor room, were you covered up with a cloth or blanket or screened with a curtain so that you did not feel exposed?<br><br>प्रसव कक्ष में जब आपकी जांच करी गयी तब क्या आपको चादर या कम्बल से ढाका गया था या कोई पर्दा डाला गया था ताकि आपको ना लागा हो की आपका सरीर खुला हुआ है	0 No, never (नहीं, कभी नहीं)<br>1 Yes, a few times (हाँ, कभी कभी)<br>2 Yes, most of the times (हाँ, ज़्यादातर)<br>3 Yes, all the time (हाँ, हमेशा)
pcc_info_confidential *(required)*	46..Do you feel like your health information was or will be kept confidential at this facility?<br><br>क्या आपको लगता है कि इस स्वास्थ्य केंद्र में आपकी जो भी स्वास्थ्य सम्बंधित सूचना ली गयी है वह गोपनीय है व रहेगी?	0 No Never (नहीं कभी नहीं)<br>1 Yes few times (हाँ कभी कभी)<br>2 Yes most of the time (हां ज्यादा तर)<br>3 Yes all the time (हाँ हमेशा)
pcc_involvement *(required)*	47. Did you feel like the doctors, nurses or other staff at the facility involved you in decisions about your care?<br><br>क्या आपको लगता है कि इस स्वास्थ्य केंद्र के डाक्टर, नर्स, व स्वास्थ्यकर्मियों ने आपको आपके स्वास्थ्य सम्बंधित फैसलों में शामिल रखा था?	0 No. Never (नहीं कभी नहीं)<br>1 Yes, a few times (हाँ कभी कभी)<br>2 Yes, Most of the time (हाँ ज्यादा तर)<br>3 Yes, all of the time (हाँ हमेशा)
pcc_permission *(required)*	48. Did the doctors, nurses or other health staff at the facility ask your permission/consent before doing procedures on you?<br><br>आप के ऊपर कोई भी प्रक्रिया करने से पहले क्या इस केंद्र के डाक्टर, नर्स, व स्वास्थ्यकर्मियों ने आपसे अनुमति या सहमति ली थी ?	0 No, never (नहीं, कभी नहीं)<br>1 Yes, a few times (हाँ, कभी कभी)<br>2 Yes, most of the times (हाँ, ज़्यादातर)<br>3 Yes, all the time (हाँ, हमेशा)
pcc_position_choice_a *(required)*	49.a. During labour and delivery, some women like laying down, while others like to sit, stand or walk around. During labour and delivery, do you feel like you were able to be in the position of your choice?<br><br>प्रसव के दौरान, कुछ महिलाएं नीचे लेटना पसंद करती हैं, जबकि अन्य बैठना, खड़े होकर घूमना पसंद करते हैं। प्रसव के दौरान, क्या आपको लगता है कि आप अपनी पसंद की स्थिति में सक्षम थे?<br>*We just asked about whether you were able to be in the position of your choice during labor and delivery. We will now ask you the same question, but we want to know if you were able to be in the position of your choice for delivery only.<br><br>(हमने अभी पूछा है कि क्या आप प्रसव के दौरान आपकी पसंद की स्थिति में हैं। अब हम आपको एक ही प्रश्न पूछेंगे, लेकिन हम जानना चाहते हैं कि क्या आप केवल वितरण के लिए अपनी पसंद की स्थिति में हैं।)*	0 No, never (नहीं, कभी नहीं)<br>1 Yes, a few times (हाँ, कभी कभी)<br>2 Yes, most of the times (हाँ, ज़्यादातर)<br>3 Yes, all the time (हाँ, हमेशा)
pcc_position_choice_b *(required)*	49.b. During the delivery, do you feel like you were able to be in the position of your choice?<br><br>प्रसव के समय, कभी-कभी महिलाओं को कोई स्थिति ज्यादा आरामदेह लगती है. क्या आपको ऐसा लगा की आप प्रसव के दौरन अपने आराम के अनुसार स्थिति में बैठ या लेट पा रही थीं?	0 No, never (नहीं, कभी नहीं)<br>1 Yes, a few times (हाँ, कभी कभी)<br>2 Yes, most of the times (हाँ, ज़्यादातर)<br>3 Yes, all the time (हाँ, हमेशा)
pcc_language *(required)*	50. Did the doctors, nurses or health staff at the facility speak to you in a language you could understand?<br><br>क्या केंद्र के डाक्टर, नर्स, और बाकी कर्मचारी आपसे ऐसी भाषा में बात करते हैं कि जो आपको आसानी से समझ में आ जाती है?	0 No, never (नहीं, कभी नहीं)<br>1 Yes, a few times (हाँ, कभी कभी)<br>2 Yes, most of the times (हाँ, ज़्यादातर)<br>3 Yes, all the time (हाँ, हमेशा)
pcc_explain_exams *(required)*	51. Did the doctors and nurses explain to you why they were doing examinations or procedures on you?<br><br>क्या डाक्टरों और नर्स ने आपको समझाया कि वह आपकी कोई भी जांच या प्रक्रिया क्यों कर रहे हैं?	0 No, never (नहीं, कभी नहीं)<br>1 Yes, a few times (हाँ, कभी कभी)<br>2 Yes, most of the times (हाँ, ज़्यादातर)<br>3 Yes, all the time (हाँ, हमेशा)

pcc_explain_meds *(required)*	52. Did the doctors and nurses explain to you why they were giving you any medicine?<br><br>क्या डॉक्टरों और नर्सों नें आपको समझाया कि वह कोई भी दवाई आपको क्यों दे रहे हैं?	0 No, never (नहीं, कभी नहीं)<br>1 Yes, a few times (हाँ, कभी कभी)<br>2 Yes, most of the times (हाँ, ज़्यादातर)<br>3 Yes, all of the time (हाँ, हमेशा)<br>4 Did not get any medicine (कोई भी दवाई नही दी गई)
pcc_feeling *(required)*	53. Did the doctors and nurses at the facility talk to you about how you were feeling?<br><br>क्या स्वास्थ्य केंद्र के डॉक्टरों और नर्सों ने आपसे आपकी तबियत के बारें में बात चीत करी?	0 No, never (नहीं, कभी नहीं)<br>1 Yes, a few times (हाँ, कभी कभी)<br>2 Yes, most of the times (हाँ, ज़्यादातर)<br>3 Yes, all the time (हाँ, हमेशा)
pcc_questions *(required)*	54. Did you feel you could ask the doctors, nurses or other staff at the facility any questions you had?<br><br>क्या आप अपने स्वास्थ्य सम्बंधित कोई भी सवाल किसी भी डॉक्टर, नर्स, या स्वास्थ्य कर्मचारी से पूछ सकती थीं?	0 No, never (नहीं, कभी नहीं)<br>1 Yes, a few times (हाँ, कभी कभी)<br>2 Yes, most of the times (हाँ, ज़्यादातर)<br>3 Yes, all the time (हाँ, हमेशा)
pcc_labor_support *(required)*	55. Were you allowed to have someone you wanted (from outside of staff at the facility, such as family or friends) to stay with you during labor?<br><br>प्रसव पीड़ा के दौरान, क्या आपको अपने किसी सम्बन्धी जैसे परिवार का कोई सदस्य या दोस्त को अपने साथ प्रसव कक्ष में रखने की अनुमति थी?<br>*We are talking about if she was allowed, not if she wanted someone or not.*	0 No, never (नहीं, कभी नहीं)<br>1 Yes, a few times (हाँ, कभी कभी)<br>2 Yes, most of the times (हाँ, ज़्यादातर)<br>3 Yes, all of the time (हाँ, हमेशा)<br>4 I did not want someone to stay with me (मैं नहीं चाहती थी कि कोई मेरे साथ रहे)
pcc_support_del *(required)*	56. Were you allowed to have someone you wanted to stay with you during delivery?<br><br>प्रसव के दौरान, क्या आपको अपने किसी सम्बन्धी जैसे परिवार का कोई सदस्य या दोस्त को अपने साथ प्रसव कक्ष में रखने की अनुमति थी?<br>*We are talking about if she was allowed, not if she wanted someone or not.*	0 No, never (नहीं, कभी नहीं)<br>1 Yes, a few times (हाँ, कभी कभी)<br>2 Yes, most of the times (हाँ, ज़्यादातर)<br>3 Yes, all of the time (हाँ, हमेशा)<br>4 I did not want someone to stay with me (मैं नहीं चाहती थी कि कोई मेरे साथ रहे)
pcc_attention_help *(required)*	57. When you needed help, did you feel the doctors, nurses or other staff at the facility paid attention?<br><br>जब भी आपको किसी प्रकार की सहायता की ज़रुरत पड़ी, तब डॉक्टर, नर्स, या अन्य कोई स्वास्थ्यकर्मी केंद्र पर आपकी सहायता के लिए उपलब्ध थे?	0 No, never (नहीं, कभी नहीं)<br>1 Yes, a few times (हाँ, कभी कभी)<br>2 Yes, most of the times (हाँ, ज़्यादातर)<br>3 Yes, all the time (हाँ, हमेशा)
pcc_controlpain *(required)*	58. Do you feel the doctors or nurses did everything they could to help control your pain?<br><br>क्या आपको लगता है कि आपकी पीड़ा कम करने के लिए डॉक्टर व नर्स ने सभी संभव प्रयास करे?	0 No, never (नहीं, कभी नहीं)<br>1 Yes, a few times (हाँ, कभी कभी)<br>2 Yes, most of the times (हाँ, ज़्यादातर)<br>3 Yes, all the time (हाँ, हमेशा)
pcc_abuse_verbal *(required)*	59. ""Did you feel the doctors, nurses, or other health providers shouted at you, scolded, insulted, threatened, or talked to you rudely?<br><br>(If yes) will you say this happened once, a few times, or many times.""""<br><br>क्या आपको कभी भी लगा कि इस स्वास्थ्य केंद्र के डॉक्टर, नर्स, व अन्य स्वास्थ्यकर्मी ने आपके ऊपर चिल्लाया, डांटा, अपमानित किया, धमकाया, अपशब्द कहा या दुर्व्यवहार किया?	0 No, never (नहीं, कभी नहीं)<br>1 Yes, a few times (हाँ, कभी कभी)<br>2 Yes, most of the times (हाँ, ज़्यादातर)<br>3 Yes, all the time (हाँ, हमेशा)
pcc_physical_abuse *(required)*	60. "Did you feel like you were treated roughly like pushed, beaten, slapped, pinched, physically restrained, or gagged?<br><br>(If yes) will you say this happened once, a few times, or many times."<br><br>""क्या किसी ने आपसे शारीरिक रूप से दुर्व्यवहार किया जैसे कि आपको धक्का दिया, मारा, पीटा, नोचा, या हाथ पाँव बाँध दिया?<br><br>(अगर हाँ, तो ऐसा कितनी बार हुआ होगा?)""""	0 No, never (नहीं, कभी नहीं)<br>1 Yes, a few times (हाँ, कभी कभी)<br>2 Yes, most of the times (हाँ, ज़्यादातर)<br>3 Yes, all the time (हाँ, हमेशा)
pcc_enoughstaff *(required)*	61. Do you think there was enough health staff in the facility to care for you?	0 No, never (नहीं, कभी नहीं)<br>1 Yes, a few times (हाँ, कभी कभी)

	""क्या स्वास्थ्य केंद्र में आपकी देखभाल करने के लिए पर्याप्त स्वास्थ्यकर्मी थे? ""	2 Yes, most of the times (हाँ, ज़्यादातर)<br>3 Yes, all the time (हाँ, हमेशा)
pcc_bestcare *(required)*	62. Did you feel the doctors, nurses or other staff at the facility took the best care of you?<br><br>क्या आपको लगता है कि स्वास्थ्य केंद्र के डॉक्टर, नर्स व अन्य कर्मचारियों ने आपकी सभी संभव देख रेख करी?	0 No, never (नहीं, कभी नहीं)<br>1 Yes, a few times (हाँ, कभी कभी)<br>2 Yes, most of the times (हाँ, ज़्यादातर)<br>3 Yes, all the time (हाँ, हमेशा)
pcc_trust *(required)*	63. Did you feel you could completely trust the doctors, nurses or other staff at the facility with regards to your care?<br><br>क्या आपको लगता है कि आप स्वास्थ्य केंद्र के डॉक्टर, नर्स, और अन्य कर्मचारियों के ऊपर अपनी देखभाल के लिए पूरी तरह भरोसा कर सकती थी?	0 No, never (नहीं, कभी नहीं)<br>1 Yes, a few times (हाँ, कभी कभी)<br>2 Yes, most of the times (हाँ, ज़्यादातर)<br>3 Yes, all the time (हाँ, हमेशा)
pcc_bribe *(required)*	64. Did the doctors, nurses or other staff at the facility ask you or your family for money other than the official cost?<br><br>अधिकृत धनराशि के अलावा, क्या केंद्र के डॉक्टर, नर्स या अन्य कर्मचारियों ने आपसे या आपके परिवार से किसी भी चीज़ के लिए पैसे मांगे?	0 No, never (नहीं, कभी नहीं)<br>1 Yes, a few times (हाँ, कभी कभी)<br>2 Yes, most of the times (हाँ, ज़्यादातर)<br>3 Yes, all the time (हाँ, हमेशा)
pcc_clean_b *(required)*	65.a. Did you think the toilets and washrooms were clean?<br><br>क्या आपको लगता है कि शौचालय और वाशरूम साफ थे?	0 No, never (नहीं, कभी नहीं)<br>1 Yes, a few times (हाँ, कभी कभी)<br>2 Yes, most of the times (हाँ, ज़्यादातर)<br>3 Yes, all of the time (हाँ, हमेशा)<br>4 Not applicable (लागू नहीं)
note_2	We just asked about you opinion related to how clean the toilets and washrooms. We now will ask you a similar question, but we want to know specifically whether you thought the facility was clean overall.<br><br>शौचालयों और वाशरूम को साफ करने के तरीके से हमने आपके बारे में सिर्फ राय के बारे में पूछा। अब हम आपको एक समान प्रश्न पूछेंगे, लेकिन हम विशेष रूप से जानना चाहते हैं कि आपने सोचा था कि सुविधा समग्र रूप से साफ थी।	
pcc_clean_a *(required)*	65. b. ""Thinking about the wards,<br>washrooms and the general environment of the health facility, will you say the facility was very clean, clean, dirty, or very dirty""""<br><br>इस स्वास्थ्य केंद्र के कमरे, शौचालय, और आस पास की जगहों के रख-रखाव को देख कर आप क्या कहेंगी?	0 Very dirty (बहुत गन्दा है)<br>1 Dirty (गन्दा है)<br>2 Clean (साफ़ है)<br>3 Very clean (बहुत साफ़ है)
pcc_safe *(required)*	66. In general, did you feel safe in the health facility?<br><br>क्या आप इस स्वास्थ्य केंद्र में सुरक्षित महसूस करती हैं?	0 No, never (नहीं, कभी नहीं)<br>1 Yes, a few times (हाँ, कभी कभी)<br>2 Yes, most of the times (हाँ, ज़्यादातर)<br>3 Yes, all the time (हाँ, हमेशा)
pcc_explain_purpose *(required)*	67. Did you feel you understood the purpose of medicines and tests given/done to you?<br><br>क्या आपको समझ आ रहा था की आपको अलग-अलग दवाइयां या टेस्ट क्यों दिए जा रहे हैं?	0 No, never (नहीं, कभी नहीं)<br>1 Yes, a few times (हाँ, कभी कभी)<br>2 Yes, most of the times (हाँ, ज़्यादातर)<br>3 Yes, all of the time (हाँ, हमेशा)<br>4 Not applicable (लागू नहीं)
pcc_cleanPNCW *(required)*	68. Did you feel the postnatal ward was clean?<br><br>क्या आपको लगा की प्रसवोत्तर वार्ड साफ़ था?	0 No, never (नहीं, कभी नहीं)<br>1 Yes, a few times (हाँ, कभी कभी)<br>2 Yes, most of the times (हाँ, ज़्यादातर)<br>3 Yes, all of the time (हाँ, हमेशा)<br>4 Not applicable (लागू नहीं)
pcc_ask_pain *(required)*	69. Did you feel like the doctor or nurse asked how much pain your were in?<br><br>क्या आपको लगा की डॉक्टर / नर्स ने आपके दर्द के बारे मैं आपसे पुछा?	0 No, never (नहीं, कभी नहीं)<br>1 Yes, a few times (हाँ, कभी कभी)<br>2 Yes, most of the times (हाँ, ज़्यादातर)<br>3 Yes, all the time (हाँ, हमेशा)
pcc_language1 *(required)*	70. When you had questions, did the doctor or nurse answer in a way you could understand?<br><br>जब आपने कोई सवाल पूछे, क्या डॉक्टर /नर्स ने जवाब ऐसी तरह दिए की आप पूरी तरह समझ पायीं?	0 No, never (नहीं, कभी नहीं)<br>1 Yes, a few times (हाँ, कभी कभी)<br>2 Yes, most of the times (हाँ, ज़्यादातर)

		3 Yes, all the time (हाँ, हमेशा)
pcc_medioutside *(required)*	71. Were you or your family asked to buy anything from outside the health facility for your care?<br><br>क्या आपको या आपके परिवार को स्वास्थ्य केंद्र के बाहर से आपके इलाज से सम्बंधित कोई सामान या टेस्ट करवाने को कहा गया था?	0 No, never (नहीं, कभी नहीं)<br>1 Yes, a few times (हाँ, कभी कभी)<br>2 Yes, most of the times (हाँ, ज़्यादातर)<br>3 Yes, all the time (हाँ, हमेशा)
pcc_painmeds_needed *(required)*	72. Did you feel you were you given pain medication when you felt you needed it?<br><br>क्या आपको लगा की ज़रुरत पड़ने पर आपको दर्द की दवा दी गयी थी ?	0 No, never (नहीं, कभी नहीं)<br>1 Yes, a few times (हाँ, कभी कभी)<br>2 Yes, most of the times (हाँ, ज़्यादातर)<br>3 Yes, all the time (हाँ, हमेशा)
pcc_helptoilet *(required)*	73. When you had to go to the toilet or washroom, did someone help you?<br><br>टॉयलेट/ बाथरूम जाने के समय क्या किसी ने आपकी सहायता की?	0 No, never (नहीं, कभी नहीं)<br>1 Yes, a few times (हाँ, कभी कभी)<br>2 Yes, most of the times (हाँ, ज़्यादातर)<br>3 Yes, all of the time (हाँ, हमेशा)<br>4 Not applicable (लागू नहीं)
pcc_cleanLW *(required)*	74. Did you feel the labour ward was clean?<br><br>क्या आपको लगा की लेबर वार्ड साफ़ था?	0 No, never (नहीं, कभी नहीं)<br>1 Yes, a few times (हाँ, कभी कभी)<br>2 Yes, most of the times (हाँ, ज़्यादातर)<br>3 Yes, all the time (हाँ, हमेशा)
Scale for person-centered quality measures<br><br>Now I am going to ask you some questions about your experiences in the health facility during your last delivery. Remember that all the questions in this section refer specifically to the time you were in the health facility for this last delivery. Also, know that everything you tell me is confidential and will not be shared with the health facility. <br>व्यक्ति केंद्रित गुणवत्ता उपायों के लिए स्केल<br><br>अब मैं आपको अंतिम सुविधा के दौरान स्वास्थ्य सुविधा में अपने अनुभवों के बारे में कुछ प्रश्न पूछने जा रहा हूं।<br>याद रखें कि इस खंड में सभी प्रश्न विशेष रूप से उस समय तक संदर्भित करते हैं जब आप इस अंतिम वितरण के लिए स्वास्थ्य सुविधा में थे। साथ ही, पता है कि जो कुछ भी आप मुझे बताते हैं वह गोपनीय है और स्वास्थ्य सुविधा के साथ साझा नहीं किया जाएगा। > During your labor and delivery would you say you were treated differently because of any of the following?<br><br>क्या प्रसव या डिलिवरी के दौरान आपके साथ किसी भी वजह से किसी तरह का भेदभाव हुआ था ।		
pcc_age *(required)*	75. During your labor and delivery would you say you were treated differently because of any of the following?<br><br>Your age?<br><br>क्या प्रसव या डिलिवरी के दौरान आपके साथ किसी भी वजह से किसी तरह का भेदभाव हुआ था<br>।<br>आपकी उम्र ?	0 No (नहीं)<br>1 Yes, Better (हाँ , बेहतर)<br>2 Yes, Worse (हाँ , खराब)
pcc_maritalstatus *(required)*	76. During your labor and delivery would you say you were treated differently because of any of the following?<br><br>Your marital status?<br><br>आपकी वैवाहिक स्थिति	0 No (नहीं)<br>1 Yes, Better (हाँ , बेहतर)<br>2 Yes, Worse (हाँ , खराब)
pcc_no_children *(required)*	77. During your labor and delivery would you say you were treated differently because of any of the following?<br><br>The number of children you have?<br><br>आपके बच्चों की संख्या	0 No (नहीं)<br>1 Yes, Better (हाँ , बेहतर)<br>2 Yes, Worse (हाँ , खराब)
pcc_sex *(required)*	78. During your labor and delivery would you say you were treated differently because of any of the following?<br><br>Sex of your newborn?<br><br>शिशु का लिंग	0 No (नहीं)<br>1 Yes, Better (हाँ , बेहतर)<br>2 Yes, Worse (हाँ , खराब)
pcc_delioutcome *(required)*	79. During your labor and delivery would you say you were treated differently because of any of the following?<br><br>The outcome of your pregnancy?<br><br>आपकी गर्भावस्था का परिणाम	0 No (नहीं)<br>1 Yes, Better (हाँ , बेहतर)<br>2 Yes, Worse (हाँ , खराब)
pcc_economicstatus1 *(required)*	80. Your economic status (wealth or lack of money)?<br><br>आपकी आर्थिक स्थिति (अमीर या गरीब होना)	0 No (नहीं)<br>1 Yes, Better (हाँ , बेहतर)<br>2 Yes, Worse (हाँ , खराब)
pcc_education *(required)*	81. The level of your education?<br><br>आपकी शिक्षा का स्तर	0 No (नहीं)<br>1 Yes, Better (हाँ , बेहतर)<br>2 Yes, Worse (हाँ , खराब)
pcc_socialstatus *(required)*	82. Your social status?	0 No (नहीं)

	आपकी सामाजिक स्थिति	1 Yes, Better (हाँ , बेहतर)<br>2 Yes, Worse (हाँ , खराब)
pcc_religion *(required)*	83. Your religion?<br><br>आपका धर्म	0 No (नहीं)<br>1 Yes, Better (हाँ , बेहतर)<br>2 Yes, Worse (हाँ , खराब)
pcc_caste *(required)*	84. Your caste?<br><br>आपकी जाति	0 No (नहीं)<br>1 Yes, Better (हाँ , बेहतर)<br>2 Yes, Worse (हाँ , खराब)
pcc_connection *(required)*	85. Your connections with the facility?<br><br>स्वास्थ्य केंद्र में आपकी जान पहचान	0 No (नहीं)<br>1 Yes, Better (हाँ , बेहतर)<br>2 Yes, Worse (हाँ , खराब)
pcc_diseasestatus *(required)*	86. Any illnesses you may have?<br><br>आपकी कोई बीमारी	0 No (नहीं)<br>1 Yes, Better (हाँ , बेहतर)<br>2 Yes, Worse (हाँ , खराब)
pcc_opinion *(required)*	87. Your difference in opinion?<br><br>आपकी डॉक्टरों से अलग राय	0 No (नहीं)<br>1 Yes, Better (हाँ , बेहतर)<br>2 Yes, Worse (हाँ , खराब)

Scale for person-centered quality measures

Now I am going to ask you some questions about your experiences in the health facility during your last delivery.
Remember that all the questions in this section refer specifically to the time you were in the health facility for this last delivery. Also, know that everything you tell me is confidential and will not be shared with the health facility.

व्यक्ति केंद्रित गुणवत्ता उपायों के लिए स्केल

अब मैं आपको अंतिम सुविधा के दौरान स्वास्थ्य सुविधा में अपने अनुभवों के बारे में कुछ प्रश्न पूछने जा रहा हूं।
याद रखें कि इस खंड में सभी प्रश्न विशेष रूप से उस समय तक संदर्भित करते हैं जब आप इस अंतिम वितरण के लिए स्वास्थ्य सुविधा में थे। साथ ही, पता है कि जो कुछ भी आप मुझे बताते हैं वह गोपनीय है और स्वास्थ्य सुविधा के साथ साझा नहीं किया जाएगा। > In the following questions, I will ask you if you think it is acceptable for certain things to happen during your labour and delivery. Please tell me if is acceptable in all instances. acceptable in only certain instances, or not acceptable in all instances"

निम्नलिखित प्रश्नों में, मैं आपसे पूछूंगा कि क्या आपको लगता है कि आपके श्रम और प्रसव के दौरान कुछ चीजें होने के लिए स्वीकार्य है। कृपया मुझे बताएं कि सभी मामलों में स्वीकार्य है या नहीं। केवल कुछ मामलों में स्वीकार्य है, या सभी मामलों में स्वीकार्य नहीं है "

pcc_confidential1 *(required)*	88. Personal information kept confidential.<br><br>निजी जानकारी का गोपनीय रखा जाना।	1 Unacceptable in all instances (कभी नहीं होना चाहिए।)<br>2 Acceptable in certain instances (कभी कभी होना ठीक है।)<br>3 Acceptable in all instances (हमेशा होना चाहिए।)
pcc_waitingtime *(required)*	89. To wait for more than one hour before being seen<br><br>डॉक्टर को दिखवाने के लिए एक घंटे से ज़्यादा इंतज़ार करना।	1 Unacceptable in all instances (कभी नहीं होना चाहिए।)<br>2 Acceptable in certain instances (कभी कभी होना ठीक है।)<br>3 Acceptable in all instances (हमेशा होना चाहिए।)
pcc_scolding *(required)*	90 Providers to shout at or scold the patient<br><br>डॉक्टर / नर्स मरीज़ पे चिल्लाएं या उसको डांटा |	1 Unacceptable in all instances (कभी नहीं होना चाहिए।)<br>2 Acceptable in certain instances (कभी कभी होना ठीक है।)<br>3 Acceptable in all instances (हमेशा होना चाहिए।)
pcc_beating *(required)*	91. Providers hit the patient if they don't do what they are told<br><br>कहना न मानने पर डॉक्टर / नर्स ने मरीज़ को मारा	1 Unacceptable in all instances (कभी नहीं होना चाहिए।)<br>2 Acceptable in certain instances (कभी कभी होना ठीक है।)<br>3 Acceptable in all instances (हमेशा होना चाहिए।)
pcc_decision *(required)*	92. Not to have a choice of who should be with me during labor and deliver<br><br>प्रसव पीड़ा और डिलीवरी के दौरान मेरे साथ कौन हो ये चुनने का अधिकार न होना।	1 Unacceptable in all instances (कभी नहीं होना चाहिए।)<br>2 Acceptable in certain instances (कभी कभी होना ठीक है।)<br>3 Acceptable in all instances (हमेशा होना चाहिए।)
pcc_clean1 *(required)*	93. For facilities to be dirty<br><br>स्वास्थ्य केंद्र का गन्दा होना।	1 Unacceptable in all instances (कभी नहीं होना चाहिए।)<br>2 Acceptable in certain instances (कभी कभी होना ठीक है।)<br>3 Acceptable in all instances (हमेशा होना चाहिए।)

pcc_bettercare_age *(required)*	94. To receive better care because of age<br><br>उम्र की वजह से बेहतर सेवा मिलना।	1	Unacceptable in all instances (कभी नहीं होना चाहिए।)
		2	Acceptable in certain instances (कभी कभी होना ठीक है।)
		3	Acceptable in all instances (हमेशा होना चाहिए।)
pcc_marital *(required)*	95. To receive better care because you are married<br><br>विवाहित होने की वजह से बेहतर सेवा मिलना।	1	Unacceptable in all instances (कभी नहीं होना चाहिए।)
		2	Acceptable in certain instances (कभी कभी होना ठीक है।)
		3	Acceptable in all instances (हमेशा होना चाहिए।)
pcc_bettercare_education *(required)*	96. To receive better care because you are more educated<br><br>शिक्षित होने की वजह से बेहतर सेवा मिलना।	1	Unacceptable in all instances (कभी नहीं होना चाहिए।)
		2	Acceptable in certain instances (कभी कभी होना ठीक है।)
		3	Acceptable in all instances (हमेशा होना चाहिए।)
pcc_bettercare_economicstatus *(required)*	97. To receive better care because you are wealthy<br><br>अमीर होने की वजह से बेहतर सेवा मिलना।	1	Unacceptable in all instances (कभी नहीं होना चाहिए।)
		2	Acceptable in certain instances (कभी कभी होना ठीक है।)
		3	Acceptable in all instances (हमेशा होना चाहिए।)
pcc_extrapayment *(required)*	98. Health providers ask me or my family for money other than the official cost<br><br>स्वास्थ्य केंद्र वाले इलाज के खर्चे के अलावा पैसे मांगते हैं.	1	Unacceptable in all instances (कभी नहीं होना चाहिए।)
		2	Acceptable in certain instances (कभी कभी होना ठीक है।)
		3	Acceptable in all instances (हमेशा होना चाहिए।)
treated_differently *(required)*	99. To be treated differently if you come to the facility with your partner?<br><br>(यदि आप अपने साथी के साथ स्वास्थ केंद्र में आते हैं तो अलग से इलाज किया जाना चाहिए)	1	Unacceptable in all instances (कभी नहीं होना चाहिए।)
		2	Acceptable in certain instances (कभी कभी होना ठीक है।)
		3	Acceptable in all instances (हमेशा होना चाहिए।)
Pregnancy and Childbirth History – Referral <br>(गर्भावस्था और प्रसव इतिहास - रेफरल)<br>*Group relevant when: selected(${witness_consent} , '1')*			
deli_referred *(required)*	100. "Now I would like to ask questions about referral. When we say referral we are talking about from one facility to another. We do not mean referral within the same facility."<br><br>अब हम सर्वेक्षण के रेफ़रल सेक्शन के साथ शुरू करेंगे, अब मैं रेफ़रल के बारे में प्रश्न पूछना चाहता हूं। जब हम रेफरल कहते हैं तो हम एक सुविधा से दूसरे सुविधा के बारे में बात कर रहे हैं। हम एक ही सुविधा के भीतर रेफरल का मतलब नहीं है। "<br><br>Were you at any point during labor and delivery referred from one place to another?<br><br>बच्चा होने के समय क्या आपको कभी भी एक जगह से दूसरी जगह जाने को कहा गया था?	1	Yes (हाँ)
		0	No (नहीं)
		88	Don't Know (पता नहीं)
referred *(required)*	101. Why were you referred?<br><br>ऐसा क्यों कहा गया था?<br>*Question relevant when: selected(${deli_referred} , '1')*	1	Delivery staff not available स्वास्थ्य सहयोगी नहीं थे
		2	Medicines were unavailable (दवाइयाँ नहीं थीं)
		3	Blood was not available (खून नहीं था)
		4	Cesarean-section was not possible (सीज़ेरियन सेक्शन के लिए कोई नहीं था)
		5	Other surgery was not possible (अन्य सर्जरी नहीं हो सकी)
		6	Complications with delivery (Specify) (बच्चा होने के समय अन्य

		उलझनें (स्पष्टकरें)<br>88 Don't Know (पता नहीं)<br>9 Others (Specify) (अन्य (स्पष्ट करें))
referral_other_specify *(required)*	102. Why were you referred? Other (Specify)<br><br>ऐसा क्यों कहा गया था? बच्चा होने के समय अन्य उलझनें (स्पष्टकरें)<br>*Question relevant when: selected(${referred} , '9')*	
referred_from_place *(required)*	103.1 What type of place were you referred from?<br><br>आपको कौन सी दूसरी जगह पर भेजा गया था?<br>*Clarify with woman what type of facility she started at before being refereed*<br>*Question relevant when: selected(${deli_referred} , '1')*	1 Respondent's Home (अपना घर)<br>2 Someone else's home (किसी और का घर)<br>3 Community Health Centre (CHC) सामुदायिक स्वास्थ्य केंद्र (सीएचसी)<br>4 Primary Health Centre (PHC) प्राथमिक स्वास्थ्य केंद्र (पीएचसी)<br>5 Sub-centre (उप-केन्द्र)<br>6 Mobile clinic (Govt.) (मोबाइल क्लिनिक (सरकार)<br>11 other Govt. facility (Specify) (अन्य सरकार सुविधा (निर्दिष्ट करें)<br>7 Private Hospital/clinic (निजी अस्पताल / क्लिनिक)<br>8 Maternity Home (प्रसूति गृह)<br>9 Mobile Clinic (Private) (मोबाइल क्लिनिक (निजी)<br>12 other private facility (Specify) (मोबाइल क्लिनिक (निजी)<br>10 Others (Specify) (अन्य (निर्दिष्ट करें)
referred_from_other1 *(required)*	103.2 What type of place were you referred from? - Other Government facility (Specify)<br><br>आपको कैसी जगह से जाने को कहा गया था? - अन्य सरकारी(स्पष्टकरें)<br>*Question relevant when: selected(${referred_from_place} , '11')*	
referred_from_other2 *(required)*	103.3 What type of place were you referred from? - Other Private facility (Specify)<br><br>आपको कैसी जगह से जाने को कहा गया था? -मोबाइल क्लिनिक Private/अन्य निजी (स्पष्टकरें)<br>*Question relevant when: selected(${referred_from_place} , '12')*	
referred_from_other3 *(required)*	103.4 What type of place were you referred from? - Other (Specify)<br><br>आपको कैसी जगह से जाने को कहा गया था? - अन्य निजी(स्पष्टकरें)<br>*Question relevant when: selected(${referred_from_place} , '10')*	
referred_to_place *(required)*	104.1 What type of place were you referred to?<br><br>आपको कौन सी दूसरी जगह जाने के लिए कहा गया था?<br>*Question relevant when: selected(${deli_referred} , '1')*	1 Respondent's Home (अपना घर)<br>2 Someone else's home (किसी और का घर)<br>3 Community Health Centre (CHC) सामुदायिक स्वास्थ्य केंद्र (सीएचसी)<br>4 Primary Health Centre (PHC) प्राथमिक स्वास्थ्य केंद्र (पीएचसी)<br>5 Sub-centre (उप-केन्द्र)<br>6 Mobile clinic (Govt.) (मोबाइल क्लिनिक (सरकार)<br>11 other Govt. facility (Specify) (अन्य सरकार सुविधा (निर्दिष्ट करें)<br>7 Private Hospital/clinic (निजी अस्पताल / क्लिनिक)<br>8 Maternity Home (प्रसूति गृह)<br>9 Mobile Clinic (Private) (मोबाइल क्लिनिक (निजी)

		12 other private facility (Specify) (मोबाइल क्लिनिक (निजी)<br>10 Others (Specify) (अन्य (निर्दिष्ट करें)
referred_to_other1 *(required)*	104.2 What type of place were you referred to? - Other Government facility (Specify)<br><br>आपको कौन सी दूसरी जगह जाने के लिए कहा गया था? /अन्य सरकारी(स्पष्टकरें)<br>*Question relevant when: selected(${referred_to_place} , '11')*	
referred_to_other2 *(required)*	104.3 What type of place were you referred to? - Other Private facility (Specify)<br><br>आपको कौन सी दूसरी जगह जाने के लिए कहा गया था? - अन्य निजी (स्पष्टकरें)<br>*Question relevant when: selected(${referred_to_place} , '12')*	
ref_others *(required)*	What type of place were you referred to? (others)<br><br>आपको कौन सी दूसरी जगह जाने के लिए कहा गया था? - अन्य (स्पष्टकरें)<br>*Question relevant when: selected(${referred_to_place} , '10')*	
Social Support and Privacy During Labor and Delivery <br>सामाजिक सहायता और गोपनीयता<br>*Group relevant when: selected(${witness_consent} , '1')*		
accompany *(required)*	Now we will start with 'Social support and privacy during labor and delivery' section of the survey.<br><br>अब हम 'सामाजिक सहायता और गोपनीयता' अनुभाग में प्रवेश करेंगे ?<br><br>105 Did anyone accompany you from home to the health facility?<br><br>क्या कोई आपके साथ घर से स्वास्थ्य केंद्र आया था?	1 Yes (हाँ)<br>0 No (नहीं)
accompany_a *(required)*	106. Who accompanied you?<br><br>आपके साथ कौन आया था?<br>*Select all that apply*<br>*Question relevant when: selected(${accompany} , '1')*	1 Husband/Partner (पति/ साथी)<br>2 Mother-in-law (सास)<br>3 Mother (माँ)<br>4 Sister (बहन0<br>5 Friend/Neighbour (दोस्त/पडोसी)<br>6 Nurse/Midwife (नर्स/दाई0<br>7 Doctor (डॉक्टर)<br>9 Other (Specify) (अन्य (स्पष्टकरें))
accompany_specify *(required)*	106.2. Who accompanied you? - Other (Specify)<br><br>आपके साथ कौन गया था? अन्य (स्पष्टकरें)<br>*Question relevant when: selected(${accompany_a} , '9')*	
present_deli *(required)*	107. a. Did anyone stay with you in the delivery room?<br><br>क्या कोई आपके साथ पसब के कमरे में उपस्थित था ?	1 Yes (हाँ)<br>0 No (नहीं)
present_deli_want *(required)*	107. b. Did you want anyone to stay with you in the delivery room?<br><br>क्या आप चाहते थे की प्रसब के दौरान कोई आपके साथ उपस्थित रहे?	1 Yes (हाँ)<br>0 No (नहीं)
want_stay_deliveryroom *(required)*	108. Who did you want to stay in the delivery room with you?<br><br>आप प्रसब के समय उस कमरे में किसकी उपस्थिति चाहती थी?<br>*Select all that apply*<br>*Question relevant when: selected(${present_deli_want} , '1')*	1 Husband/Partner (पति/ साथी)<br>2 Mother-in-law (सास)<br>3 Mother (माँ)<br>4 Sister (बहन0<br>5 Friend/Neighbour (दोस्त/पडोसी)<br>6 Nurse/Midwife (नर्स/दाई0<br>7 Doctor (डॉक्टर)<br>9 Other (Specify) (अन्य (स्पष्टकरें))
deli_others_specify *(required)*	109.2 Who did you want to stay in the delivery room with you? Other (Specify)<br><br>आप प्रसब के समय उस कमरे में किसकी उपस्थिति चाहती थी? अन्य (स्पष्टकरें)<br>*Question relevant when: selected(${want_stay_deliveryroom} , '9')*	
broughtfood *(required)*	110. "Now I am going to ask you a few questions about other types of support…" Please let me know who, if anyone, provided each form of support. Feel free to say "no one" if no one provided you with the support<br><br><br>"अब मैं आपसे अन्य प्रकार कि सहयता के बारे में कुछ प्रश्न पूछने जा रही हूं …" कृपया मुझे बताएं कि, अगर कोई है, तो वो प्रत्येक प्रकार की सहयता प्रदान करते है। अगर कोई आपको सहयता प्रदान नहीं करता है तो आप"कोई नहीं" कहने के लिए स्वतंत्र महसूस करें| 68.1 Who brought you food or water before/after delivery?	1 Mother (माँ)<br>2 Mother-in-law (सास)<br>3 Husband/Partner (पति/ साथी)<br>4 Sister (बहन)<br>5 Friend/Neighbour (दोस्त/पडोसी)<br>6 Other health worker (अन्य स्वास्थ्य कार्यकर्ता)

	प्रसव के पहले/बाद कौन आपके लिए पानी या खाना लाए ?<br>*Probe- Anyone else*	7 ASHA (आशा)<br>10 No one (कोई नहीं)<br>9 Other (Specify) अन्य (स्पष्टकरें)
bring_food_other *(required)*	110.2 Who brought you food or water before/after delivery? - Other (Specify)<br><br>प्रसव के पहले/बाद कौन आपके लिए पानी या खाना लाए ? अन्य<br>*Question relevant when: selected(${broughtfood} , '9')*	
helped_speak *(required)*	111. Who helped you speak to the provider?<br><br>स्वास्थ्य प्रदाताओं के साथ बात करने में किसने मदद की<br>*Select all that apply <br>Probe: Anyone else?*	1 Mother (माँ)<br>2 Mother-in-law (सास)<br>3 Husband/Partner (पति/ साथी)<br>4 Sister (बहन)<br>5 Friend/Neighbor (दोस्त/पडोसी)<br>6 Other health worker (अन्य स्वास्थ्य कार्यकर्ता)<br>7 ASHA (आशा)<br>10 No one (कोई नहीं)<br>9 Other (Specify) अन्य (स्पष्टकरें)
talk_hp_specify *(required)*	111.2 Who helped you speak to the provider? - Other (Specify)<br><br>स्वास्थ्य प्रदाताओं के साथ बात करने में किसने मदद की - अन्य<br>*Question relevant when: selected(${helped_speak} , '9')*	
information *(required)*	112.1 Gave you information about what was happening with you or your baby<br><br>आप और आपके बच्चे के साथ क्या हो रहा है उसके बारे में आपको किसने जानकारी दी<br>*Probe: Anyone else?*	1 Mother (माँ)<br>2 Mother-in-law (सास)<br>3 Husband/Partner (पति/ साथी)<br>4 Sister (बहन)<br>5 Friend/Neighbor (दोस्त/पडोसी)<br>6 Other health worker (अन्य स्वास्थ्य कार्यकर्ता)<br>7 ASHA (आशा)<br>10 No one (कोई नहीं)<br>9 Other (Specify) अन्य (स्पष्टकरें)
information_specify *(required)*	112.2 Gave you information about what was happening with you or your baby - Other (Specify)<br><br>आप और आपके बच्चे के साथ क्या हो रहा है उसके बारे में जानकारी दी गई?- अन्य<br>*Question relevant when: selected(${information} , '9')*	
laborsuprt *(required)*	113.1 Provided support (with labor, breathing, techniques, strategies)<br><br>प्रसव पीड़ा के दौरान सांस लेने की तकनीक में किशने आप को सहायता प्रदान की<br>*Probe: Anyone else?*	1 Mother (माँ)<br>2 Mother-in-law (सास)<br>3 Husband/Partner (पति/ साथी)<br>4 Sister (बहन)<br>5 Friend/Neighbor (दोस्त/पडोसी)<br>6 Other health worker (अन्य स्वास्थ्य कार्यकर्ता)<br>7 ASHA (आशा)<br>10 No one (कोई नहीं)<br>9 Other (Specify) अन्य (स्पष्टकरें)
laborsuprt_specify *(required)*	113.2 Provided support (with labor, breathing, techniques, strategies) - Other (Specify)<br><br>सहायता प्रदान की (प्रसव पीड़ा के दौरान, सांस लेने में, तकनीक - अन्य<br>*Question relevant when: selected(${laborsuprt} , '9')*	
encourage *(required)*	114.1 Provided encouragement or guidance<br><br>प्रोत्साहन और मार्गदर्शन प्रदान किया<br>*Probe- "anyone else"*	1 Mother (माँ)<br>2 Mother-in-law (सास)<br>3 Husband/Partner (पति/ साथी)<br>4 Sister (बहन)<br>5 Friend/Neighbor (दोस्त/पडोसी)<br>6 Other health worker (अन्य स्वास्थ्य कार्यकर्ता)<br>7 ASHA (आशा)<br>10 No one (कोई नहीं)<br>9 Other (Specify) अन्य (स्पष्टकरें)
encourage_specify *(required)*	114.2 Provided encouragement or guidance - Other (Specify)	

	प्रोत्साहन और मार्गदर्शन प्रदान किया | अन्य<br>*Question relevant when: selected(${encourage} , '9')*	
curtains *(required)*	115. Were there curtains, a wall, or something else separating you and other women in the labor room?<br><br>क्या लेबर रूम में पर्दे, दीवार, या कुछ और था जो आपको अन्य महिलाओं से अलग कर रहे थे?	1 Yes (हाँ)<br>0 No (नहीं)<br>88 Don't Know (पता नहीं)
Pregnancy and Childbirth History – Delivery Care for Live Birth or Still Birth <br>गर्भावस्था और प्रसव के इतिहास - लाइव जन्म या फिर भी जन्म के लिए डिलिवरी देखभाल<br>*Group relevant when: selected(${witness_consent} , '1')*		
labor_hr *(required)*	Now we will start with your experience of 'Delivery care' at the facility<br><br>अब हम 'प्रसव के बाद दी गयी देखभाल' अनुभाग में प्रवेश करेंगे ?<br><br>116. How long were you in labor before you reached the health facility?<br><br>स्वाथ्य केंद्र पहुंचने से पहले आप कितने समय प्रसव पीड़ा में थीं?	1 Less than an hour (एक घंटे से कम)<br>2 One or more than one hour (एक या एक घंटे से अधिक)
labor_hrs *(required)*	117. How many hours?<br><br>कितने घंटे लगें ?<br>*Question relevant when: selected(${labor_hr} , '2')*<br>*Response constrained to: .>=1 and .<=48*	
test_wait_time_enter *(required)*	118 When you arrived in the health facility, about how long did you wait before you were first examined by a health provider?<br><br>जब आप स्वास्थ्य केंद्र में पहुंची, स्वास्थ्य प्रदाता से पहली बार जांच कराने के लिए आपको कितना समय इंतज़ार करना पड़ा ?<br>*Response constrained to: .>=0 and .<=48*	
wait_time_period_unit *(required)*	119. Select Hours/minutes for the response entered in the previous question<br><br>ऊपर भरी अवधि की इकाई बताएं|	1 Minutes (मिनट)<br>2 Hours (घंटे)
del_asssited_ *(required)*	120.1 Who assisted with the delivery of your baby?<br><br>बच्चे के जन्म के दौरान आपको किसने सहायता प्रदान की?<br>*Select all that apply<br><br>Probe: Anyone else?*	1 Doctor (डॉक्टर)<br>2 Nurse (नर्स)<br>3 Auxilary Nurse Midwife (A.N.M.) (ए.एन.एम)<br>4 Midwife (दाई)<br>5 Accredited Social Health Activist (ASHA)/Anganwadi Worker (AWW) (आशा / आगनवाड़ी करीयाकर्ता)<br>6 Family members/Friend (परिवार के लोग / दोस्त)<br>7 Don't Know (पता नहीं)<br>9 Others (अन्य)
del_asssited_specify *(required)*	120.2 Who assisted with the delivery of your baby? - Other<br><br>बच्चे के जन्म के दौरान आपको किसने सहायता प्रदान की? - अन्य<br>*Question relevant when: selected(${del_asssited_} , '9')*	
provider *(required)*	121. Was the main provider a man or woman?<br><br>मुख्य प्रदाता आदमी या औरत थी ?<br>*Only select one (MAIN)*	1 Male (पुरूष)<br>2 Female (औरत)
"procedures during labor"<br><br>गर्भावस्था के दौरान प्रक्रियाओं।<br>*Group relevant when: selected(${witness_consent} , '1')*		
hp_ask_complications *(required)*	I am now going to ask you several questions about services you received or did not receive WITHIN THE FIRST HOUR OF YOUR ARRIVAL AT THE HEALTH FACILITY.<br><br>अब मैं आपको प्राप्त सेवाओं के बारे में कई प्रश्न पूछने जा रहा हूं या प्राप्त नहीं हुआ है| स्वास्थ्य सुविधा पर आपके आगमन के पहले घंटे के भीतर।<br><br>122. Did the health provider ask you questions about how you were feeling or about any problems you were having?<br><br>क्या किसी स्वास्थ्य सहयोगी ने आपसे आपकी तबीयत के बारे में पूछा/ या आपसे पुछा यदि आपको कोई कठिनाई हो रही है?	1 Yes (हाँ)<br>0 No (नहीं)<br>88 Don't Know (पता नहीं)
hpask_headache_visionprob *(required)*	123. Did a health provider ask if you had experienced headaches or blurred vision?	1 Yes (हाँ)<br>0 No (नहीं)

	क्या किसी स्वास्थ्य सहयोगी ने आपसे पुछा कि क्या आपको सर दर्द हो रहा है, या आपकी दृष्टि धुंधली है?	88 Don't Know (पता नहीं)
hpask_bleeding *(required)*	124.Did a health provider ask if you had experienced vaginal bleeding?<br><br>क्या किसी स्वास्थ्य सहयोगी ने आपसे पुछा कि क्या आपके योनि से खून निकल रहा है?	1 Yes (हाँ)<br>0 No (नहीं)<br>88 Don't Know (पता नहीं)
hpask_water *(required)*	125. Did a health provider ask if your water had broken and for how long?<br><br>क्या किसी स्वास्थ्य सहयोगी ने आपसे पुछा कि क्या आपको पानी चल रहा है और कब से?	1 Yes (हाँ)<br>0 No (नहीं)<br>88 Don't Know (पता नहीं)
hpask_questions *(required)*	126. Did a health provider ask if you had any questions?<br><br>क्या किसी स्वास्थ्य सहयोगी ने आपसे पुछा कि क्या आपके पास उन के लिए कोई प्रश्न हैं?	1 Yes (हाँ)<br>0 No (नहीं)<br>88 Don't Know (पता नहीं)
hpcheck_bp *(required)*	127. Did a health provider check your blood pressure?<br><br>क्या किसी स्वास्थ्य सहयोगी ने आपके खून के दबाव (ब्लडप्रेशर) की जाँच की थी?	1 Yes (हाँ)<br>0 No (नहीं)<br>88 Don't Know (पता नहीं)
hpcheck_pulse *(required)*	128. Did a health provider check your pulse rate?<br><br>क्या किसी स्वास्थ्य सहयोगी ने आपके नाड़ी (पल्स) की जाँच की थी?	1 Yes (हाँ)<br>0 No (नहीं)<br>88 Don't Know (पता नहीं)
hpcheck_contraction *(required)*	129. Did a health provider time your contractions?<br><br>क्या किसी स्वास्थ्य सहयोगी ने आपके संकुचन (कंट्रक्शन्स) का जाँच किया था?	1 Yes (हाँ)<br>0 No (नहीं)<br>88 Don't Know (पता नहीं)
hpcheck_heartbeat *(required)*	130. Did a health provider check your baby's heart beat?<br><br>क्या किसी स्वास्थ्य सहयोगी ने आपके बच्चे के दिल की धड़कन की जाँच की थी?	1 Yes (हाँ)<br>0 No (नहीं)<br>88 Don't Know (पता नहीं)
hpcheck_vagina *(required)*	131. Did a health provider perform a vaginal examination on you?<br><br>क्या किसी स्वास्थ्य सहयोगी ने आपकी योनि की जाँच की थी?	1 Yes (हाँ)<br>0 No (नहीं)<br>88 Don't Know (पता नहीं)
waittime_deli *(required)*	I would now like to ask you questions about procedures you received during labour. Please answer apart from your initial examination.<br><br>अब मैं आपको श्रम के दौरान प्राप्त प्रक्रियाओं के बारे में प्रश्न पूछना चाहूंगा। कृपया अपनी प्रारंभिक परीक्षा के अलावा उत्तर दें।<br><br>132.1 About how long were you in the facility before you delivered your baby?<br><br>आपको स्वास्थ्य केंद्र आए कितना समय हुआ था जब आपको बच्चा हुआ ?	1 Less than an hour (एक घंटे से कम)<br>2 One or more than one hour (एक या एक घंटे से अधिक)
after_del_hrs *(required)*	132.2 Duration in hours?<br><br>कितने घंटे तक ?<br>*Question relevant when: selected(${waittime_deli} , '2')*<br>*Response constrained to: .>=1 and .<=99*	
leftalone_labour *(required)*	133. Were you left alone at any point during labour or delivery?<br><br>प्रसव पीड़ा या प्रसव के दौरान क्या कभी भी आपको अकेले छोड़ा गया था?	1 Yes (हाँ)<br>0 No (नहीं)<br>88 Don't Know (पता नहीं)
"procedures during labor.<br><br>गर्भावस्था के दौरान प्रक्रियाओं।<br>*Group relevant when: selected(${witness_consent} , '1')*		
blood_presr *(required)*	134. Apart from the intial examination when you arrived at the health facility, did a health provider at any other time check your blood pressure during your labour?<br><br>स्वास्थ्य केंद्र पहुचने के बाद प्रारम्भिक जाच के उपरांत क्या किसी स्वास्थ्य सहयोगी ने आपकी कभी भी प्रसव के समय बीपी (ब्लड प्रैशर) की जाच की ?	1 Yes (हाँ)<br>0 No (नहीं)<br>88 Don't Know (पता नहीं)
pulse *(required)*	135. Apart from the initial examination when you arrived at the health facility, did a health provider at any other time check your pulse rate during your labour?<br><br>स्वास्थ्य केंद्र पहुचने के बाद प्रारम्भिक जाच के उपरांत क्या किसी स्वास्थ्य सहयोगी ने आपकी कभी भी प्रसव के समय नाड़ी (पल्स) की जाच की ?	1 Yes (हाँ)<br>0 No (नहीं)<br>88 Don't Know (पता नहीं)
contraction *(required)*	136. Apart from the intial examination when you arrived at the health facility, did a health provider at any other time check your contraction during your labour?<br><br>स्वास्थ्य केंद्र पहुचने के बाद प्रारम्भिक जाच के उपरांत क्या किसी स्वास्थ्य सहयोगी ने आपकी कभी भी प्रसव के समय संकुचन (कंट्रक्शन्स) की जाच की ?	1 Yes (हाँ)<br>0 No (नहीं)<br>88 Don't Know (पता नहीं)
heartbeat *(required)*	137. Apart from the initial examination when you arrived at the health facility, did a health provider at any other time	1 Yes (हाँ)

	check your baby's heart beat during your labour?<br><br>स्वास्थ्य केंद्र पहुचने के बाद प्रारम्भिक जाच के उपरांत क्या किसी स्वास्थ्य सहयोगी ने आपकी कभी भी प्रसव के समय आपके बच्चे के दिल की धड़कन की जाँच की ?	0 No (नहीं)<br>88 Don't Know (पता नहीं)
vaginal_exami *(required)*	138. Apart from the initial examination when you arrived at the health facility, did a health provider at any other time perform vaginal examination on you during your labour?<br><br>स्वास्थ्य केंद्र पहुचने के बाद प्रारम्भिक जाच के उपरांत क्या किसी स्वास्थ्य सहयोगी ने प्रसव के समय आप की योनि की जाँच की?	1 Yes (हाँ)<br>0 No (नहीं)<br>88 Don't Know (पता नहीं)
Pregnancy and Childbirth History – Procedures during delivery <br>(गर्भावस्था और प्रसव के इतिहास - प्रसव के दौरान प्रक्रियाएं)<br>*Group relevant when: selected(${witness_consent} , '1')*		
other_prob *(required)*	"Now we will start with 'procedures during delivery' section of the survey.<br><br>139. At any time just during the delivery did you suffer from any problems?<br><br>क्या किसी भी समय प्रसव के दौरान आपको अन्य कोई समस्या हुई थी?	1 Yes (हाँ)<br>0 No (नहीं)<br>88 Don't Know (पता नहीं)
deliprob *(required)*	140. IF YES: What problems did you have? Anything else?<br><br>आपको कौन कौन सी समस्याएं हुई थी? अन्य कोई?<br>*Select all that apply<br><br>Probe: Anything else?*<br>*Question relevant when: selected(${other_prob} , '1')*	1 Headache (सरदर्द)<br>2 Blurry vision (धुंधली दृष्टि)<br>3 Edema/Pre-eclamsia (सूजन)<br>4 Vaginal Bleeding (योनि से खून बहना)<br>5 Convulsions/eclampsia (दौरे पड़ना)<br>6 Tetanus (धनुस्तंभ(टिटनेस))<br>7 Foul-smelling discharge (बदबूदार श्राव)<br>8 Lower abdominal pain (निचले पेट में दर्द)<br>10 Fever (बुखार)<br>11 Excessive vomiting (अत्यधिक उलटी)<br>12 Dizziness (चक्कर आना)<br>13 Palpitation (घबराहट)<br>14 High blood pressure (उच्च रक्त चाप)<br>15 Diabetes (सूगर)<br>16 Anaemia (एनीमिया (खून की कमी))<br>17 Malaria (मलेरिआ)<br>18 Urinary Tract Infection (मूत्र पथ के संक्रमण)<br>19 Slow or no baby movement (धीमी गति या बच्चे का कोई हलचल नहीं)<br>20 Baby's head/feet came out first (शिशु के हाथ / पैर पहले बाहर आया)<br>21 Prolonged labor (लंबे समय की प्रसव पीड़ा)<br>22 Obstructed labor (बाधित प्रसव)<br>23 Torn uterus (फटे गर्भाशय)<br>24 Placenta Previa (प्लेसेंटा प्रेविया)<br>25 Fistula (नासूर)<br>9 Other (Specify) (अन्य (स्पष्ट करें))
deliprob_specify *(required)*	140.2 IF YES: What problems did you have? Anything else? - Other (specify)<br><br>आपको कौन कौन सी समस्याएं हुई थी? अन्य कोई? - अन्य (स्पष्ट करें)<br>*Question relevant when: selected(${deliprob} , '9')*	
intravenous_fluids *(required)*	141. Did you receive any of the following during labor or delivery? - Intravenous fluids<br><br>प्रसव पीड़ा या प्रसव के दौरान आपको निम्नलिखित में से क्या प्राप्त हुआ था? - Intravenous fluids (IV)/इंट्रावेनस फ्लूइड	1 Yes (हाँ)<br>0 No (नहीं)<br>88 Don't Know (पता नहीं)
bloodtransfusion *(required)*	142. Did you receive any of the following during labor or delivery? - blood transfusion	1 Yes (हाँ)<br>0 No (नहीं)

	प्रसव पीड़ा या प्रसव के दौरान आपको निम्नलिखित में से क्या प्राप्त हुआ था? रक्त ट्रांस्फुसीओं (blood transfusion)	88 Don't Know (पता नहीं)
receive_pain *(required)*	143. Did you receive any of the following during labor or delivery? - pain medications<br><br>प्रसव पीड़ा या प्रसव के दौरान आपको निम्नलिखित में से क्या मिला था? - दर्द की दवाई	1 Yes (हाँ)<br>0 No (नहीं)<br>88 Don't Know (पता नहीं)<br>77 Medicine not available (मेडिसिन उपलब्ध नहीं है)
pain_other *(required)*	144. Did you receive any of the following during labor or delivery? - other medications besides pain medications<br><br>प्रसव पीड़ा या प्रसव के दौरान आपको निम्नलिखित में से क्या प्राप्त हुआ था? - दर्द की दवाई के इलावा कोई और दवाई	1 Yes (हाँ)<br>0 No (नहीं)<br>88 Don't Know (पता नहीं)
intrument *(required)*	145. Were Instruments used to get your baby out (Forceps)<br><br>क्या आपके बच्चे को किसी उपकरण की सहायता के साथ निकाला गया था?	1 Yes (हाँ)<br>0 No (नहीं)<br>88 Don't Know (पता नहीं)
cesarian *(required)*	146. Was your baby delivered by cesarean section?<br><br>क्या आपका बच्चा शल्यक्रिया (cesarean section) के द्वारा हुआ था?	1 Yes (हाँ)<br>0 No (नहीं)<br>88 Don't Know (पता नहीं)
premature *(required)*	147. Was your baby delivered before 9 months of pregnancy?<br><br>क्या आपका बच्चा 9 महीने से पहले हुआ था ?	1 Yes (हाँ)<br>0 No (नहीं)<br>88 Don't Know (पता नहीं)
weight_baby *(required)*	148. How much did your baby weigh at the time of birth? (kilogram)<br><br>बच्चे का वज़न कितना था ? (किलोग्राम)	
kangarucare *(required)*	149. Was your baby put on your abdomen or chest as soon as it was born?<br><br>बच्चा होने के तुरंत बाद क्या उसे आपके पेट या छाती पर रखा गया था?<br>*Select Not Applicable if stillbirth.*	1 Yes (हाँ)<br>0 No (नहीं)<br>88 Don't Know (पता नहीं)<br>9 Not Applicable (लागू नहीं)
hp_checked *(required)*	"now I have some questions that relate to after your delivery"<br>(अब मेरे पास कुछ प्रश्न हैं जो आपकी डिलीवरी के बाद संबंधित हैं")<br><br>150. After your baby was born, did any health care provider check on your health?<br><br>बच्चा होने के बाद क्या किसी स्वास्थ्य सहयोगी ने आपके स्वास्थ्य की जाँच की थी?	1 Yes (हाँ)<br>0 No (नहीं)<br>88 Don't Know (पता नहीं)
first_checkup *(required)*	151. How long after delivery did the first check take place?<br><br>बच्चा होने के कितने समय बाद स्वास्थ्य सहयोगी ने आपके स्वास्थ्य की पहले बार जाँच की थी?<br>*Question relevant when: selected(${hp_checked} , '1')*	
wait_time1 *(required)*	151.2 Duration in hours/days/weeks<br>*Question relevant when: selected(${hp_checked} , '1')*	1 Hours (घंटे)<br>4 Minutes (मिनट)<br>2 Days (दिन)<br>3 Weeks (सप्ताह)<br>88 Don't know (पता नहीं)
Pregnancy and Childbirth History – Procedures after delivery<br><br>(गर्भावस्था और प्रसव के इतिहास - प्रसव के बाद प्रक्रियाएं)<br>*Group relevant when: selected(${witness_consent} , '1')*		
afterhrs_bp *(required)*	"""Now I have some questions that relate to AFTER your delivery"."<br><br>" अब मेरे पास आपके लिए कुछ प्रश्न हैं जो आपकी डिलीवरी से संबंधित हैं। "<br><br>152.1. Within two hours of delivery did a health provider check your blood pressure?<br><br>बच्चा होने के दो घंटे के अंदर क्या स्वास्थ्य सहयोगी ने खून के दबाव (ब्लडप्रेशर) की जाँच की<br>*Select N/A if stillbirth.*	1 Yes (हाँ)<br>0 No (नहीं)<br>88 Don't Know (पता नहीं)<br>9 Not Applicable (लागू नहीं)
after2hrs_pulse *(required)*	153. Within two hours of delivery did a health provider check your pulse?<br><br>बच्चा होने के दो घंटे के अंदर क्या स्वास्थ्य सहयोगी ने नाड़ी (पल्स) की जाँच की	1 Yes (हाँ)<br>0 No (नहीं)<br>88 Don't Know (पता नहीं)
after2hrs_abdomen *(required)*	154. Within two hours of delivery did a health provider check your abdomen?<br><br>बच्चा होने के दो घंटे के अंदर क्या स्वास्थ्य सहयोगी ने पेट की जाँच की	1 Yes (हाँ)<br>0 No (नहीं)<br>88 Don't Know (पता नहीं)
after2hrs_perineum *(required)*	155. Within two hours of delivery did a health provider check your perineum [area between vaginal opening and anus]?<br><br>बच्चा होने के दो घंटे के अंदर क्या स्वास्थ्य सहयोगी ने पेरिनुएम (गुदा और योनि मुख के बीच का भाग/ Perineum) की जाँच की ?	1 Yes (हाँ)<br>0 No (नहीं)<br>88 Don't Know (पता नहीं)

check_pad *(required)*	156. Within two hours of delivery did a health provider check your pad for amount of bleeding?<br><br>बच्चा होने के दो घंटे के अंदर क्या स्वास्थ्य सहयोगी ने पैड (Pad) की जाँच की ये पता करने के लिए की कितना खून बहा है|	1 Yes (हाँ)<br>0 No (नहीं)<br>88 Don't Know (पता नहीं)
child_test *(required)*	157. Within two hours of delivery did a health provider examine your baby?<br><br>बच्चा होने के दो घंटे के अंदर क्या स्वास्थ्य सहयोगी ने बच्चे की जाँच किया ?<br>*Select Not Applicable if stillbirth.*	1 Yes (हाँ)<br>0 No (नहीं)<br>88 Don't Know (पता नहीं)<br>9 Not Applicable (लागू नहीं)
breastfeed_well *(required)*	158. Within two hours of delivery did a health provider check if breastfeeding is going well?<br><br>बच्चा होने के दो घंटे के अंदर क्या स्वास्थ्य सहयोगी ने जाचा की बच्चा माँ का दूध सही तरह पी रहा है ?<br>*Select Not Applicable if stillbirth.*	1 Yes (हाँ)<br>0 No (नहीं)<br>88 Don't Know (पता नहीं)<br>9 Not Applicable (लागू नहीं)
Pregnancy and Childbirth History - Postpartum care<br><br>Hint: (These questions still relate to after your delivery)<br>*Group relevant when: selected(${witness_consent} , '1')*		
separate_bed *(required)*	159. Did you get a separate bed in the post-partum ward?<br><br>क्या आपको प्रसवोत्तर वार्ड में अलग बिस्तर मिला था ?	1 Yes (हाँ)<br>0 No (नहीं)<br>88 Don't Know (पता नहीं)
curtains_available *(required)*	160. Were there curtains in the ward where you were after delivery?<br><br>आप प्रसव के बाद जिस कमरे मैं थी क्या वहाँ पर्दे थे?	1 Yes (हाँ)<br>0 No (नहीं)<br>88 Don't Know (पता नहीं)
male_enter *(required)*	161. Could males enter freely in the ward?<br><br>क्या पुरुष वार्ड में स्वतंत्र रूप से प्रवेश कर सकते थे?	1 Yes (हाँ)<br>0 No (नहीं)<br>88 Don't Know (पता नहीं)
electricity_available *(required)*	162. Was there provision for electricity 24*7?<br><br>क्या वहां 24 * 7 बिजली की व्यवस्था थी?	1 Yes (हाँ)<br>0 No (नहीं)<br>88 Don't Know (पता नहीं)
water_available *(required)*	163. Was there provision for water supply 24*7 in bathroom?<br><br>क्या वहां बाथरूम मैं पानी की आपूर्ति के लिए 24 * 7 व्यवस्था थी?	1 Yes (हाँ)<br>0 No (नहीं)<br>88 Don't Know (पता नहीं)
food_provided *(required)*	164. Was food provided by the facility?<br><br>स्वास्थ्य केन्द्र में खाने कि व्यबस्था थी?	1 Yes (हाँ)<br>0 No (नहीं)<br>88 Don't Know (पता नहीं)
doc_visit_times *(required)*	165. How frequently did doctor visit you in the ward?<br><br>चिकित्सक वार्ड में आप की जाँच के लिए कितने बार आते थे?	0 Never (कभी नहीँ)<br>1 Once a day (दिन में एक बार)<br>2 Twice a day (दिन में दो बार)<br>3 Thrice a day (दिन में तीन बार)<br>4 More than thrice a day (दिन में तीन बार से ज़्यादा)
nurse_visit_times *(required)*	166. How frequently did nurse visit you at post-natal ward?<br><br>नर्स आपको देखने के लिए प्रसवोत्तर वार्ड मैं कितने बार आती थी?	0 Never (कभी नहीँ)<br>1 Once a day (दिन में एक बार)<br>2 Twice a day (दिन में दो बार)<br>3 Thrice a day (दिन में तीन बार)<br>4 More than thrice a day (दिन में तीन बार से ज़्यादा)
staff_availability *(required)*	167. When you needed whether any staff was available for assistance?<br><br>क्या आपके जरूरत के समय कोई भी कर्मचारी सहायता के लिए उपलब्ध रहते थे?	1 Yes (हाँ)<br>0 No (नहीं)<br>88 Don't Know (पता नहीं)
planning_fp	168. Are you planning to use any family planning method in the next 6 months?<br><br>क्या आप अगले 6 महीने में कोई भी परिवार नियोजन की विधि उपयोग करने की योजना बना रहे ही ?	1 Already using one (पहले से ही उपयोग कर रहे है)<br>2 Planning to use (इस्तेमाल की योजना कर रहे है)<br>3 Not planning (अभी सोचा नही है)<br>88 Don't know (पता नहीं)
coper_t_operation	169. Did you undergo copper-t insertion or sterilization after your delivery?<br><br>क्या प्रसव के बाद आपको कॉपर-टी लगाया गया या आपका ऑपरेशन किआ गया ?	0 No (नही)<br>1 Sterilization (बंध्याकरण)<br>2 Intrauterine Device (IUD) (आई यू डी)
method_next6m	170. In the next 6 months which family planning method are you planning to use? - Specify<br><br>आप 6 महीने में कौन सी तकनीक अपनाने वाली हैं, स्पस्ट करें |	
consent_iud_ster	171. When you underwent family planning procedure (IUD/sterilization), did the staff take your consent?	1 Yes (हाँ)

	जब आप परिवार नियोजन प्रक्रिया (आईयूडी / नसबंदी) लेते थे, तो क्या कर्मचारी आपकी सहमति लेते थे?	0	No (नहीं)
		88	Don't Know (पता नहीं)
Pregnancy and Childbirth History – Discharge<br><br>गर्भावस्था और प्रसव इतिहास - निर्वहन<br>*Group relevant when: selected(${witness_consent} , '1')*			
anycompli_afterdeli *(required)*	172. At any time just after the delivery did you suffer from any problems?<br><br>बच्चा होने के एकदम बाद क्या आपको किसी प्रकार की कठिनाइयों का सामना करना पड़ा था?	1	Yes (हाँ)
		0	No (नहीं)
		88	Don't Know (पता नहीं)
comp_afterdelivery *(required)*	173.1. IF YES: What problems did you have?<br><br>आपको कौन कौन सी समस्याएं हुई थी<br>*Select all that apply*<br>*Question relevant when: selected(${anycompli_afterdeli} , '1')*	1	Headache (सरदर्द)
		2	Blurry vision (धुंधली दृष्टि)
		3	Edema/Pre-eclamsia (सूजन)
		4	Vaginal Bleeding (योनि से खून बहना)
		5	Convulsions/eclampsia (दौरे पड़ना)
		6	Tetanus (धनुस्तंभ(टिटनेस))
		7	Foul-smelling discharge (बदबूदार श्राव)
		8	Lower abdominal pain (निचले पेट में दर्द)
		10	Fever (बुखार)
		11	Excessive vomiting (अत्यधिक उलटी)
		12	Dizziness (चक्कर आना)
		13	Palpitation (घबराहट)
		14	High blood pressure (उच्च रक्त चाप)
		15	Diabetes (सूगर)
		16	Anaemia (एनीमिया (खून की कमी))
		17	Malaria (मलेरिआ)
		18	Urinary Tract Infection (मूत्र पथ के संक्रमण)
		19	Slow or no baby movement (धीमी गति या बच्चे का कोई हलचल नहीं)
		20	Baby's head/feet came out first (शिशु के हाथ / पैर पहले बाहर आया)
		21	Prolonged labor (लंबे समय की प्रसव पीड़ा)
		22	Obstructed labor (बाधित प्रसव)
		23	Torn uterus (फटे गर्भाशय)
		24	Placenta Previa (प्लेसेंटा प्रेविया)
		25	Fistula (नासूर)
		9	Other (Specify) (अन्य (स्पष्ट करें))
compli_afterdeli_specify *(required)*	173.2 IF YES: What problems did you have? - Other (Specify)<br><br>आपको कौन कौन सी समस्याएं हुई थी? अन्य (स्पष्ट करें)<br>*Question relevant when: selected(${comp_afterdelivery} , '9')*		
Household Characteristics: Before we finish, I would like to ask you some final questions about your household<br><br>अब हम 'घर की विशेषताये' अनुभाग में प्रवेश करेंगे ?<br>*Group relevant when: selected(${witness_consent} , '1')*			
hh_adult	174. How many people live in your household, including yourself? How many adults?<br><br>आपके घर में कितने बड़े लोग है ? (adults)<br>*Response constrained to: .>=1 and .<=20*		
hh_children	175. How many children live in your household?<br><br>आपके घर में कितने बच्चे है ?<br>*Response constrained to: .>=1 and .<=20*		
living *(required)*	176. Do any of the following people stay with you."<br><br>क्या आप के घर में आप के साथ इनमे से कोई रहते हैं ?	1	Husband/Male partner (पति / पुरुष साथी)
		2	your mother (आपकी मां)

	Select all that apply	3 Your father (आपकी पिताजी)<br>4 Mother in-law (सास)<br>5 Father-in-Law (ससुर)<br>6 Your siblings (आपके भाई बहन)<br>7 bother- in law (बहनोई/ देवर)<br>8 Sister-in law (ननद)
household_goods	177.1 Does your household have a (name)<br><br>क्या आपके घर में (नाम) वस्तुये है<br>*Select all that apply*	1 Pressure Cooker (प्रेशर कुकर)<br>2 Color Television (रंगीन टेलीविजन)<br>3 Table (टेबुल)<br>5 Chair (कुरसी)<br>4 Electric Fan (बिजली का पंखा)<br>6 Refrigerator (फ्रिज)<br>7 Mattress (गद्दा)<br>8 None of the above (इनमे से कोई नही)
hh_window_glass	178. Does your household have windows with glass?<br><br>क्या आपके घर में शीशे की खिड़कियां है?	1 Yes (हाँ)<br>0 No (नहीं)
hh_toilet1	179. What type of toilet does your household use?<br><br>आप लोग घर पे किस प्रकार की शौचालय का उपयोग करते है?	1 Any type of latrine/toilet<br>2 Bush/no facility
hh_roof	180. What is the main material that the roof of your household is made of?<br><br>आपके घर की छत बनाने के लिए कौनसा मुख्य सामग्री का प्रयोग किआ गया है ?	1 Concrete/Cement roof<br>2 Any other material
hh_wall	181. What is the main material that the walls of your household are made of?<br><br>आपके घर की दीवारों को बनाने के लिए कौनसा मुख्य सामग्री का प्रयोग किआ गया है ?	1 Cement wall<br>2 Any other material
hh_cooking	182. What type of fuel does your household use for cooking?<br><br>खाना पकाने के लिए आपके घर में कौन से प्रकार का ईंधन का उपयोग किआ जाता है?	1 LPG, Natural gas for cooking<br>2 Wood<br>9 Others
hh_other	182.2. What type of fuel does your household use for cooking? - Other<br><br>खाना पकाने के लिए आपके घर में कौन से प्रकार का ईंधन का उपयोग किआ जाता है? - अन्य<br>*Question relevant when: selected(${hh_cooking} , '9')*	
hh_account	183. Does any member of your household have a bank account or post office account?<br><br>आपके घर के किसी भी सदस्य का बैंक खाता या डाकघर खाता है?	1 Yes (हाँ)<br>0 No (नहीं)
end_interv *(required)*	end_interview<br>*This is the end of the interview. Thank you for your time.<br><br><br>(यह साक्षात्कार का अंत है आपके समय के लिए शुक्रिया।)*<br>*Question relevant when: selected(${witness_consent} , '1')*	
