# Supplementary material for: A comparison of intensive vs. light-touch quality improvement interventions for maternal health in Uttar Pradesh, India
Source: BMC Health Serv Res. 2020 Dec 4;20:1121. doi: 10.1186/s12913-020-05960-6 (PMC7716449; doi:10.1186/s12913-020-05960-6)
Supplement: Supplementary file 6 — Additional file 6. [file 12913_2020_5960_MOESM6_ESM.docx]

Supplement 1. Sensitivity Analyses: comparison of PCMC score & Change package results (adjusted) with propensity score matching (PSM).

|  |  | | |  |
| --- | --- | --- | --- | --- |
|  | **Survey Round: Endline (reference Baseline)** | **Treatment Group: Light Touch (reference Intensive Intervention)** | **Interaction term** | **R^2^** |
| **Full PCMC score, 23 items (adjusted), original** |  |  |  |  |
| Coefficient | 9.16 | -23.27 | 16.15 |  |
| 95%CI | (7.50, 10.83) | (-25.02, -21.51) | (13.47, 18.83) | 0.7351 |
| p-value | 0.000 | 0.000 | 0.000 |  |
| **Full PCMC score (adjusted), using PSM** |  |  |  |  |
| Coefficient | 9.44 | -22.59 | 15.94 |  |
| 95%CI | (7.38, 11.51) | (-24.65, -20.54) | (12.44, 19.44) | 0.7020 |
| p-value | 0.000 | 0.000 | 0.000 |  |
| **Change Package score, 8 items (adjusted)** |  |  |  |  |
| Coefficient | 26.94 | -25.52 | 11.75 |  |
| 95%CI | (24.03, 29.85) | (-28.33, -22.70) | (7.33, 16.17) | 0.7609 |
| p-value | 0.000 | 0.000 | 0.000 |  |
| **Change Package score (adjusted), using PSM** |  |  |  |  |
| Coefficient | 25.37 | -25.33 | 14.95 |  |
| 95%CI | (22.02, 28,72) | (-28.53, -22.13) | (9.43, 20.47) | 0.7103 |
| p-value | 0.000 | 0.000 | 0.000 |  |
